# RBM10 Is a Biomarker Associated with Pan-Cancer Prognosis and Immune Infiltration: System Analysis Combined with In Vitro and Vivo Experiments

**DOI:** 10.1155/2022/7654937

**Published:** 2022-11-28

**Authors:** Yingyue Cao, Luyi Pang, Shi Jin

**Affiliations:** ^1^National Cancer Center/National Clinical Research Center for Cancer/Cancer Hospital and Shenzhen Hospital, Chinese Academy of Medical Sciences and Peking Union Medical College, Shenzhen 518116, China; ^2^Department of Biomedicine, Southern University of Science and Technology, Shenzhen 518000, China

## Abstract

RNA binding motif protein 10 (RBM10) is a splicing factor that has been reported to be involved in the occurrence and progression of multiple malignancies. However, the RBM10 involvement in pan-cancer immunotherapy is not clear. In here, we provide the first comprehensive assessment of the prognostic value and immunological function of RBM10 in human pan-cancer utilizing multiple public databases. Data reveal the aberrant RBM10 expression in most tumors, and its expression is positively or negatively linked with the clinical prognosis of various cancers, depending on the different types and subtypes of cancers. In most tumors, RBM10 mutations are frequently occurred, which is closely related to tumor progression. Moreover, our results also show that RBM10 is considerably linked with most of the immune checkpoint genes, tumor immune cell infiltration, tumor mutation burden, and microsatellite instability. Additionally, RBM10 is significantly positively correlated with the sensitivity of trametinib, 17-AAG, PD-0325901, RDEA119, cetuximab, and afatinib, indicating potential antagonism between RBM10 inhibitors and these antitumor drugs, and more likely to develop drug resistance. We also verify that downregulation of RBM10 enhances the malignant phenotype of lung adenocarcinoma cells using in *vitro* cell experiments, and in *vivo* animal experiments show that the overexpression of RBM10 reduces the growth of tumors. Furthermore, upregulating RBM10 greatly reduces the PD-L1 protein levels, while silencing RBM10 considerably enhances PD-L1 protein levels. Moreover, the overexpression of RBM10 decreases the protein stability of PD-L1. To sum up, our pan-cancer analysis indicates that RBM10 is a promising biomarker for prognosis and immunotherapy, which provides a new insight for cancer immunotherapy.

## 1. Introduction

Malignant tumors bring overwhelming pain to individuals [1] and also are currently the main cause of mortality around the globe [2]. Nearly 19.3 million new cases and 10 million deaths related to cancer were recorded globally in 2020, which is critically increasing the cancer burden around the world [3]. Tumor immunotherapy, such as immune checkpoint (ICP) inhibitors, has greatly changed the tumor treatment mode by improving the survival time of cancer patients [4]. Unfortunately, only a few people have benefited, and the treatment of cancer is still unsatisfactory [5]. Therefore, it is urgent to explore the pathogenesis of cancer and find more effective new biomarkers and potential immunotherapeutic targets for cancer patients.

RNA binding motif protein 10 (RBM10, also called S1-1) belongs to the RNA binding motif protein (RBP) family. It is first discovered in 1995 [6] and is mainly responsible for the splicing of precursor mRNA and posttranscriptional regulation [7]. The roles of RBM10 in regulating apoptosis, cell proliferation, metastasis, and other biological activities have been proved [6, 8]. Upregulation of RBM10 inhibits the growth of primary chondrocytes by inducing apoptosis and blocking cell migration as well as inducing apoptosis by reducing the Bcl-2 expression [9]. RBM10 suppresses the growth of colorectal cancer cells by inhibiting of the MDM2-TP53 feedback loop to activate TP53 [10]. In lung adenocarcinoma (LUAD), RBM10 decreases cell proliferation through inhibiting RAP1/AKT/CREB pathway [11]. Moreover, our recent study has also proved the potential of RBM10 as a tumor suppressor that has the ability to inhibit the metastasis and proliferation of LUAD by partially inactivating the Wnt/*β*-catenin pathway [12]. These reports support that RBM10 has an essential function as a tumor suppressor gene. Interestingly, some recent studies have highlighted the carcinogenic effect of RBM10 [13, 14]. The increased RBM10 expression is substantially positively linked with the enhanced disease invasion of metastatic melanoma [15]. As we all known, immunotherapy and targeted therapy have greatly improved the prognosis of cancer patients. The study by Pang et al. [8] have shown that the high RBM10 expression is positively linked with most immune cell infiltrates and the expressions of PD-1 and PD-L1 in hepatocellular carcinoma (HCC). In addition, a recent research confirms that the low RBM10 expression significantly increases the immune activity of LUAD and is negatively linked with CD8+ T cells having a tumor-suppressive effect, which makes it evident that RBM10 is linked with immune infiltration [16]. Most current studies about the role of RBM10 in cancer have been focused on an individual or limited to some specific tumor types. Therefore, it is particularly important to comprehensively and systematically analyze the prognosis value, biological functions, molecular mechanism of RBM10, and its involvement in tumor immune microenvironment in different types of human cancers to provide new insights and directions for clinical treatment of tumors.

Therefore, the current study is aimed at exploring RBM10 expression profiles and prognosis value in pan-cancer. We initially utilize The Cancer Genome Atlas (TCGA), Genotype-Tissue Expression (GTEx), Gene Expression Profiling Interactive Analysis 2 (GEPIA2), and Kaplan-Meier (KM) plotter web tools to investigate the RBM10 expression and its clinical prognosis in human pan-cancer. The cBioPortal and UALCAN databases are used to evaluate the mutation characteristics of RBM10 gene, DNA methylation, and the correlation with copy number variations (CNV) in tumors. Additionally, the link of the RBM10 expression with tumor-infiltrating immune cells (TIICs) and related-immune markers is discussed by using different databases, such as the Tumor Immune Estimation Resource (TIMER), SangerBox, and an integrated repository portal for tumor-immune system interactions (TISIDB). The relationship of the RBM10 expression with ESTIMATEScore, ICPs, TMB, and MSI genes is also explored. The Gene Set Enrichment Analysis (GSEA) database is utilized for analyzing the drug sensitivity and functional enrichment analysis of RBM10. Finally, in *vitro* and in *vivo* experiments are used to further verify the effect of the abnormal RBM10 expression on the proliferation and metastasis of LUAD cells, and the effect of upregulation or silencing of the RBM10 expression on the PD-L1 expression in LUAD cells is examined by western blotting. To sum up, the results of the present study can help to understand the prognostic value of RBM10 in a variety of cancer types and its role in the tumor immune microenvironment, providing a solid base for investigating the involvement of RBM10 in cancer immunotherapy in the future.

## 2. Materials and Methods

### 2.1. Analysis of RBM10 Gene Expression

All raw data obtaining GTEx and TCGA were taken from the UCSC Xena data center (http://xena.ucsc.edu/) to evaluate the expression level of RBM10 in various types of cancer. The differential expression of RBM10 in tumor, normal, and metastatic tissues was explored using a TNM plotter [17] (https://tnmplot.com/analysis/). The “Stage Plot” module of GEPIA2 (http://gepia2.cancer-pku.cn/#analysis) was utilized for evaluating the link between RBM10 expression and different stages of pan-cancer, which was visualized as a violin diagram. The abbreviations and meanings of the 33 tumor types were seen in Table S1.

### 2.2. Protein Levels

The Human Protein Atlas (HPA, https://www.proteinatlas.org/) was utilized for exploring RBM10's protein level in tumors and healthy tissues of people. In addition, the clinical proteomic tumor analysis consortium (CPTAC) dataset (http://ualcan.path.uab.edu/analysis-prot.html) was employed for finding the expression levels of RBM10's total proteins in various tumors and healthy tissues.

### 2.3. Prognostic Analysis

Based on RBM10's median expression level, affected people were sorted into high and low expression groups. By the “Survival Map” module of GEPIA2, a heat map was generated to evaluate the link between RBM10 expression and cancer prognosis, obtaining overall survival (OS) and disease-free survival (DFS). The KM curves were drawn. Furthermore, the Kaplan-Meier plotter database (https://kmplot.com/analysis/) was employed for analyzing the link between the expression of RBM10 in the TCGA database and OS and relapse-free survival (RFS) of different cancers. Finally, the COX_OS, COX_DFI, COX_PFI, and COX_DSS analysis data of various tumors were obtained through SangerBox's “Gene-KM plotter” module (http://past20.sangerbox.com/Gene). The results were visualized as forest maps. *p* < 0.05 showed statistical significance.

### 2.4. Gene Alterations and DNA Methylation Level

The genetic alterations of RBM10 gene in pan-cancer were described through the “Cancer Type Summary” module from the cBioPortal website (https://www.cbioportal.org/). The “mutations” module provided us with the site map of RBM10. The OS, DFS, DSS, and PFS of patients with or without RBM10 changes in TCGA were analyzed using the “comparison/survival” module, and the results were visualized as KM curves. Then, the linear correlation between RBM10 expression and CNV was also analyzed by GSCA (http://bioinfo.life.hust.edu.cn/GSCA/#/mutation), and the results were visualized as a scatter diagram.

DNA methylation is among the major types of gene changes closely associated with tumor occurrence and development. The correlation between RBM10 DNA methylation level and its expression was assessed with the GSCA database. Furthermore, the RMB10's promoter methylation levels in various tumors and the related healthy tissues were obtained using UALCAN (http://ualcan.path.uab.edu/). The level of RBM10 methylation and prognostic value of patients with pan-cancer were also analyzed, which results were visualized as KM curves.

### 2.5. Analysis of Immune Infiltration

We employed xCell and TIMER2 for evaluating the correlation of the RBM10 expression with different levels of immune cell infiltration in tumors. We visualized the results as a heat map. The SangerBox (http://past20.sangerbox.com/Gene) website was used to study the relationship of the RBM10 expression with MSI, TMB, ESTIMATEScore, different types of immune cells, and ICP genes in various tumors from the TCGA cohort. The Spearman rank correlation test was performed. In addition, the TISIDB database (http://cis.hku.hk/TISIDB/) helped us get the heat maps of the coexpression between RBM10 and major histocompatibility complex (MHC) genes, immunosuppressive genes, chemokines, and chemokine receptors in different tumors.

### 2.6. Drug Sensitivity Analysis

The Genomics of Drug Sensitivity in Cancer (GDSC, https://www.cancerrxgene.org/) was utilized for analyzing the association of the mRNA expression of RBM10 with the drug IC_50_ in pan-cancer. In addition, the association of the RBM10 expression with various drug sensitivities was evaluated using the Cancer Therapeutics Response Portal (CTRP, http://portals.broadinstitute.org/ctrp/).

### 2.7. Gene Enrichment Analysis

We constructed the protein-protein interaction network (PPI) of RBM10 with the help of the STRING database (https://string-db.org/). The top 50 RBM10-related target genes of all TCGA tumors and healthy tissues were obtained based on the “Similar Genes Detection” module of GEPIA2. The top 10 target genes with the highest correlation were selected, and the heat maps of these 10 target genes were obtained by using the “Gene_Corr” module of TIMER2. In addition, GSEA helped in the analysis of the high and low expression groups of RBM10. The results showed the first three terms of the Kyoto Encyclopedia of Genes and Genomes (KEGG) pathway and HALLMARK analyses.

### 2.8. Cell Culture

Human LUAD cell lines H827 (Cat. No. CL-0094) were sourced from Procell Life Science and Technology Co., Ltd. (Wuhan, China). H3255 (ATCC, Cat. No. CRL-2882) cell lines were purchased from the American Type Culture Collection (ATCC, Manassas, VA, USA). All cell lines were stored in the central laboratory of the Cancer Hospital and Shenzhen Hospital, Chinese Academy of Medical Sciences (Shenzhen, China). All cell lines were authenticated by short tandem repeat (STR) profiling. All cells were both cultured in RPMI-1640 medium (Cat. No. C11875500BT, Gibco, Beijing, China) containing10% fetal bovine serum (FBS, Cat. No. ST30-3302, PAN, Germany) in a 5% CO_2_ humidified cell cultured incubator at 37°C.

### 2.9. Cell Transfection and Lentiviral Infection

Following the steps of Cao et al. [12], H3255 and H827 cells were transfected with siRBM10 (RiboBio, Guangzhou, China) or overexpression RBM10 plasmids (Han bio Biotechnology Company, Shanghai, China), and the transfection efficiencies were detected by western blotting. The specific siRNA sequences were as follows: siRBM10: GCATGACTATGACGACTCA and siNC: GCATGACTATGACGACTCA. In addition, we also transfected LUAD cell with RBM10 overexpression virus (Han bio Biotechnology Company, Shanghai, China) to construct H3255 cell line with the stable RBM10 overexpression.

### 2.10. Cell Counting Kit-8

After transfection of 36 h, the cell counting kit-8 (CCK-8, Cat. No. HY-K0301, MCE, Shanghai, China) was used to assess the cell viability, following the manufacturer's instructions for the entire operation. Briefly, LUAD cells (3 × 10^3^/100 *μ*l per well) were seeded in 96-well plates and cultured in a humidified incubator for various time points (1, 2, 3, 4, and 5 days). The 10 *μ*l CCK-8 solution was added to 100 *μ*l culture medium with 10% FBS and incubated for 1.5 h. The absorbance of each well was measured at 450 nm using a spectrophotometer (Infinite M200 PRO TECAN, China). All the experiments were carried out for 3 times. The following equation was applied for calculation: the cell viability (%) = (*A* experiment − *A* control)/(*A* blank − *A* control) × 100%.

### 2.11. Clone Formation Assay

In brief, the cells were seeded in six plates with 700 cells/well. The cells were cultured in an incubator continuously for 14 days. Fresh culture medium with 10% FBS was replaced every three days. The supernatant was discarded, washed with phosphate buffer saline, fixed with 4% paraformaldehyde, and stained with 1% crystal violet. ≥50 cells/colony were counted. Clone formation rate (%) = (number of clones/number of inoculated cells) × 100%. The experiment was conducted in triplicate.

### 2.12. Transwell Assays

Transwell assays were used to assess cell migration and invasion abilities. For migration assays, 3 × 10^4^ cells per well were resuspended in 300 *μ*l serum-free medium and seeded in the upper chamber (8.0 *μ*m pore size, Cat. No. 3422, Corning, USA). 700 *μ*l fresh medium containing 10% FBS was added to the lower chamber. The cells were incubated in an incubator with 5% CO_2_ at 37°C for 24 h. For invasion experiments, 30 *μ*l of Matrigel (Cat. No. 356234, Corning, USA) was evenly spread throughout the upper chamber and placed in the incubator for 2 h. 5 × 10^4^ cells were added to the upper chamber and cultured for 48 h. The noninvasive cells and Matrigel in the upper chamber were removed, and then, the cells were fixed with 4% paraformaldehyde for 15 min and stained with 0.1% crystal violet for 60 min. Cells were observed and counted in five random fields of each membrane selected under an inverted microscope (Leica Microsystems Inc., USA). All experiments were carried out in 3 times.

### 2.13. Western Blotting (WB)

The detail experiment procedures of WB were performed as described previously [18]. Cell precipitates or tumor tissues were retrieved and lysed on ice by using radioimmunoprecipitation assay (RIPA) lysis buffer (Cat. No. P0013B, Beyotime Biotechnology, Shanghai, China) containing protease inhibitors. Protein supernatants were collected, and then, the protein content was analyzed by enhanced BCA Protein Assay Kit (Cat. No. P0009, Beyotime Biotechnology, Shanghai, China). 60 *μ*g of protein per sample was separated on 10% sodium dodecyl sulfate-polyacrylamide gel electrophoresis (SDS-PAGE) and then transferred onto polyvinylidene fluoride (PVDF) membranes (Cat. No. IPVH00010, Millipore, USA). After blocking with 5% nonfat milk for 1 h at room temperature (RT), the membranes were incubated with primary antibodies overnight at 4°C. The next day, the membranes were incubated with the corresponding horseradish peroxidase- (HRP-) labeled secondary antibodies for another 1 h at RT. The proteins were visualized using electrochemiluminescence (ECL) western blotting substrate kit (Cat. No. PE0010, Solarbio, Beijing, China) and imaged using imaged with a chemiluminescence imaging system (TANON-5200MULTI). Relative protein level was standardized to the *β*-actin level. The primarily antibodies were used: PD-L1 (dilution, 1 : 2000, Cat. No. 66248-1-Ig, Proteintech, Wuhan, China), RBM10 (dilution, 1 : 1000, Cat. No. 14423-1-AP, Proteintech, Wuhan, China), and *β*-actin (dilution, 1 : 50000, Cat. No. 66009-1-Ig, Proteintech, Wuhan, China). The HRP-labeled secondary antibodies were as follows: peroxidase-conjugated goat anti-rabbit IgG (dilution, 1 : 10000, Cat. No. ZB-2301, Nakasugi Golden Bridge Biotechnology Co., Ltd., Beijing, China) and peroxidase-conjugated goat anti-mouse IgG (dilution, 1 : 10000, Cat. No. ZB-2305, Nakasugi Golden Bridge Biotechnology Co., Ltd., Beijing, China). Quantification of the protein was analyzed by using the ImageJ software (NIH, MA, USA). All experiments were carried out in triplicate.

### 2.14. PD-L1 Protein Half-Life Analysis

According to the manufacturer's instructions, LUAD cells were treated with 20 *μ*g/ml cycloheximide (CHX, Cat. No. HY-12320, MCE, China) for a specific time. Then, cells were collected and lysed for WB to detect the half-life of PD-L1 protein. The experiments were repeated three times.

### 2.15. In *Vivo* Experiment

Female BALB/c nude mice (4~5 weeks of age, 18-20 g) were purchased from Guangdong Yaokang Biotechnology Co., Ltd. (Guangdong, China). All experiments on mice were carried out according to the approval of the Animal Care and Ethics Committee of the Cancer Hospital and Shenzhen Hospital, Chinese Academy of Medical Sciences (Ethics: KYKT2021-13-1). The high concentration of Matrigel was purchased from Corning (Cat. No. 354248, Corning, USA). Briefly, approximately 5 × 10^6^ H3255 cells transduced with either vector or RBM10 were resuspended in 200 *μ*l PBS/Matrigel (3 : 1) and then were injected into the left (H3255-vector) and right (H3255-RBM10) armpits of mice (*n* = 5). Tumor size was measured using vernier caliper every 7 days. Tumor volume (mm^3^) = length × width^2^ × 0.5. Then, we plotted the tumor growth curve. After 35 days, all nude mice were sacrificed, and the subcutaneous tumors were resected and photographed. After that, a part of tumor tissues was stored immediately in liquid nitrogen and kept at -80°C. Total proteins were extracted for WB as previously described [18]. A part of the tumor tissues was soaked in formalin and embedded in paraffin for immunohistochemistry (IHC).

### 2.16. Immunohistochemistry (IHC) Staining

For IHC analysis, detailed experiment steps have been described previously [18]. In brief, all xenograft tumor tissues were first embedded in paraffin. Paraffin sections were dewaxed, dehydrated, and then subjected to antigen retrieval. Endogenous peroxidase activity was blocked with 3% hydrogen peroxide. The antigen repair was performed with citric acid or EDTA and then incubated with specific primary antibodies overnight at 4°C. The next day, slides were incubated with secondary antibody for 20 min at RT. The sections were full rinsed with tap water, counterstained, dehydrated, and sealed. Finally, we used a microscope (Leica Microsystems Inc., USA) to observe the tissue sections. The primary antibodies against Ki67 (dilution, 1 : 1000, Cat. No. GB111499, Servicebio, Wuhan, China), PD-L1 (dilution, 1 : 2000, Cat. No. 10366-1-AP, Proteintech, Wuhan, China), and RBM10 (dilution, 1 : 2000, Cat. No. GB113203, Servicebio, Wuhan, China) were used for IHC.

### 2.17. Statistical Analysis

The GraphPad Prism 6.0 software was used for statistical analysis, and all data were expressed as mean ± SD. Each experiment was done in triplicate. *p* < 0.05 was considered statistically significant.

## 3. Results

### 3.1. RBM10 Expression in Human Normal and Tumor Tissues

**Figure 1:**
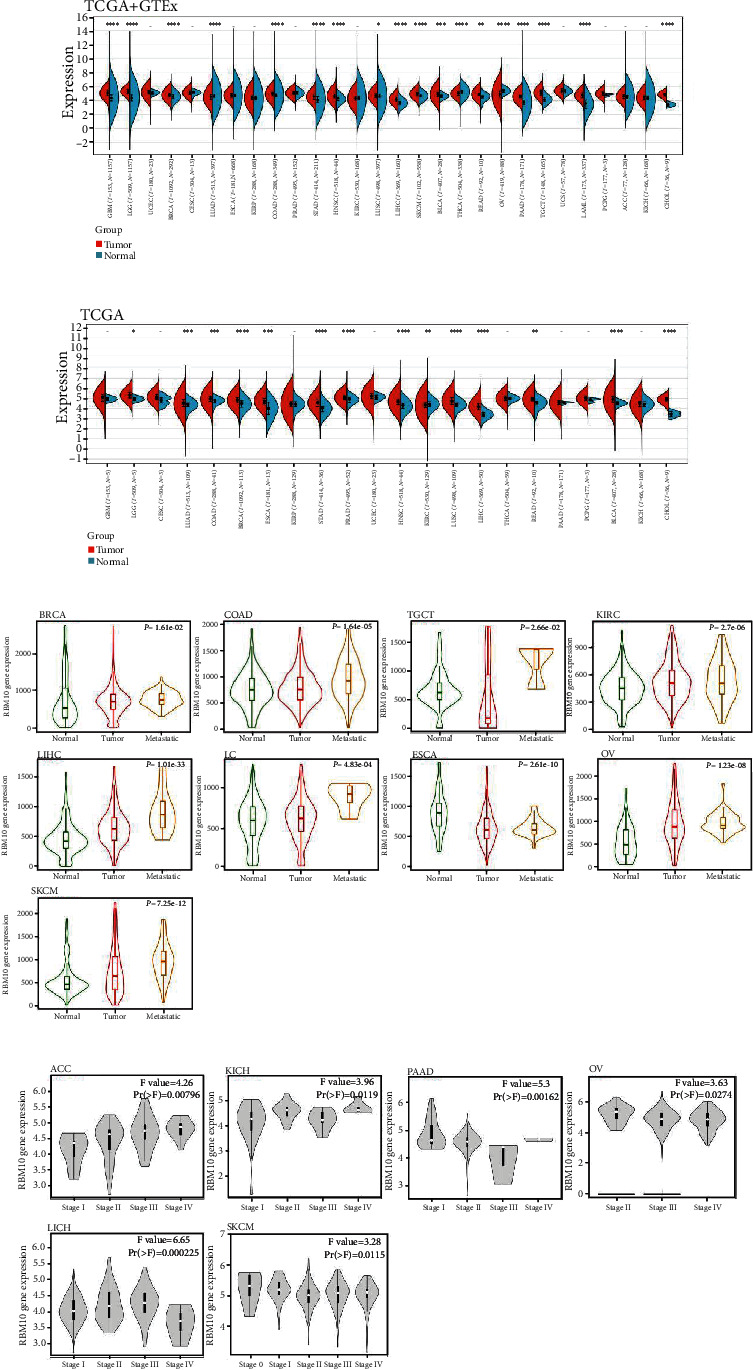
Pan-cancer analysis of the RBM10 expression. (a) RBM10 mRNA expression levels in different cancers and normal tissues of TCGA and GTEx databases. (b) RBM10 mRNA levels in tumor and normal tissues for 22 cancers of TCGA. (c) The expression levels of RBM10 mRNA in different primary tumors, metastatic tumors, and corresponding normal tissues were assessed according to TNM plotter website. (d) The correlation between RBM10 expression and the pathological stages of cancers using GEPIA. ^∗^*p* < 0.05, ^∗∗^*p* < 0.01, and ^∗∗∗^*p* < 0.001. -: not significant.

By integrating the TCGA and GTEx, RBM10 mRNA was expressed increasingly in most tumor tissues than in healthy tissues, including BRCA, LUSC, COAD, GBM, LGG, STAD, PAAD, LIHC, HNSC, TGCT, SKCM, BLCA, READ, LAML, and CHOL (Figure 1(a)). However, compared with adjacent normal tissues, mRNA levels of RBM10 were significantly lower in LUAD, THCA, and OV (Figure 1(a)). When only the TCGA database was included, RBM10 mRNA was greatly upregulated in LGG, LUAD, COAD, BRCA, ESCA, STAD, PRAD, HNSC, LUSC, LIHC, READ, BLCA, and CHOL but downregulated in KIRC (Figure 1(b)). In addition, in BRCA, COAD, TGCT, KIRC, LIHC, lung cancer, ESCA, OV, and SKCM, the expression of RBM10 in metastatic tumor tissues was markedly increased in comparison with that in the corresponding primary tumor tissues (Figure 1(c)).

Subsequently, we observed that RBM10 mRNA was the highest in the ovary, followed by the pituitary gland and cerebral cortex (NX > 35, Figure S1A), according to the HPA, GTEx, and FANTOM5 datasets. In most other normal tissues, the RBM10 mRNA expression was low, indicating its low mRNA tissue specificity. In addition, the analysis of the Cancer Cell Line Encyclopedia (CCLE) database indicated that RBM10 had the highest expression in small-cell lung cancer (SCLC) cells and the lowest in HNSC cell lineage (Figure S1B). The analysis of the CPTAC database showed that in comparison with the surrounding healthy tissues, RBM10 protein level was considerably increased in UCEC, breast cancer, colon cancer, KIRC, OV, HNSC, LUAD, GBM, and hepatocellular carcinoma (HCC) (Figure S2A).

Moreover, the RBM10 expression was strongly linked with the stages of tumors in ACC, KICH, LIHC, OV, PAAD, and SKCM (all *p* < 0.05, Figure 1(d)). Specifically, in ACC and KICH, the RBM10 level gradually increased with the increase of tumor grades. However, in OV, PAAD, and SKCM, the RBM10 expression decreased with increasing tumor grades. Interestingly, the RBM10 expression was low in stage IV, medium in stage I, and high in stages II and III in LIHC. Further, the RBM10 mRNA expression was considerably different from different molecular subtypes of BRCA, HNSC, KIRP, LGG, READ, OV, LUSC, and PRAD (Figure S3A). We did not observe a significantly different association between the expression level of RBM10 mRNA and the stages of the tumor or with the molecular subtypes in other cancer types (Figure S3B and S3C).

To further clarify the function of RBM10, a single-gene Gene Ontology (GO) analysis on RBM10 was explored by using SangerBox. The results showed that RBM10 was primarily involved in biological processes including negative regulation of mRNA splicing through spliceosomes and RNA splicing and positive regulation of smooth muscle cell apoptotic process. As a cellular constituent, RBM10 mRNA is present in the cell nuclear speck. In terms of molecular function, RBM10 is mainly involved in miRNA binding (Figure S2B). The details are obtained in Table S2.

### 3.2. Prognostic Value of RBM10

**Figure 2:**
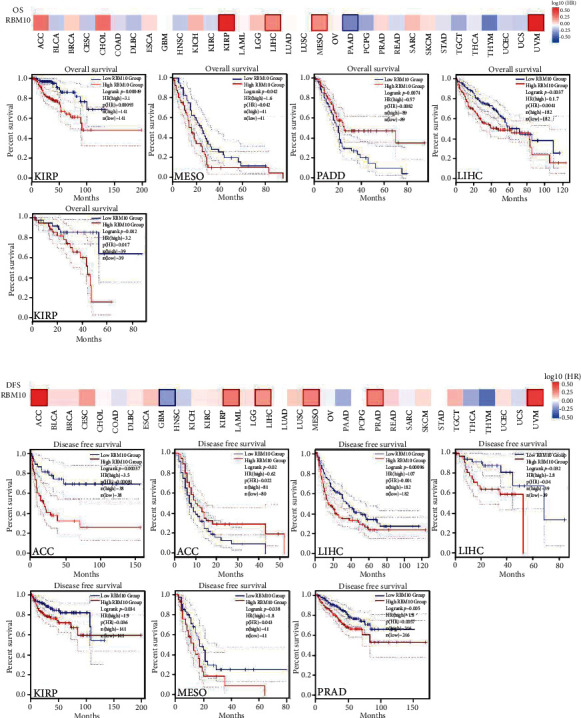
The prognosis value of RBM10 in human tumors. The median RBM10 expression was taken as the cut-off value. (a) The survival heat map represented the relationship between RBM10 expression and overall survive (OS) in TCGA tumors by using GEPIA2 tool. The survival curves showed the significant differences correlation of the RBM10 expression with OS of KIRP, MESO, PAAD, LIHC, and UVM. *p* < 0.05 was statistically significant. (b) The survival heat map represented the link of RBM10 level with disease-free survival (DFS) in TCGA tumor by GEPIA2 tool. The survival curves showed the significant difference correlation of the RBM10 expression with DFS of ACC, GBM, LIHC, UVM, KIRP, MESO, and PRAD. *p* < 0.05 was statistically significant.

The GEPIA2 was utilized for evaluating the prognostic value of RBM10 in TCGA pan-cancer. The median expression of RBM10 in different tumor types was taken as the cut-off value, and the tumors were categorized into high and low expression groups. As shown in Figure 2(a), high RBM10 level is a risk factor for overall survival (OS) in KIRP (hazard ratio, HR = 3.1, *p* = 0.00049), MESO (HR = 1.6, *p* = 0.042), LIHC (HR = 1.7, *p* = 0.0037), and UVM (HR = 3.2, *p* = 0.012). In contrast, the high RBM10 expression represented a better OS in PAAD patients (HR = 0.57, *p* = 0.0074). In addition, DFS results showed that in ACC (HR = 3.5, *p* = 0.00039), LIHC (HR = 1.7, *p* = 0.00096), UVM (HR = 2.8, *p* = 0.032), KIRP (HR = 1.9, *p* = 0.034), MESO (HR = 1.8, *p* = 0.038), and PRAD (HR = 1.8, *p* = 0.005), low RBM10 level was positively linked with better DFS but negatively linked with better DFS in GBM patients (HR = 0.62, *p* = 0.02, Figure 2(b)).

Further, the clinical prognosis of RBM10 in 21 types of tumors was examined through the Kaplan-Meier plotter website. The increased expression of RBM10 was closely associated with the poorer OS and RFS in KIRP and LIHC and the better OS and RFS in PAAD, STAD, and THCA (Figure S4A and S4B). Patients with high RBM10 level in BLCA, CESC, and PCPG had longer OS, while patients with ESCA, KIRC, and SARC had significantly shorter OS. Elevated RBM10 levels predicted better RFS in OV patients but worse in READ patients (Figure S4A and S4B). Interestingly, the high RBM10 expression was positively associated with better OS and poorer RFS in patients with UCEC (Figure S4A and S4B). In addition, univariate Cox regression analysis of the SangerBox database showed that the RBM10 expression was closely related to OS, DSS, DFI, and PFI in patients with different types of tumors (Figure S5). Based on the above information, it is observed that the expression of RBM10 is greatly linked with the prognosis of patients with various cancer types. Although the prognostic values of different malignancies are different, the correlation between RBM10 and clinical prognosis may provide new clues to the potential pathogenesis of different tumors.

### 3.3. Genetic Variation Analysis of RBM10

**Figure 3:**
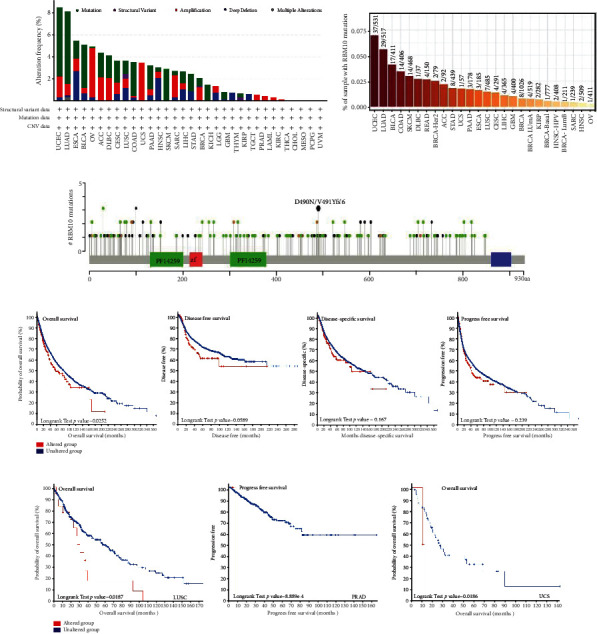
Mutation characteristics of RBM10. (a) Alteration frequency of RBM10 in different cancers was accessed by the cBioPortal website. (b) Mutation frequency of RBM10 from the TIMER2.0 database. (c) Different mutation sites of RBM10. (d) The relationship of RBM10 mutation status with OS, DFS, DSS, and PFS in all TCGA tumors. (e) The KM curve showed the association of RBM10 mutation status with OS of LUSC, PFS of PRAD, and OS of UCS, respectively. *p* < 0.05 was statistically significant.

Next, we assessed the genetic alternations of RBM10 in pan-cancer using cBioPortal tool. As shown in Figure 3(a), “mutation” was the most widely gene change in RBM10 in most tumors, and the mutation rates of LUAD, UCEC, and BLCA were the highest, 6.54%, 6.24%, and 3.89%, respectively. Additionally, “mutation” was the only RBM10 genetic change type in SKCM and KICH. And “amplification” was the only gene change type of UCS, PRAD, LAML, KIRC, and THCA. The TIMER2.0 database analysis showed that UCEC (37/531), LUAD (29/517), and BLCA (17/411) were the three malignancies showing the highest rate of mutations in the RBM10 gene (Figure 3(b)). In LUAD, BLCA, and PAAD, the RBM10 expression in its mutant type was considerably decreased in comparison with that in the RBM10 wild type (Figure S6).  Figure 3(c) shows the type and site of RBM10 gene mutation. Subsequently, KM curves were generated to find the prognostic value of RBM10 gene changes in TCGA pan-cancer datasets. Compared with the RBM10 unchanged groups, the OS (*p* = 0.0252) of the RBM10 changed groups was worse, while no major variation was observed in DFS (*p* = 0.0589), DSS (*p* = 0.167), and PFS (*p* = 0.239) (Figure 3(d)). Further, we also found that in LUSC (*p* < 0.001) and UCS (*p* = 0.007), OS of the RBM10 altered groups was substantially decreased in comparison with that of the corresponding RBM10 unaltered groups (Figure 3(e)). In PRAD (*p* < 0.001), the PFS of the RBM10 unaltered group was better, indicating that the change of RBM10 gene may be related to cancer progression (Figure 3(e)). In addition, the expression of RBM10 in ACC, ESCA, HNSC, LUAD, BLCA, CESC, LUSC, OV, STAD, and UCS was greatly positively associated with CNV but negatively linked with KIRP, LGG, and PRAD (Figure S7A and S7B). No significant association between RBM10 expression and CNA was found in other cancers (Figure S7C). More details were seen in Table S3.

### 3.4. DNA Methylation Analysis of RBM10

**Figure 4:**
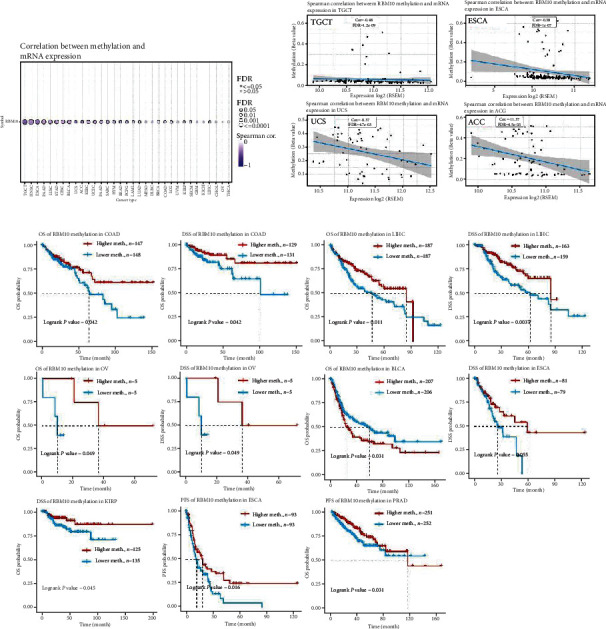
RBM10 methylation level in pan-cancer. (a) Spearman's correlation of RBM10 methylation with the mRNA expression in various cancers. Blue bubbles represent negative correlation. The darker the color, the higher the correlation. Bubble size was positively correlated with FDR. Black outline indicates FDR < 0.05. (b) Scatter plots represented the top four tumors with the strongest correlations. (c) Prognostic analysis of RBM10 between hypermethylated and hypomethylated groups in different types of cancer. *p* < 0.05 was statistically significant.

The change of DNA methylation is considered to be the major factor of tumorigenesis and progression [19]. The methylation levels of RBM10 in various types of tumor tissues were analyzed by the GSCA database. The results revealed that RBM10 mRNA was significantly negatively linked with methylation level in the majority of types of tumors, except MESO, DLBC, UVM, GBM, KICH, CHOL, OV, and THCA (Figure 4(a)).  Figure 4(b) indicates the four tumor types with the highest correlation, including TGCT, ESCA, UCS, and ACC. Furthermore, the methylation level of the RBM10 promoter in pan-cancer tissues was analyzed by the UALCAN tool. Compared with that in the surrounding healthy tissues, the RBM10 promoter methylation levels in BLCA, LIHC, and PRAD tumor tissues were considerably enhanced, while it was decreased only in BRCA and TGCT tumor tissues (Figure S8A). Then, we drew KM curves for describing the prognostic value of RBM10 promoter methylation in the TCGA pan-cancer datasets. The high RBM10 methylation level predicted a better prognosis of OS and DSS in COAD, LIHC, and OV patients (Figure 4(c)). In ESCA, low RBM10 methylation level was strongly positively associated with decreased DSS and PFS. In addition, the low methylation level of RBM10 represented DSS in KIRP patients, and PFS in PRAD patients was even worse. Conversely, low RBM10 methylation levels significantly prolonged OS in patients with BLCA. Otherwise, the SangerBox database discovered the close link of the RBM10 expression with DNA methyl-transferase (DNMT) genes (DNMT1: red, DNMT2: blue, DNMT3A: green, and DNMT3B: purple) (Figure S8B). In LGG, LIHC, BLCA, BRCA, and SKCM, the expression of RBM10 was substantially positively associated with four DNMT genes (Figure S8B).

### 3.5. Association of RBM10 Expression with Tumor Immune

**Figure 5:**
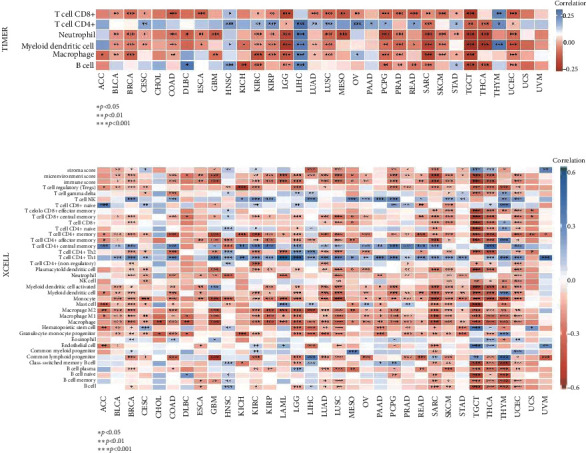
The association of the RBM10 expression with immune cell infiltration level in pan-cancer. (a) A heat map of the relationship between RBM10 expression and levels of immune cell infiltration in human cancer types was obtained using TIMER2. (b) A heat map of the correlation between RBM10 and levels of immune cell infiltration in 33 cancers was drawn using xCell. ^∗^*p* < 0.05, ^∗∗^*p* < 0.01, and ^∗∗∗^*p* < 0.001.

The association of the RBM10 expression with the level of immune cell infiltration was explored. TIMER2 suggested that the RBM10 expression was closely correlated with the infiltration levels of B cells in 14 types of cancer, CD8+ T cells in 22 types of cancer, CD4+ T cells in 18 types of cancer, neutrophils in 21 types of cancer, myeloid dendritic cells in 22 types of cancer, and macrophages in 19 types of cancer (Figure 5(a)). For most malignancies, the RBM10 expression was negatively associated with the infiltration levels of CD8+ T cells, B cells, neutrophils, macrophages, and myeloid dendritic cells but positively associated with CD4+ T cells. It should be noted that in LGG, SARC, TGCT, and UCEC, the RBM10 expression was negatively associated with all six immune cells, and it was considered positively associated with B cells, neutrophils, CD4+ T cells, and macrophages in LIHC. In addition, xCell data showed that RBM10 level was negatively associated with CD4+ T cell memory, monocyte, macrophage M1, macrophage M2, and macrophage in most tumors (Figure 5(b)). However, the RBM10 expression was positively associated with T cell CD4+ Th1 and T cell CD4+ central memory in many cancers, especially in LUAD and LUSC (Figure 5(b)). Interestingly, the RBM10 expression was negatively linked with the infiltration level of most immune cells in LGG, LUAD, LUSC, SARC, SKCM, TGCT, THCA, and UCEC (Figure 5(b)). TISIDB was used to analyze the RBM10 expression in various immune subtypes (C1 (wound healing) and C2 (IFN-*γ* dominant), C3 (inflammation), C4 (lymphocyte depletion), C5 (immune quiescence), and C6 (TGF-*β* dominance)). As shown in Figure S9A, RBM10 is significantly differentially expressed in various immune subtypes of KIRC, LIHC, LUAD, BRCA, COAT, LUSC, PCPG, SKCM, STAD, UCEC, PRAD, READ, SARC, and TGCT. The RBM10 expression was reduced in C6 subtype of 10 types of tumor excluding PRAD and TGCT but increased in C6 subtype of UCEC and TGCT. In addition, we also observed that the expression of RBM10 increased in C5 subtype of KIRC and decreased in C5 subtype of PCPG. RBM10 was not associated with different immune subtypes of other types of cancer (Figure S9B).

**Figure 6:**
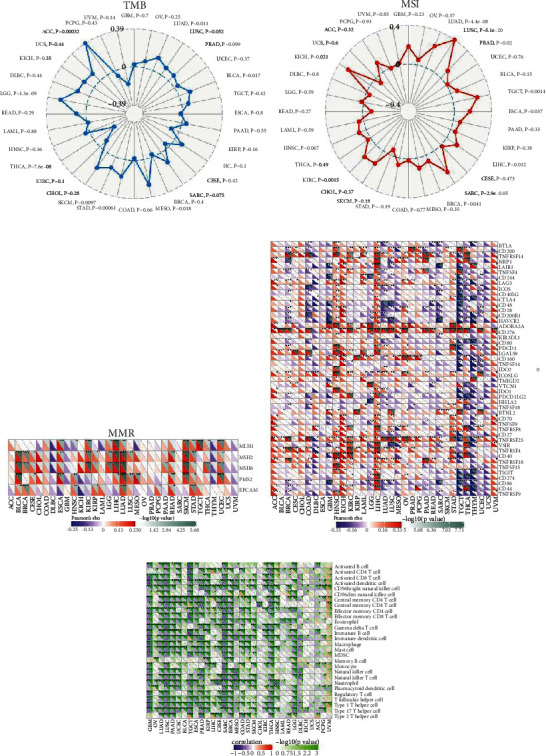
Relationship between RBM10 expression and tumor mutation burden (TMB), microsatellite instability (MSI), mismatch repairs (MMRs), neoantigens, immune checkpoint (ICP) genes, and some immune-related pathway. (a) Radar map of the correlation between RBM10 expression and TMB. (b) Radar map showed the association between RBM10 expression and MSI. (c) A heat map indicated the correlation of the RBM10 expression with the expression of MMR genes. The lower triangle of each square represented Spearman's correlation coefficient, and the upper triangle represented the *p* value. ^∗^*p* < 0.05, ^∗∗^*p* < 0.01, and ^∗∗∗^*p* < 0.001. (d) The heat map showed the association of RBM10 with ICP genes in pan-cancer using TCGA data from SangerBox. The upper triangle of each square represented the *p* value of correlation test, and the lower triangle represented Spearman's correlation coefficient. ^∗^*p* < 0.05, ^∗∗^*p* < 0.01, and ^∗∗∗^*p* < 0.001. (e) Heat map of the correlation between RBM10 expression and some immune-related pathway. The upper triangle of each square represented the *p* value of correlation test, and the lower triangle represented Spearman's correlation coefficient. ^∗^*p* < 0.05, ^∗∗^*p* < 0.01, and ^∗∗∗^*p* < 0.001.

Evidence shows that abnormal changes of tumor microenvironment (TME) affect a variety of biological behaviors of tumor cells, including promoting tumor cell proliferation, metastasis, and apoptosis inhibition [20]. We continued to evaluate the association of the RBM10 expression with TME using ESTIMATEScore, ImmuneScore, and StromalScore. The expression of RBM10 was negatively linked with ImmuneScore of 22 cancers, StromalScore of 17 cancers, and ESTIMATEScore of 22 cancers (Figure S10A–C) but positively linked with these three scores in UVM. We noticed that the top three tumors that most significantly correlated and associated between RBM10 expression and immune infiltration were GBM, LUSC and SARC (Figure S10D). Patients with high TMB/MSI-H have been shown to perform better in immunotherapy [19]. Subsequently, the association between TMB, MSI, and RBM10 expressions was examined.  Figure 6(a) indicates that RBM10 was positively correlated with the TMB of LUAD, BLCA, MESO, STAD, SKCM, LGG, and ACC but only negatively linked with the TMB of THCA. Similarly, we further discovered the RBM10 expression to be strongly positively associated with MSI of various malignancies, such as LUSC, PRAD, TGCT, LUAD, ESCA, LIHC, SARC, BRCA, KIRC, KICH, and GBM (Figure 6(b)). Notably, the RBM10 expression had the highest correlation with TMB of ACC (Spearman′s cor = 0.39) and MSI of LUSC (Spearman′s cor = 0.4). Further, we also found that the RBM10 expression was significantly linked with MMRs (MLH1, MSH2, MSH6, PMS2, and EPCAM) in most tumors, except in COAD, DLBC, GBM, ACC, CHOL, KICH, LAML, SARC, UCS OV, PAAD, and UVM (Figure 6(c)). Interestingly, the RBM10 expression was positively correlated with five MMR genes in LUAD and STAD and was positively correlated with MLH1, MSH2, MSH6, and PMS2 in ESCA, LIHC, SKCM, and UCEC (Figure 6(c)). In addition, in HNSC, the RBM10 expression was substantially negatively associated with MLH1. We also observed that RBM10 was closely related to the number of new antigens (Figure S11).

ICP genes are essentially involved in immune cell infiltration and immunotherapy [21]. We also analyzed the association of the RBM10 expression with 47 ICP genes.  Figure 6(d) illustrates that the RBM10 expression was substantially linked with 41 ICP genes in LIHC, 38 ICP genes in TGCT, and 37 ICP genes in THCA. RBM10 was positively associated with most ICP genes in HNSC, KICH, and LIHC, indicating the immunosuppressive role of RBM10 in these tumors, and targeting it can achieve better immunotherapeutic results. Conversely, RBM10 was substantially negatively associated with most ICP genes in COAD, TGCT, THCA, THYM, and UCEC, which suggested that targeting this gene in the stated tumors might not respond well to immunotherapy. Furthermore, the effect of the RBM10 expression on the response to ICB therapy was investigated using the Tumor Immune Dysfunction and Exclusion (TIDE) score. The higher the TIDE score, the worse the effect of immune checkpoint blockade (ICB), and the shorter the OS of patients receiving ICB [22]. We observed that in HCC, TGCT, CESC, THCA, COAT, KIRP, KIRC, PCPG, and UCEC, the TIDE score of the RBM10 high expression group was much higher in comparison with that of the RBM10 low expression group, indicating that high RBM10 level may promote immune escape and lead to adverse ICB response in these tumors (Figure S12).

Finally, we also observed that in most tumors, the expression of RBM10 was closely linked with different types of immune cells (such as activated dendritic cells, activated CD4/CD8 T cells, CD56 bright natural killer cells, effector memory CD4/CD8 T cells, eosinophils, immature B cells, immature dendritic cells, macrophages, mast cells, neutrophils, T follicular helper cells, type 1 T helper cells, and type 17 T helper cells), except CHOL, LAML, KICH, and UCS (Figure 6(e)). Moreover, RBM10 level was substantially negatively associated with the MHC genes, chemokines, chemokine receptors, immune-stimulatory factors, and immunosuppressive factors in most tumor types (Figure S13A–E). In addition, among these immunosuppressive markers, PD-L1, IL10, CSF-1R, and TGFB1 were considerably negatively associated with the expression of RBM10 in most tumors, especially in LUAD (Figure S13F**)**. Overall, RBM10 may be a good immunotherapy target in general and a predictor of response to immunotherapy.

### 3.6. Relationship between RBM10 and Drug Sensitivity

The GDSC database was utilized for analyzing the association of the RBM10 expression with 251 drug sensitivities. The results suggested that there was a major link between RBM10 expression and 133/251 drug sensitivities (Table S4 and S5). Specifically, the expression of RBM10 was positively associated with 42/133 drugs, among which it was substantially positively associated with trametinib, 17-AAG, PD-0325901, RDEA119, cetuximab, afatinib, and XAV939 (all cor > 0.25), indicating that the high expression of RBM10 may lead to drug resistance (Table S4). The RBM10 expression was negatively associated with the IC_50_ of 91/133 drugs, among which GSK1070916 (cor = −0.344712733), NPK76-II-72-1 (cor = −0.337091794), navitoclax (cor = −0.336103607), BX-912 (cor = −0.331763642), and I-BET-762 (cor = −0.303893649) showed the strongest negative correlation with the expression of RBM10 (Table S5). In addition, we observed a considerable association of multiple drug sensitivities with the RBM10 mRNA expression through the CTRP database, and the results were demonstrated in supplementary Table S6.

### 3.7. Functional Enrichment Analysis of RBM10

**Figure 7:**
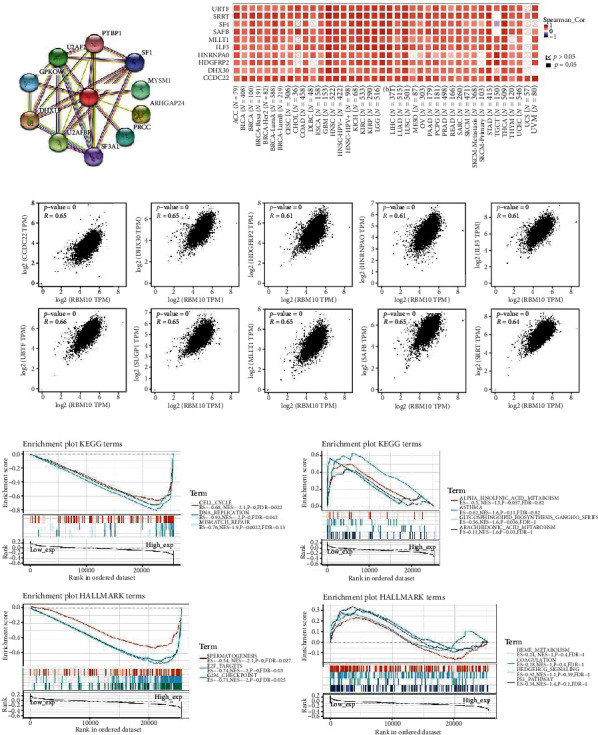
Enrichment analysis of RBM10. (a) The RBM10 interacting proteins were obtained using STRING tool. (b) Top 50 RBM10-related genes were explored through GEPIA2, and we selected top 10 genes (CCDC22, DHX30, HDGFRP2, HNRNPA0, ILF3, MLLTT1, SAFB, SF4, SRRT, and UBTF). A heat map indicated the correlation of the RBM10 expression with the expression of selected target genes in pan-cancer by using TIMER2.0. (c) The correlation between RBM10 expression and the selected 10 target genes using GEPIA2. (d) Enrichment analysis of RBM10 in KEGG and HALLMARK pathways.

Using the STRING database, the RBM10 expression was closely associated with PTBP1, SF1, MYSM1, ARHGAP24, PRCC, SF3A1, U2AFBP, GPKOW, and U2AF2 proteins (Figure 7(a)). Then, the top 50 genes significantly related to the RBM10 expression in the TCGA pan-cancer dataset were obtained using GEPIA2 (Table S7). The top 10 genes with the highest correlation were selected, namely, UBTF, SAFB, DHX30, HNRNPA0, SUGP1 (SF4), MLLT1, ILF3, HDGFRP2, SRRT, and CCDC22 (Table S7 and Figure 7(b)). Interestingly, RBM10 was substantially positively associated with these 10 genes in most types of cancer but was substantially negatively associated with SUGP1 (SF4) only in TGCT (Figure 7(b)).  Figure 7(c) shows the correlation between the expression of these 10 genes and RBM10 expression in pan-cancer by GEPIA2. As shown in Figure 7(d), KEGG enrichment showed that the high expression of RBM10 was mainly related to CELL_CYCLE, DNA_REPLICATION, and MISMATCH_REPAIR, and the low expression of RBM10 was closely associated with ALPHA_LINOLENIC_ACID_METABOLISM, ASTHMA, GLYCOSPHINGOLIPID_BIOSYNTHESIS_GANGLIO_SERIES, and ARACHIDONIC_ACID_METABOLISM (Table S8). HALLMARK term indicated that the high expression of RBM10 is considerably negatively associated with SPERMATOGENESIS, E2F_TARGETS, and G2M_CHECKPOINT, but the downregulation of RBM10 is significantly positively correlated with HEME_METABOLISM, COAGULATION, HEDGEHOG_SIGNALING, and P53_PATHWAY (Figure 7(d) and Table S8).

### 3.8. RBM10 Inhibits the Proliferation, Migration, and Invasion of LUAD Cells and Affects the Protein Stability of PD-L1 In Vitro

**Figure 8:**
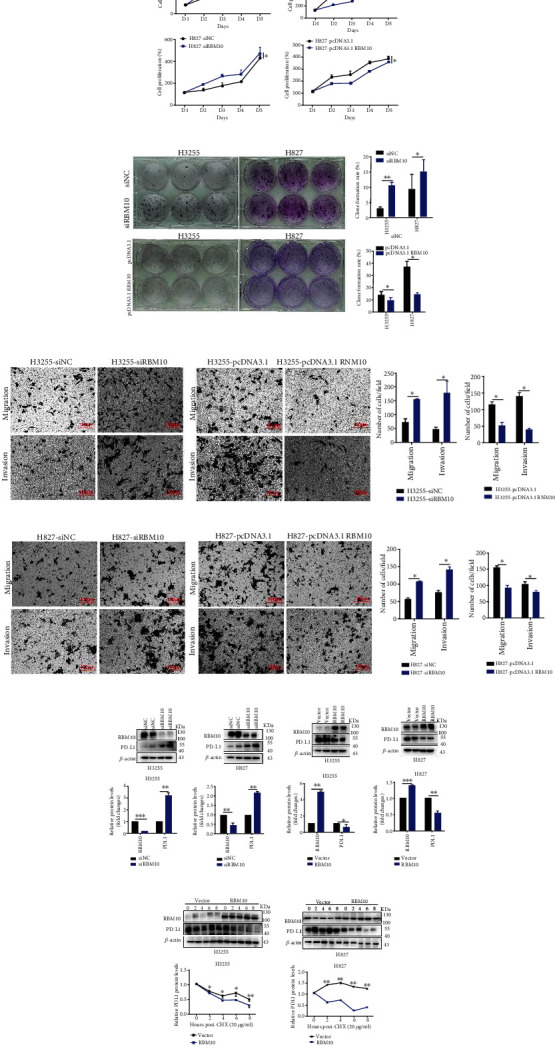
RBM10 inhibited the proliferation, migration, and invasion of LUAD cells and affects the protein stability of PD-L1 in *vitro*. After effectively upregulating or silencing the RBM10 expression in H3255 and H827 cells, (a, b) CCK-8 assays were performed to assess proliferation of LUAD cells. (c) The colony formation assays were used to measure cell clonalities. (d, e) Transwell assays were used to examine cell invasiveness and migration capability; scale bar, 100 *μ*m. (f, g) After silencing (f) or overexpressing (g) RBM10 in LUAD cells, western blot examined the protein level of PD-L1. (h, i) After cycloheximide (CHX, 20 *μ*g/ml) treated H3255 (h) and H827 (i) cells with stably overexpressing RBM10 for 0, 2, 4, 6, and 8 hours, western blot analyzed the protein stability of PD-L1. ^∗^*p* < 0.05, ^∗∗^*p* < 0.01, and ^∗∗∗^*p* < 0.001.

**Figure 9:**
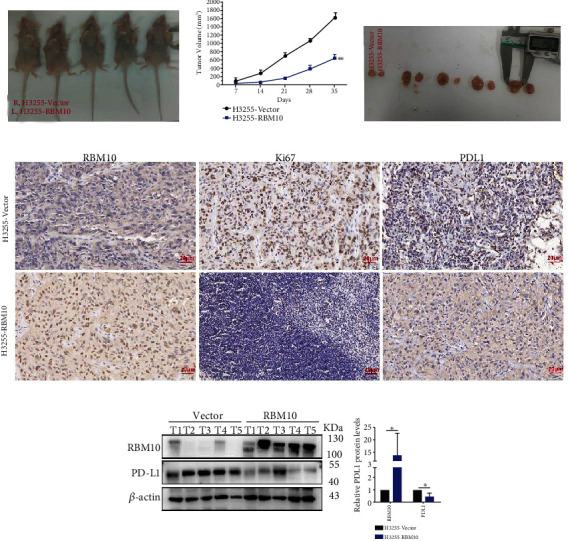
Overexpression of RBM10 inhibited LUAD tumor growth in *vivo*. (a) The model of subcutaneous transplanted tumor was established by subcutaneous injection of stable RBM10 overexpression groups (H3255-RBM10) and control groups (H3255-vector) into axilla of nude mice. (b) The xenograft tumor growth curves of the H3255-RBM10 and H3255-vector groups. (c) After the mice were sacrificed on day 35, we presented the representative images of the subcutaneous xenograft tumor lumps from the H3255-vector and H3255-RBM10 groups. (d) Representative images of IHC of Ki67, PD-L1, and RBM10 in nude mice xenograft tumor sections were shown (magnification, ×400; scale bar, 20 *μ*m). (e) The protein levels of PD-L1 and RBM10 in xenograft tumor tissues were detected by western blot. *β*-Actin was used as an internal control. ^∗^*p* < 0.05, ^∗∗^*p* < 0.01, and ^∗∗∗^*p* < 0.001.

After effectively upregulating or silencing the RBM10 expression, we found that downregulation of RBM10 promoted the proliferation, migration, and invasion of H3255 and H827 cells by using CCK-8, clone formation, and Transwell assays, while upregulation of RBM10 significantly decreased these functions in LUAD cell lines (Figures 8(a)–8(e)). In the subcutaneous xenograft model, we observed that the tumor size in the H3255 RBM10-overexpression xenograft groups was significantly decreased compared with the control groups at the end of the experiment (Figures 9(a) and 9(c)). The tumor growth curve exhibited the same trend (Figure 9(b)). Furthermore, the results of IHC showed that the overexpression of RBM10 significantly decreased the protein expression levels of Ki67 compared with the control group (Figure 9(d)). Taken together, these data imply that the overexpression of RBM10 decreases LUAD progression both in *vivo* and in *vitro*.

Additionally, RBM10 silencing significantly upregulated PD-L1 protein level in LUAD cells, while RBM10 overexpressing significantly decreased PD-L1 protein level (Figures 8(f) and 8(g)). Similarly, PD-L1 protein level was lower in tumor tissues generated by RBM10 overexpression cells than vector control based on IHC and WB (Figures 9(d) and 9(e)). After cycloheximide (CHX, 20 *μ*g/ml) treated LUAD cells for a specific time, we observed that upregulation of RBM10 significantly reduced the protein stability of PD-L1 in H3255 and H827 cells, and downregulation of RBM10 increased the protein stability of PD-L1 (Figures 8(h) and 8(i)). In all, these outcomes preliminary suggest that RBM10 may regulate the expression of PDL1 by affecting its protein stability.

## 4. Discussion

Cancer is the leading killer which seriously threats the human health. RBM10 is a RNA binding protein that mainly participates the splicing of precursor mRNA and posttranscriptional regulation. Most studies have proved that RBM10 may play a role in tumor progression. However, the role of RBM10 has not been widely studied in pan-cancer, and its function and potential mechanism in tumors are still unclear. To the best of our knowledge, this is the first systematic and comprehensive study on the expression level, prognostic value, genetic variation, DNA methylation, and the correlation with tumor immune cell infiltration of RBM10 in a pan-cancer dataset.

In our investigation, we first evaluated the RBM10 expression level in normal as well as tumor tissues of 33 cancers. The findings revealed that RBM10 was expressed abnormally in most cancers and highly in LGG, LUSC, BRCA, BLCA, CHOL, COAT, LIHC, HNSC, STAD, and READ. Interestingly, the RBM10 expression was upregulated in the TCGA-LUAD dataset, while the RBM10 expression was significantly reduced in the LUAD integrating the TCGA and GTEx datasets. The role of RBM10 in the progression of LUAD has been controversial. Jung et al. [10] reported that RBM10 inhibits the proliferation of LUAD cells by regulating RAP1/AKT/CREB signal pathway. Zhang et al. [23] confirmed that RBM10 inhibits the progression of LUAD by regulating the selective splicing of EIF4H. Our research group previously proved that RBM10 partially inactivated Wnt/*β*-catenin signaling pathway and inhibited proliferation and metastasis of LUAD cells [12]. These pieces of evidence suggest that RBM10 mainly acts as a tumor suppressor in LUAD. However, Sun et al. [13] found that as an oncogene, RBM10 promotes the progression of LUAD by promoting cell proliferation and inhibiting apoptosis. This inconsistency may be due to the tumor heterogeneity of tumor-related genes and different data collection sources [24]. At the same time, we also found that the expression of RBM10 was different in different tumor stages and molecular subtypes of various tumor types, indicating that RBM10 was closely related to tumor progression and tumor molecular subtypes. Previously, the correlation of the RBM10 expression with tumor stages was reported to be negative in pancreatic cancer [25]. The correlation of high expression of RBM10 in HCC with more advanced tumor stages was significantly positive [8]. Additionally, our prognostic analysis indicated that in certain malignancies, the high RBM10 expression was a risk factor and was considerably linked to the poorer OS, PFS, and RFS. However, in STAS, PAAD, and THCA, high RBM10 level predicted a better prognosis, which was consistent with previous confirmation of RBM10 might act as a protective factor in certain cancer types. In conclusion, although RBM10 has different or even opposite prognostic value in different tumors, all these data strongly lead to the suggestion that it can be used as a biomarker for prognostic prediction of tumor patients.

Tumor suppressor gene mutations or abnormal activation of oncogenes leads to tumor progression. In recent years, more and more scholars have devoted themselves to studying the relationship between gene mutation and human cancer progression and metastasis [26, 27]. RBM10 mutation is considered to be one of the most common mutation genes in solid tumors, including LUAD [23], colorectal cancer [28], bladder cancer [29], and pancreatic ductal adenocarcinoma [30]. It has been proved that RBM10 is frequently mutated in LUAD and often leads to decreased or lost function [23], and LUAD patients with RBM10 mutation have a shorter OS and higher disease recurrence rate [23]. In our research, RBM10 gene alternations were mainly “mutations” in most cancers, and the mutation frequency was the highest in LUAD, UCEC, and BLCA, which was consistent with the previous results [23, 29, 30]. Survival analysis demonstrated that the change of RBM10 substantially reduced the OS of LUSC, PRAD, and UCS. However, the potential molecular mechanism of RBM10 mutation affecting the clinical prognosis of tumor patients has not been clarified yet, and further exploration is needed in the future.

Abnormal DNA methylation is highly associated with the incidence and growth of tumors [31]. Previous studies have shown that RBM10 is related to DNA methylation [32, 33]. Here, for the first time, we found a negative correlation between RBM10 mRNA level and DNA methylation level based on pan-cancer, indicating a potential regulatory role of DNA methylation in RBM10. Interestingly, RBM10 methylation plays a role in promoting or inhibiting cancer in different cancers. Specifically, in COAD, LIHC, OV, ESCA, KIRP, and PRAD, hypomethylation of RBM10 was significantly positively correlated with poor prognosis. However, RBM10 hypomethylation significantly prolonged the OS of patients with BLCA. Unfortunately, there is no relevant study on the molecular mechanism of RBM10 methylation affecting tumor prognosis. Therefore, further research exploring the role and potential molecular mechanism of RBM10 methylation in different tumor progressions is necessary.

So far, little is known about the correlation between RBM10 expression and tumor immune infiltration. Pozzi et al. [34] found that the dengue virus reduced the splicing and innate immune response to host cells by targeting RBM10. Atsumi et al. [35] found that RBM10 regulated the activity of NF-*κ*B promoters through selective splicing of DNMT3B, thus regulating the development of inflammation. A bioinformatics study found that in HCC, the RBM10 expression was positively correlated with infiltration of CD8+ T cell, as well as the expression of PD-1 and PD-L1 [8]. However, a recent report showed that CD8+ T cells showed a higher level of infiltration in LUAD with RBM10 deletion [16]. In this study, we found that the RBM10 expression was negatively correlated with B cells, CD8+ T cells, neutrophils, macrophages, and DC cell infiltration levels in most tumor types, indicating that RBM10 was likely to affect tumor development and prognosis by impacting the tumor microenvironment. It is worth noting that in HCC, RBM10 was significantly positively correlated with a variety of immune cells, which was consistent with the results of Pang et al. [8]. In addition, we further found that the RBM10 expression was closely related to tumor-associated macrophages (TAMs) in a variety of cancers through xCell. TAM is the most prevalent group of tumor-infiltrating immune cells in the tumor microenvironment (TME) [36], which promotes tumor development through inhibition of immune clearance, promotion of tumor cell proliferation, and stimulation of angiogenesis [37], and it is linked to the poor clinical prognosis of tumor patients [38]. According to the activation status and different functions, TAM is mainly split into two cell subsets (proinflammatory M1 and anti-inflammatory M2 macrophages) [36]. Type M1 macrophages are typically activated by interferon-*γ* and lipopolysaccharide (LPS), which primarily secretes proinflammatory factors, and are crucial in the initial stage of inflammation. On the other hand, M2 macrophages are activated by Th2 cytokines which include IL-13, IL-4, and immune complexes, which mainly express anti-inflammatory factors as well as function in inflammatory response inhibition and promoting tissue repair. In addition, it has been demonstrated that tumor-infiltrating M2 macrophages are substantially linked to poor clinical outcomes in a variety of malignancies [39]. According to this study's findings, the correlation between RBM10 and the infiltration level of macrophages (M1 and M2 phenotypes) was significantly negative in most tumors, indicating that RBM10 may directly or indirectly affect macrophage polarization. It has been known that CD4+ T cell subsets play an important role in cancer immunity, including Th1, Th2, and regulatory T (Treg) cells. Importantly, the balance between Th1 and Th2 differentiation is an important factor in maintaining immune homeostasis, and disruption of Th1/Th2 balance and shift toward Th2 cells is significantly associated with immune escape and promotion of cancer progression [40]. In here, we also found that in most tumor types, the RBM10 expression was positively correlated with the T cell CD4+ Th1 cell infiltration level and negatively correlated with the T cell CD4+ Th2 cell infiltration level. In addition, the RBM10 expression was significantly negatively correlated with IL-10 and TGF-*β*1 in most tumors based on TISIDB. M2 subtype could facilitate survival and migration of tumor cell through expressing a variety of cytokines and growth factors, including TGF-*β*1 [41]. Taken together, based on these data, we speculate that the high RBM10 expression inhibited tumor progression via blocking immune escape by reducing M2 macrophages in some tumors. However, the specific mechanism of RBM10 affecting M2 macrophages needs to be investigated in the further research. In addition, it is well known that M2 macrophages mainly include M2a, M2b, and M2c. Therefore, we also need to further explore whether and how RBM10 affect the subtype of M2 macrophages. In general, these data strongly prove that RBM10 is highly associated with tumor immune infiltration and may inhibit or promote cancer progression by recruiting and regulating infiltrating immune cells.

It is reported that TME can not only affect the therapeutic effect of ICB but also induce drug resistance [42]. Previous research has demonstrated that cancers with high MSI (MSI-H), TMB, or neoantigens respond to immunotherapy more favorably [43]. In here, the RBM10 expression was found to be negatively correlated with StromalScore, ImmuneScore, and ESTIMATEScore in a variety of cancers, especially in GBM, LUSC, and SARC. And the RBM10 expression is closely related to TMB, MSI, the number of new antigens, and MMR genes in a variety of cancers. Immune checkpoint inhibitors are considered to be the most effective anti-tumor immunotherapy [44]. Here, the RBM10 expression is significantly associated with most ICP genes, especially in HNSC, KICH, LIHC, COAD, TGCT, THCA, THYM, and UCEC. Interestingly, we also found that the ICP genes significantly associated with RBM10 expression were different in different cancers. More importantly, RBM10 was considerably linked to genes encoding MHC, immune activation, immunosuppression, chemokine, and chemokine receptor proteins. Taken together, the above results display that RBM10 regulates tumor immunity in some cancers. Therefore, targeting RBM10 may be a promising immunotherapy strategy for specific cancer. As far as we know, no small molecule drug specifically targeting RBM10 has been developed. Therefore, new drugs targeting RBM10 in tumor-infiltrating immune cells can be developed in the future, which will bring good news to the immunotherapy of tumor patients.

Because of our interest in LUAD, we focused on investigating the potential biological role of RBM10 in LUAD. Through a series of in vitro cytological assays including CCK-8, clone formation, and Transwell assays, we confirmed that RBM10 acted as a tumor suppressor gene and inhibited the growth, migration, and invasiveness of LUAD cells. Consistently, the xenograft tumor model was also proved that upregulation of RBM10 significantly decreased tumor growth. The results of IHC showed that Ki67 level was lower in the H3255-RBM10 groups than in vector control. Immune checkpoint inhibitors (ICIs) have made remarkable clinical progress in the treatment of lung cancer. However, there are still many problems and challenges in the clinical application of ICI, such as limited benefit population, lack of effective biomarkers, drug resistance, and lack of precise combination therapy. In here, we especially evaluated the association of the RBM10 expression in LUAD with the expression of some immunosuppressive genes. We found the correlation of the RBM10 expression with PD-L1 (CD274), CSF1R, IL-10, and TGFB1 to be significantly negative based on the LUAD-TCGA dataset, indicating that RBM10 is a promising therapeutic target for LUAD. Subsequently, we observed that the overexpression of RBM10 decreased the protein level of PD-L1, and silencing RBM10 significantly increased PD-L1 protein level. We also observed consistent results in tumor xenografts. Furthermore, RBM10 overexpressing markedly reduced PD-L1 protein stability in LUAD cells. Therefore, these pieces of evidence strongly suggest that RBM10 may inhibit the progression of lung adenocarcinoma by regulating the expression of PD-L1. In the future, more studies are needed to clarify the molecular mechanism of RBM10 regulating PD-L1.

We also observed that high RBM10 level could lead to the drug resistance of trametinib, 17-AAG, PD-0325901, RDEA119, cetuximab, and afatinib. In contrast, the RBM10 expression was negatively correlated with GSK1070916, NPK76-II-72-1, navitoclax, BX-912, and I-BET-762. These data would provide a theoretical basis for clinical patients to choose antitumor drugs.

Our research was still insufficient. First of all, the data used in this study were from public data platforms and lack of clinical experimental verification. Secondly, bioinformatics analysis was used to prove that RBM10 was highly associated with tumor immune cell infiltration. In the future, a large number of cytological experiments and preclinical studies are needed to verify the impact of RBM10 on the human immune microenvironment and immunotherapy. Thirdly, we preliminarily confirmed that RBM10 downregulates the expression of PD-L1 by influencing the stability of PD-L1 protein, while the mechanism needs to be further elucidated in the future. Finally, this study confirmed the inhibitory effect of RBM10 on cell proliferation and metastasis of lung adenocarcinoma. Due to the heterogeneity of gene expression and function, the specific role and molecular mechanisms of RBM10 in various human cancers require further exploration.

## 5. Conclusions

In summary, this is the first comprehensive pan-cancer research for RBM10. The abnormal expression of RBM10 was closely linked to the prognosis value, genetic mutation, tumor immune microenvironment, immune regulation, TMB, and MSI of various human different cancers, which strongly indicate that RBM10 could be served as a potential biomarker for cancer prognosis and immunotherapy response. In addition, we also believe that these findings can provide suggestions for further exploring the role and molecular mechanism of RBM10 in different cancers in the future, and we can better understand the role of RBM10 in tumorigenesis.

## Data Availability

This study involves online databases which were included in Materials and Methods of the article. For more reasonable requests, please contact the corresponding author directly.

## Supplementary Materials

Supplementary Materials Figure S1: RBM10 mRNA expression in human normal tissues and different cancer cells. (A) RBM10 mRNA expression in various normal tissues was analyzed using the HPA/GTEx/FANTOM5 database. (B) The mRNA expression level of RBM10 in various cancer cells was analyzed from the data of the CCLE database. Figure S2: the protein expression of RBM10 in pan-cancer and a single-gene GO analysis of RBM10. (A) RBM10 protein level in different tumors and normal adjacent tissues through the CPTAC database. (B) SangerBox showed the positively association between RBM10 expression and each analysis result. Figure S3: the correlation between RBM10 and different molecular subtypes in pan-cancer. (A) The expression of RBM10 was significantly associated with molecular subtypes in BRCA, HNSC, KIRP, LGG, READ, OV, LUSC, and PRAD. (B) The RBM10 expression was not associated with molecular subtypes in ACC, COAD, ESCA, GBM, LIHC, PCPG, and SKCM. (C) The expression level of RBM10 was not associated with tumor stages in BLCA, BRCA, CESC, CHOL, COAD, DLBC, ESCA, HNSC, KIRC, KIRP, LUAD, LUSC, READ, STAD, TGCT, THCA, and UCEC. Figure S4: the prognosis analysis of RBM10 in tumors. (A, B) The correlation analysis of RBM10 expression with overall survival (OS, (A)) and relapse-free survival (RFS, (B)) in patients with different cancer types from the TCGA database of the Kaplan-Meier plotter. The median RBM10 expression was used to classify patients into high expression and low expression groups. *p* < 0.05 was statistically significant. Figure S5: (A–D) forest map showed the univariate Cox regression results of RBM10 for OS (A), DSS (B), DFI (C), and PFI (D) in multiple cancers. Figure S6: the expression level of RBM10 in the patients with RBM10 wild type (RBM10 WT) or RBM10 mutation (RBM10 Mut) of LUAD, BLCA, and PAAD. Figure S7: the correlation of RBM10 expression with CNV. (A) The association between RBM10 mRNA expression and CNV in different cancers using GSCA. Blue bubbles represented negative correlation, and red bubbles represented positive correlation; and the darker the color, the higher the correlation. Bubble size was positively correlated with FDR. Black outline indicates FDR < 0.05. (B) A significant difference correlation between RBM10 mRNA levels and CNV in ACC, BLCA, CESC, ESCA, HNSC, KIRP, LGG, LUAD, LUSC, OV, PRAD, STAD, and UCS. (C) There was no correlation between RBM10 mRNA expression and CNV among other human tumors. Figure S8: the promotor methylation level of RBM10 in various cancers. (A) The promoter methylation level of RBM10 in BLCA, BRCA, LIHC, TGCT, and PRAD. (B) The correlation between RBM10 expression and the level of DNMT genes in across different cancer types was investigated via SangerBox database. The red color: DNMT1; the blue color: DNMT2; the green color: DNMT3A; the purple: DNMT3B. Figure S9: relationship between RBM10 expression and different immune subtypes in human tumor. (A) The violin diagram showed that RBM10 is significantly associated with different immune subtypes in BRCA, COAD, KIRC, LIHC, LUAD, LUSC, PCPG, PRAD, READ, SARC, UCEC, TGCT, SKCM, and STAD using TISIDB. (B) There was no significant difference between RBM10 expression and immune subtypes in ACC, BLCA, CESC, CHOL, ESCA, GBM, HNSC, KICH, KIRP, LGG, MESO, OV, PAAD, THCA, UCS and UVM. Figure S10: the RBM10 expression was related to TME. (A) The correlation of the RBM10 expression with ImmuneScore in pan-cancer through SangerBox. (B) The association between RBM10 level and StromalScore in different cancer types based on the SangerBox website. (C) The relationship of the RBM10 expression with ESTIMATEScore across multiple cancer types using SangerBox. (D) The top three tumors that were most significantly related. Figure S11: the relationship between RBM10 expression and neoantigens. Figure S12: TIDE score of the high and low RBM10 expression groups in GBM, TGCT, CESC, THCA, COAD, KIRP, KIRC, PCPG, and UCEC. A low score indicated good efficacy. *p* < 0.05 was considered statistically significant. G1, RBM10 high expression: red color; G2, RBM10 low expression: blue color. Figure S13: the correlation of the RBM10 expression with immunorelation-related genes. (A–E) Heat maps indicated the association of the RBM10 expression with MHC (A), chemokines (B), chemokines receptors (C), immunostimulants (D), and immunoinhibitors (E). (F) Scatter plots showed that RBM10 was negatively correlated with PD-L1 (CD274), CSF1R, IL-10, and TGFB1 in LUAD. Table S1: list abbreviations. Table S2: a single-gene GO analysis of RBM10 by using the SangerBox database. Table S3: the correlation between RBM10 level and CNV via GSCA database. Table S4: the positive correlation of RBM10 mRNA level with the drug sensitivity in the GDSC database. Table S5: the negative correlation of RBM10 mRNA level with the drug sensitivity in the GDSC database. Table S6: a significant difference about the relationship of RBM10 level with drug sensitivity in CTRP was observed. Table S7: top 50 RBM10 related genes were explored using GEPIA2. Table S8: the detail enrichment results of KEGG pathway and HALLMARK of RBM10 in GSEA. Supplementary original western blots. (F) Original western blots of (Figure 8(f)). (G) Original western blots of (Figure 8(g)). (H) Original western blots of (Figure 8(h)). (I) Original western blots of (Figure 8(i)). (E) Original western blots of (Figure 9(e)).
