# Supplementary material for: RBM10 Is a Biomarker Associated with Pan-Cancer Prognosis and Immune Infiltration: System Analysis Combined with In Vitro and Vivo Experiments
Source: Oxid Med Cell Longev. 2022 Nov 28;2022:7654937. doi: 10.1155/2022/7654937 (PMC11401663; doi:10.1155/2022/7654937)
Supplement: Supplementary Materials — Figure S1: RBM10 mRNA expression in human normal tissues and different cancer cells. (A) RBM10 mRNA expression in various normal tissues was analyzed using the HPA/GTEx/FANTOM5 database. (B) The mRNA expression level of RBM10 in various cancer cells was analyzed from the data of the CCLE database. Figure S2: the protein expression of RBM10 in pan-cancer and a single-gene GO analysis of RBM10. (A) RBM10 protein level in different tumors and normal adjacent tissues through the CPTAC database. (B) SangerBox showed the positively association between RBM10 expression and each analysis result. Figure S3: the correlation between RBM10 and different molecular subtypes in pan-cancer. (A) The expression of RBM10 was significantly associated with molecular subtypes in BRCA, HNSC, KIRP, LGG, READ, OV, LUSC, and PRAD. (B) The RBM10 expression was not associated with molecular subtypes in ACC, COAD, ESCA, GBM, LIHC, PCPG, and SKCM. (C) The expression level of RBM10 was not associated with tumor stages in BLCA, BRCA, CESC, CHOL, COAD, DLBC, ESCA, HNSC, KIRC, KIRP, LUAD, LUSC, READ, STAD, TGCT, THCA, and UCEC. Figure S4: the prognosis analysis of RBM10 in tumors. (A, B) The correlation analysis of RBM10 expression with overall survival (OS, (A)) and relapse-free survival (RFS, (B)) in patients with different cancer types from the TCGA database of the Kaplan-Meier plotter. The median RBM10 expression was used to classify patients into high expression and low expression groups. p < 0.05 was statistically significant. Figure S5: (A–D) forest map showed the univariate Cox regression results of RBM10 for OS (A), DSS (B), DFI (C), and PFI (D) in multiple cancers. Figure S6: the expression level of RBM10 in the patients with RBM10 wild type (RBM10 WT) or RBM10 mutation (RBM10 Mut) of LUAD, BLCA, and PAAD. Figure S7: the correlation of RBM10 expression with CNV. (A) The association between RBM10 mRNA expression and CNV in different cancers using GSCA. Blue bubbles represented negative corr [file 7654937.f1.zip › Graphical Abstract (1).pdf]

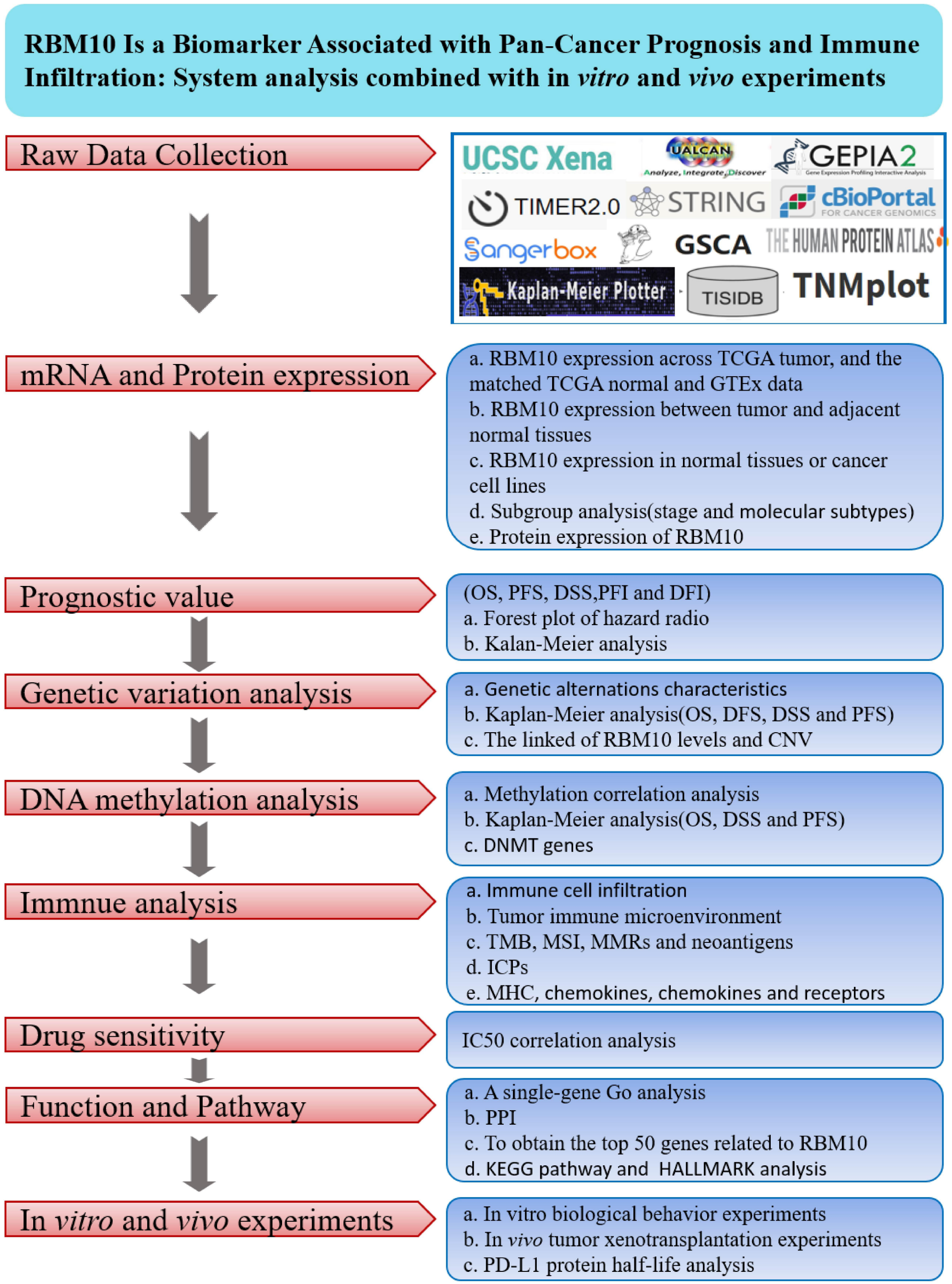
RBM10 Is a Biomarker Associated with Pan-Cancer Prognosis and Immune Infiltration: System analysis combined with in vitro and vivo experiments
Raw Data Collection
UCSC Xena
UALCAN
GEPIA2
TIMER2.0
STRING
cBioPortal
sangerbox
GSCA
THE HUMAN PROTEIN ATLAS
Kaplan-Meier Plotter
TISIDB
TNMplot
mRNA and Protein expression
a. RBM10 expression across TCGA tumor, and the matched TCGA normal and GTEx data
b. RBM10 expression between tumor and adjacent normal tissues
c. RBM10 expression in normal tissues or cancer cell lines
d. Subgroup analysis(stage and molecular subtypes)
e. Protein expression of RBM10
Prognostic value
(OS, PFS, DSS,PFI and DFI)
a. Forest plot of hazard radio
b. Kalan-Meier analysis
Genetic variation analysis
a. Genetic alternations characteristics
b. Kaplan-Meier analysis(OS, DFS, DSS and PFS)
c. The linked of RBM10 levels and CNV
DNA methylation analysis
a. Methylation correlation analysis
b. Kaplan-Meier analysis(OS, DSS and PFS)
c. DNMT genes
Immnue analysis
a. Immune cell infiltration
b. Tumor immune microenvironment
c. TMB, MSI, MMRs and neoantigens
d. ICPs
e. MHC, chemokines, chemokines and receptors
Drug sensitivity
IC50 correlation analysis
Function and Pathway
a. A single-gene Go analysis
b. PPI
c. To obtain the top 50 genes related to RBM10
d. KEGG pathway and HALLMARK analysis
In vitro and vivo experiments
a. In vitro biological behavior experiments
b. In vivo tumor xenotransplantation experiments
c. PD-L1 protein half-life analysis
