# Supplementary material for: RBM10 Is a Biomarker Associated with Pan-Cancer Prognosis and Immune Infiltration: System Analysis Combined with In Vitro and Vivo Experiments
Source: Oxid Med Cell Longev. 2022 Nov 28;2022:7654937. doi: 10.1155/2022/7654937 (PMC11401663; doi:10.1155/2022/7654937)
Supplement: Supplementary Materials — Figure S1: RBM10 mRNA expression in human normal tissues and different cancer cells. (A) RBM10 mRNA expression in various normal tissues was analyzed using the HPA/GTEx/FANTOM5 database. (B) The mRNA expression level of RBM10 in various cancer cells was analyzed from the data of the CCLE database. Figure S2: the protein expression of RBM10 in pan-cancer and a single-gene GO analysis of RBM10. (A) RBM10 protein level in different tumors and normal adjacent tissues through the CPTAC database. (B) SangerBox showed the positively association between RBM10 expression and each analysis result. Figure S3: the correlation between RBM10 and different molecular subtypes in pan-cancer. (A) The expression of RBM10 was significantly associated with molecular subtypes in BRCA, HNSC, KIRP, LGG, READ, OV, LUSC, and PRAD. (B) The RBM10 expression was not associated with molecular subtypes in ACC, COAD, ESCA, GBM, LIHC, PCPG, and SKCM. (C) The expression level of RBM10 was not associated with tumor stages in BLCA, BRCA, CESC, CHOL, COAD, DLBC, ESCA, HNSC, KIRC, KIRP, LUAD, LUSC, READ, STAD, TGCT, THCA, and UCEC. Figure S4: the prognosis analysis of RBM10 in tumors. (A, B) The correlation analysis of RBM10 expression with overall survival (OS, (A)) and relapse-free survival (RFS, (B)) in patients with different cancer types from the TCGA database of the Kaplan-Meier plotter. The median RBM10 expression was used to classify patients into high expression and low expression groups. p < 0.05 was statistically significant. Figure S5: (A–D) forest map showed the univariate Cox regression results of RBM10 for OS (A), DSS (B), DFI (C), and PFI (D) in multiple cancers. Figure S6: the expression level of RBM10 in the patients with RBM10 wild type (RBM10 WT) or RBM10 mutation (RBM10 Mut) of LUAD, BLCA, and PAAD. Figure S7: the correlation of RBM10 expression with CNV. (A) The association between RBM10 mRNA expression and CNV in different cancers using GSCA. Blue bubbles represented negative corr [file 7654937.f1.zip › STR reports of H3255 cell lines.pdf]

H3255 细胞 STR 检验报告

鉴定日期：2021 年 4 月 21 日

一、 检材：

H3255 细胞沉淀：

要求鉴定该株细胞是否为单一来源细胞系，是否存在交叉污染现象。

二、 检材处理和检验方法：

取适量检材用 Chelex100 提取 DNA，采用 Powerplex™16 ID System STR 复合扩增试剂盒进行扩增，在 ABI 3130xl 型遗传分析仪上对 STR 位点和性别基因 Amelogenin 进行检测。

三、 检验结果：

实验中阳性及阴性对照结果均正确。

该细胞株的 STR 位点和 Amelogenin 位点的基因分型结果见附表，分型图谱见附图。

四、 分析说明：

用 Gene Mapper v3.2 software(ABI)对各 STR 位点进行基因型分析。该细胞 DNA 扩增后图谱清晰，分型结果良好。

五、 检验结论：

①该株细胞 DNA 进行细胞 STR 分型结果显示，在各个基因座均未出现三等位基因现象，细胞中没有发现人类细胞交叉污染。

②该株细胞 STR 分型 ATCC、DSMZ 和 JCRB 细胞库中找到与其细胞分型 100%相匹配的细胞，细胞名称为 H3255 等。

Result of STR matching analysis by your data.

- DSMZ Profile Database -

A graphical presentation is shown at the bottom of this page.

EV	Cell No.	Cell name	Locus names									Figures
			D5S818	D13S317	D7S820	D16S539	VWA	TH01	AM	TPOX	CSF1PO	
	Query (Your Cell)		11, 12	11, 13	10, 13	8, 8	16, 16	6, 6	X, X	8, 11	11, 14	
1.00(36/36)	CRL-2882	NCI-H3255	11,12	11,13	10,13	8,8	16,16	6,6	X,X	8,11	11,14	-

附表：H3255 细胞的 STR 位点和 Amelogenin 位点的基因分型结果

H3255 细胞		
Marker	Allele 1	Allele 2
D19S433	13	15
D5S818	11	12
D21S11	27	29
D18S51	13	19
AMEL	X	X
D3S1358	16	17
D13S317	11	13
D7S820	10	13
D16S539	8	8
CSF1PO	11	14
vWA	16	16
D8S1179	13	14
TPOX	8	11
TH01	6	6
D2S1338	19	21
FGA	23	23

H3255 的分型图谱

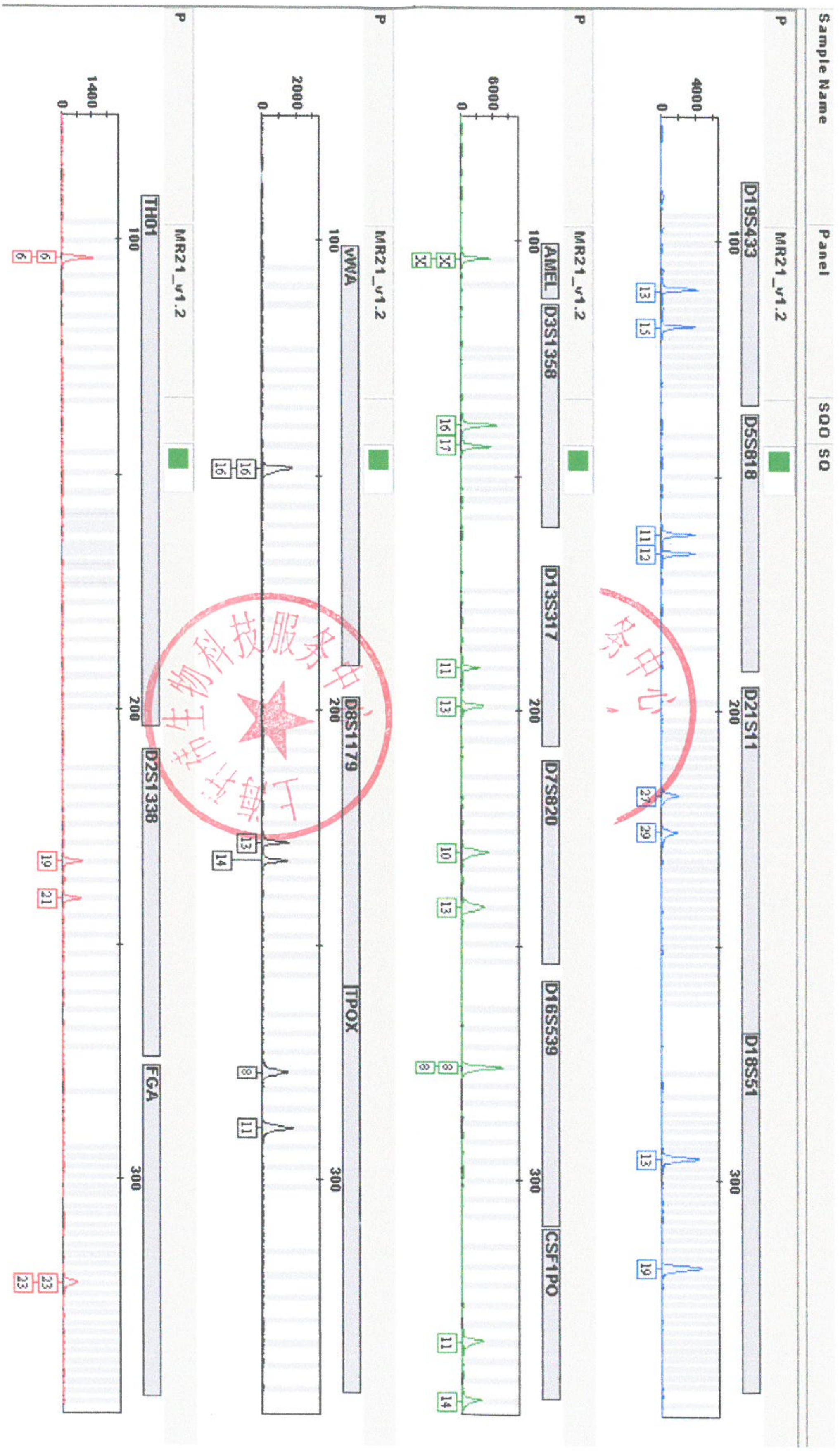
Sample Name
Panel
SQO
SQ
MR21_v1.2
D19S433
D5S818
D21S11
D18S51
AMEL
D3S1358
D13S317
D7S820
D16S539
CSF1PO
vWA
D8S1179
TPOX
TH01
D2S1338
FGA
