# Supplementary material for: RBM10 Is a Biomarker Associated with Pan-Cancer Prognosis and Immune Infiltration: System Analysis Combined with In Vitro and Vivo Experiments
Source: Oxid Med Cell Longev. 2022 Nov 28;2022:7654937. doi: 10.1155/2022/7654937 (PMC11401663; doi:10.1155/2022/7654937)
Supplement: Supplementary Materials — Figure S1: RBM10 mRNA expression in human normal tissues and different cancer cells. (A) RBM10 mRNA expression in various normal tissues was analyzed using the HPA/GTEx/FANTOM5 database. (B) The mRNA expression level of RBM10 in various cancer cells was analyzed from the data of the CCLE database. Figure S2: the protein expression of RBM10 in pan-cancer and a single-gene GO analysis of RBM10. (A) RBM10 protein level in different tumors and normal adjacent tissues through the CPTAC database. (B) SangerBox showed the positively association between RBM10 expression and each analysis result. Figure S3: the correlation between RBM10 and different molecular subtypes in pan-cancer. (A) The expression of RBM10 was significantly associated with molecular subtypes in BRCA, HNSC, KIRP, LGG, READ, OV, LUSC, and PRAD. (B) The RBM10 expression was not associated with molecular subtypes in ACC, COAD, ESCA, GBM, LIHC, PCPG, and SKCM. (C) The expression level of RBM10 was not associated with tumor stages in BLCA, BRCA, CESC, CHOL, COAD, DLBC, ESCA, HNSC, KIRC, KIRP, LUAD, LUSC, READ, STAD, TGCT, THCA, and UCEC. Figure S4: the prognosis analysis of RBM10 in tumors. (A, B) The correlation analysis of RBM10 expression with overall survival (OS, (A)) and relapse-free survival (RFS, (B)) in patients with different cancer types from the TCGA database of the Kaplan-Meier plotter. The median RBM10 expression was used to classify patients into high expression and low expression groups. p < 0.05 was statistically significant. Figure S5: (A–D) forest map showed the univariate Cox regression results of RBM10 for OS (A), DSS (B), DFI (C), and PFI (D) in multiple cancers. Figure S6: the expression level of RBM10 in the patients with RBM10 wild type (RBM10 WT) or RBM10 mutation (RBM10 Mut) of LUAD, BLCA, and PAAD. Figure S7: the correlation of RBM10 expression with CNV. (A) The association between RBM10 mRNA expression and CNV in different cancers using GSCA. Blue bubbles represented negative corr [file 7654937.f1.zip › STR reports of H827 cell lines.pdf]

HCC827 细胞 STR 鉴定报告

一、 材料处理和检验方法

取适量 **HCC827** 细胞(编号 XB0421-1811,1×10^6)使用 Microread Genomic DNA Kit 提取 DNA，采用 MicroreaderTM21 ID System 扩增 20 个 STR 位点和性别鉴定位点，使用 ABI 3730xl 型遗传分析仪进行 PCR 产物检测，使用 GeneMapperID-X 软件（Applied Biosystems）对检测结果进行分析，并与 ATCC、DSMZ、JCRB、Cellosaurus 等数据库进行比对。

二、 检测结果

实验中阴性及阳性对照结果均正确。

HCC827（编号 XB0421-1811）细胞株的 STR 位点和 Amelogenin 位点的基因分型结果见附表，分型图谱见附图。

三、 分析说明

HCC827（编号 XB0421-1811）细胞株基因组 DNA 扩增后图谱清晰，分型结果良好。

四、 检验结论

1. **HCC827**（编号 XB0421-1811）细胞株 DNA 进行细胞 STR 分型结果显示，细胞株中未发现人类细胞交叉污染。
2. 该细胞株 DNA 分型在细胞库中找到与其细胞分型 100.00%相匹配的细胞株，细胞株名称为 **HCC827**。

Procell www.procell.com.cn

附表 1： 细胞株HCC827 的 STR 位点和 Amelogenin 位点的基因分型结果

STR Loci	样品名称：XB0421-1811	数据库名称：HCC827
Amelogenin	X,X	X
CSF1PO	11,11	11
D2S1338	17,24	17,24
D3S1358	17,17	17
D5S818	12,12	12
D7S820	11,12	11,12
D8S1179	12,12	12
D13S317	9,9	9
D16S539	12,12	12
D18S51	13,13	13
D19S433	14,14	14
D21S11	31,31	31
FGA	22,24	22,24
PentaD	14,14	14
PentaE	20,20	20
TH01	6,6	6
TPOX	8,8	8
vWA	18,18	18
D1S1656		
D6S1043	11,12	
D12S391	17,17	
D2S441	11,11.3	
Cellosaurus 数据库匹配度 100.00%，匹配位点数 17（https://web.expasy.org/cellosaurus-str-search/）		

Procell
www.procell.com.cn

备注：

1. 根据国际细胞鉴定委员会(ICLAC)制定的细胞 STR 鉴定标准，细胞系的匹配度≥80% 时，认为它们具有相关性，即衍生于共同的祖先细胞；匹配度在 55% 至 80% 之间，需要进一步验证相关性；小于 55%，表明两者不具有相关性。
2. 图谱有效峰为真实的 PCR 条带;小峰和非特异性条带在计算中忽略不计。
3. STR 数据比对结果默认 ExPASy，数据来源包括 ATCC，DSMZ，JCRB 等细胞库以及文献和资料记载，数据库入口 https://web.expasy.org/cellosaurus-str-search/。

Copyright ©2019-2020 Procell Life Science&Technology Co.,Ltd. All Rights Reserved

附图 1： HCC827 细胞 STR 位点和 Amelogenin 位点的基因分型结果

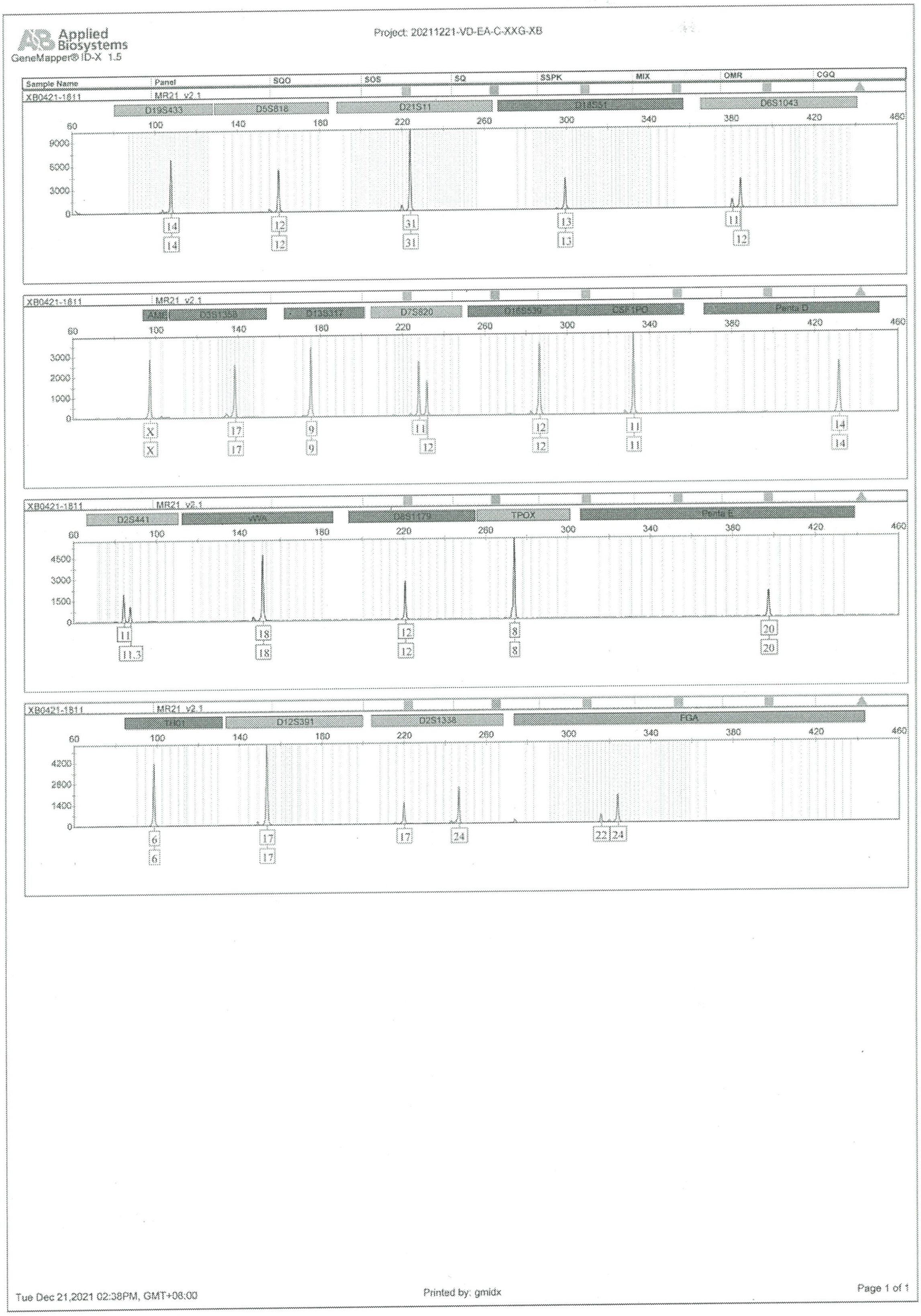

销售电话：400-650-3656
企业 QQ：4006503656
销售邮箱：sales@procell.com.cn
官方网站：www.procell.com.cn
