# Supplementary material for: RBM10 Is a Biomarker Associated with Pan-Cancer Prognosis and Immune Infiltration: System Analysis Combined with In Vitro and Vivo Experiments
Source: Oxid Med Cell Longev. 2022 Nov 28;2022:7654937. doi: 10.1155/2022/7654937 (PMC11401663; doi:10.1155/2022/7654937)
Supplement: Supplementary Materials — Figure S1: RBM10 mRNA expression in human normal tissues and different cancer cells. (A) RBM10 mRNA expression in various normal tissues was analyzed using the HPA/GTEx/FANTOM5 database. (B) The mRNA expression level of RBM10 in various cancer cells was analyzed from the data of the CCLE database. Figure S2: the protein expression of RBM10 in pan-cancer and a single-gene GO analysis of RBM10. (A) RBM10 protein level in different tumors and normal adjacent tissues through the CPTAC database. (B) SangerBox showed the positively association between RBM10 expression and each analysis result. Figure S3: the correlation between RBM10 and different molecular subtypes in pan-cancer. (A) The expression of RBM10 was significantly associated with molecular subtypes in BRCA, HNSC, KIRP, LGG, READ, OV, LUSC, and PRAD. (B) The RBM10 expression was not associated with molecular subtypes in ACC, COAD, ESCA, GBM, LIHC, PCPG, and SKCM. (C) The expression level of RBM10 was not associated with tumor stages in BLCA, BRCA, CESC, CHOL, COAD, DLBC, ESCA, HNSC, KIRC, KIRP, LUAD, LUSC, READ, STAD, TGCT, THCA, and UCEC. Figure S4: the prognosis analysis of RBM10 in tumors. (A, B) The correlation analysis of RBM10 expression with overall survival (OS, (A)) and relapse-free survival (RFS, (B)) in patients with different cancer types from the TCGA database of the Kaplan-Meier plotter. The median RBM10 expression was used to classify patients into high expression and low expression groups. p < 0.05 was statistically significant. Figure S5: (A–D) forest map showed the univariate Cox regression results of RBM10 for OS (A), DSS (B), DFI (C), and PFI (D) in multiple cancers. Figure S6: the expression level of RBM10 in the patients with RBM10 wild type (RBM10 WT) or RBM10 mutation (RBM10 Mut) of LUAD, BLCA, and PAAD. Figure S7: the correlation of RBM10 expression with CNV. (A) The association between RBM10 mRNA expression and CNV in different cancers using GSCA. Blue bubbles represented negative corr [file 7654937.f1.zip › Supplementaly Tables.pdf]

Tables S1~S8

Table S1 List of abbreviations

**Abbreviations**	**Full names**
RBM10	RNA binding motif protein 10
ICP	immune checkpoint
RBP	RNA binding motif protein
LUAD	lung adenocarcinoma
HCC	hepatocellular carcinoma
TCGA	the Cancer Genome Atlas
GTEx	the Genotype-Tissue Expression
GEPIA2	the Gene Expression Profiling Interactive Analysis2
KM	Kaplan-Meier plotter
CNV	copy number variations
TIICs	tumor-infiltrating immune cells
TIMER	the Tumor Immune Estimation Resource
TISIDB	tumor-immune system interactions
TMB	tumor mutation burden
MSI	microsatellite instability
GSEA	The Gene Set Enrichment Analysis
HPA	Human Protein Atlas
ACC	Adrenocortical carcinoma
BLCA	Bladder Urothelial Carcinoma
BRCA	Breast invasive carcinoma
CESC	Cervical squamous cell carcinoma and endocervical adenocarcinoma
CHOL	Cholangio carcinoma
COAD	Colon adenocarcinoma
DLBC	Lymphoid Neoplasm Diffuse Large B-cell Lymphoma
ESCA	Esophageal carcinoma
GBM	Glioblastoma multiforme
HNSC	Head and Neck squamous cell carcinoma
KICH	Kidney Chromophobe
KIRC	Kidney renal clear cell carcinoma
KIRP	Kidney renal papillary cell carcinoma
LAML	Acute Myeloid Leukemia
LGG	Brain Lower Grade Glioma
LIHC	Liver hepatocellular carcinoma
LUSC	Lung squamous cell carcinoma
MESO	Mesothelioma
OV	Ovarian serous cystadenocarcinoma
PAAD	Pancreatic adenocarcinoma
PCPG	Pheochromocytoma and Paraganglioma

PRAD	Prostate adenocarcinoma
READ	Rectum adenocarcinoma
SARC	Sarcoma
SKCM	Skin Cutaneous Melanoma
STAD	Stomach adenocarcinoma
TGCT	Testicular Germ Cell Tumors
THCA	Thyroid carcinoma
THYM	Thymoma
UCEC	Uterine Corpus Endometrial Carcinoma
UCS	Uterine Carcinosarcoma
UVM	Uveal Melanoma
CPTAC	Clinical Proteomic Tumor Analysis Consortium
OS	overall survival
DFS	disease-free survival
RFS	relapse free survival
DFI	disease free interval
PFI	progress-free interval
DSS	disease-specific survival
MHC	major histocompatibility complex
GDSC	the Genomics of Drug Sensitivity in Cancer
CTRP	the Cancer Therapeutics Response Portal
PPI	the protein-protein interaction network
KEGG	the Kyoto Encyclopedia of Genes and Genomes
FBS	fetal bovine serum
CCK-8	the Cell Counting Kit-8
WB	Western blotting
RIPA	radio-immunoprecipitation assay
SDS-PAGE	sodium dodecyl sulfate-polyacrylamide gels
PVDF	polyvinylidene fluoride
RT	room temperature
HRP	horseradish peroxidase
ECL	electrochemiluminescence
CHX	cycloheximide
IHC	Immunohistochemistry
CCLE	the Cancer Cell Line Encyclopedia
SCLC	small cell lung cancer
GO	Gene Ontology
HR	Hazard Ratio
DNMT	DNA methyl-transferase
TIDE	the Tumor Immune Dysfunction and Exclusion
ICB	immune checkpoint blockade
MMRs	mismatch repairs

Table S2 A single- gene GO analysis of RBM10 by using SangerBox database.

GO ID	Namespace	Name	RrefDataBase
GO:0000122	biological_process	negative regulation of transcription by RNA polymerase II	GO_REF:0000107
GO:0000381	biological_process	regulation of alternative mRNA splicing, via spliceosome	GO_REF:0000002
GO:0003723	molecular_function	RNA binding	PMID:22658674
GO:0005515	molecular_function	protein binding	PMID:22365833
GO:0005515	molecular_function	protein binding	PMID:22365833
GO:0005515	molecular_function	protein binding	PMID:22365833
GO:0005515	molecular_function	protein binding	PMID:22365833
GO:0005515	molecular_function	protein binding	PMID:22365833
GO:0005515	molecular_function	protein binding	PMID:22365833
GO:0005515	molecular_function	protein binding	PMID:22365833
GO:0005515	molecular_function	protein binding	PMID:22365833
GO:0005515	molecular_function	protein binding	PMID:22365833
GO:0005515	molecular_function	protein binding	PMID:22365833
GO:0005515	molecular_function	protein binding	PMID:22365833
GO:0005515	molecular_function	protein binding	PMID:22365833
GO:0005515	molecular_function	protein binding	PMID:25416956
GO:0005515	molecular_function	protein binding	PMID:25416956
GO:0005515	molecular_function	protein binding	PMID:26871637
GO:0005515	molecular_function	protein binding	PMID:26871637
GO:0005515	molecular_function	protein binding	PMID:26871637
GO:0005515	molecular_function	protein binding	PMID:26871637
GO:0005515	molecular_function	protein binding	PMID:26871637
GO:0005515	molecular_function	protein binding	PMID:26871637
GO:0005515	molecular_function	protein binding	PMID:26871637
GO:0005515	molecular_function	protein binding	PMID:26871637
GO:0005515	molecular_function	protein binding	PMID:26871637
GO:0005515	molecular_function	protein binding	PMID:26871637
GO:0005515	molecular_function	protein binding	PMID:26871637
GO:0005515	molecular_function	protein binding	PMID:26871637
GO:0005515	molecular_function	protein binding	PMID:26871637
GO:0005515	molecular_function	protein binding	PMID:26871637
GO:0005515	molecular_function	protein binding	PMID:26871637
GO:0005515	molecular_function	protein binding	PMID:26871637
GO:0005515	molecular_function	protein binding	PMID:29021621

GO:0005634	cellular_component	nucleus	GO_REF:0000054
GO:0006397	biological_process	mRNA processing	GO_REF:0000037
GO:0008150	biological_process	biological_process	GO_REF:0000015
GO:0008285	biological_process	negative regulation of cell population proliferation	GO_REF:0000107
GO:0008380	biological_process	RNA splicing	GO_REF:0000037
GO:0016607	cellular_component	nuclear speck	GO_REF:0000052
GO:0032991	cellular_component	protein-containing complex	PMID:17707232
GO:0034393	biological_process	positive regulation of smooth muscle cell apoptotic process	GO_REF:0000107
GO:0035198	molecular_function	miRNA binding	PMID:28431233
GO:0042802	molecular_function	identical protein binding	PMID:22365833
GO:0046872	molecular_function	metal ion binding	GO_REF:0000037
GO:0048025	biological_process	negative regulation of mRNA splicing, via spliceosome	GO_REF:0000107
GO:0070935	biological_process	3'-UTR-mediated mRNA stabilization	GO_REF:0000107

Table S3 The correlation between RBM10 level and CNV via GSCA database.

Cancer type	Symbol	Spm	FDR
ACC	RBM10	0.312735027	0.013874786
BLCA	RBM10	0.224298337	≤0.001
BRCA	RBM10	0.032695301	0.321872128
CESC	RBM10	0.198174475	0.001288828
CHOL	RBM10	-0.026649503	0.9305401
COAD	RBM10	0.049234885	0.504993157
DLBC	RBM10	0.060793573	0.873914456
ESCA	RBM10	0.354271778	2.36085E-06
GBM	RBM10	-0.089842142	0.391369581
HNSC	RBM10	0.252786919	1.48826E-08
KICH	RBM10	-0.105345568	0.553786178
KIRC	RBM10	0.047850676	0.360338073
KIRP	RBM10	-0.207319066	0.001085239
LAML	RBM10	-0.185320597	0.192709604
LGG	RBM10	-0.161183684	≤0.001
LIHC	RBM10	-0.023201043	0.726038703
LUAD	RBM10	0.124568482	0.007217392
LUSC	RBM10	0.141898069	0.002227904
MESO	RBM10	-0.21891717	0.09015045
OV	RBM10	0.252926363	≤0.001
PAAD	RBM10	0.057111568	0.548916797
PCPG	RBM10	-0.097291225	0.353099366
PRAD	RBM10	-0.137435242	0.006836241
READ	RBM10	0.015965979	0.921722263
SARC	RBM10	0.126481744	0.0648465
SKCM	RBM10	0.097503107	0.088977429
STAD	RBM10	0.202111167	≤0.001
TGCT	RBM10	0.025589368	0.817794155
THCA	RBM10	-0.047419046	0.51155795
THYM	RBM10	0.232094649	0.050375431
UCEC	RBM10	0.121894394	0.160430524
UCS	RBM10	0.522719894	≤0.001
UVM	RBM10	-0.074757811	0.698345119

Table S4 The positively correlation of RBM10 mRNA level with the drug sensitivity in GDSC database.

Symbol	Drug	Cor	FDR
RBM10	Trametinib	0.33515606	≤0.001
RBM10	17-AAG	0.306063151	≤0.001
RBM10	PD-0325901	0.279433628	≤0.001
RBM10	RDEA119	0.274006792	≤0.001
RBM10	Cetuximab	0.263296319	5.96728E-13
RBM10	Afatinib	0.25741971	2.36304E-14
RBM10	XAV939	0.252731385	1.34369E-12
RBM10	selumetinib	0.245232401	5.00115E-13
RBM10	Dasatinib	0.241804706	≤0.001
RBM10	Gefitinib	0.236330592	1.31081E-10
RBM10	Docetaxel	0.220374884	9.82831E-10
RBM10	WZ-1-84	0.217648188	≤0.001
RBM10	Erlotinib	0.210184033	≤0.001
RBM10	Lapatinib	0.208835612	≤0.001
RBM10	RO-3306	0.197411938	3.98458E-07
RBM10	WH-4-023	0.191011399	0.003437132
RBM10	Bleomycin (50 uM)	0.186339373	9.01569E-08
RBM10	CI-1040	0.183300545	1.83839E-06
RBM10	Bortezomib	0.179601091	0.005132911
RBM10	JNK Inhibitor VIII	0.178807719	4.10172E-06
RBM10	FTI-277	0.174146862	5.80771E-06
RBM10	A-770041	0.161782232	0.016783069
RBM10	TGX221	0.159664743	0.007947479
RBM10	Saracatinib	0.156994822	0.034700752
RBM10	CCT007093	0.152379527	≤0.001
RBM10	BIRB 0796	0.138612216	0.002254472
RBM10	BMS-708163	0.136349469	≤0.001
RBM10	SGC0946	0.136016807	0.001808401
RBM10	CHIR-99021	0.132702314	≤0.001
RBM10	AKT inhibitor VIII	0.131008191	0.001384013
RBM10	AS601245	0.123783561	0.005327948
RBM10	Bicalutamide	0.121114867	0.002760143
RBM10	IOX2	0.120329005	0.015182815
RBM10	GSK1904529A	0.11830902	0.00470482
RBM10	AMG-706	0.114182645	0.018204197
RBM10	SB 216763	0.107107733	0.020348243
RBM10	KIN001-055	0.102351939	0.033782392
RBM10	CCT018159	0.097476158	0.031330674
RBM10	Bryostatin 1	0.094015486	0.042317895

RBM10	Bosutinib	0.093712751	0.038725332
RBM10	Midostaurin	0.092705232	0.026049326
RBM10	FH535	0.087742701	0.043458613

Table S5 The negatively correlation of RBM10 mRNA level with the drug sensitivity in GDSC database.

Symbol	Drug	Cor	FDR
RBM10	GSK1070916	-0.344712733	7.36473E-25
RBM10	NPK76-II-72-1	-0.337091794	9.45467E-25
RBM10	Navitoclax	-0.336103607	1.61576E-22
RBM10	BX-912	-0.331763642	8.75494E-24
RBM10	I-BET-762	-0.303893649	5.0189E-20
RBM10	XMD13-2	-0.297463995	6.5427E-19
RBM10	FK866	-0.296088392	2.47342E-18
RBM10	Vorinostat	-0.290087681	9.17831E-17
RBM10	CAY10603	-0.285490994	2.55043E-17
RBM10	Methotrexate	-0.282383795	6.20385E-16
RBM10	QL-X-138	-0.281210373	1.4861E-16
RBM10	OSI-027	-0.280209071	1.47216E-16
RBM10	AR-42	-0.277800489	2.41113E-16
RBM10	WZ3105	-0.276915172	1.89577E-16
RBM10	PHA-793887	-0.268822296	1.04965E-15
RBM10	JW-7-24-1	-0.266064818	3.39388E-15
RBM10	THZ-2-102-1	-0.26519536	8.65848E-15
RBM10	PIK-93	-0.260565433	9.524E-15
RBM10	Belinostat	-0.255916819	1.87499E-13
RBM10	TPCA-1	-0.253191791	5.94239E-14
RBM10	Tubastatin A	-0.252651839	9.39869E-14
RBM10	TG101348	-0.251599906	1.07623E-13
RBM10	PI-103	-0.250628076	2.30724E-13
RBM10	CUDC-101	-0.249149815	5.42212E-13
RBM10	CX-5461	-0.247871695	5.49682E-13
RBM10	Genentech Cpd 10	-0.245053377	8.61057E-13
RBM10	Ispinesib Mesylate	-0.239361959	3.11728E-12
RBM10	CP466722	-0.234305451	5.81896E-12
RBM10	GSK690693	-0.234041205	1.09655E-11
RBM10	UNC0638	-0.233057749	2.6846E-12
RBM10	QL-XI-92	-0.231629714	1.3291E-11
RBM10	XMD14-99	-0.224407185	1.50429E-10
RBM10	KIN001-102	-0.223387976	5.63454E-11
RBM10	BMS345541	-0.214083454	4.40317E-10
RBM10	SNX-2112	-0.203704803	3.90692E-09
RBM10	YM201636	-0.203277935	4.84727E-09
RBM10	AT-7519	-0.201803988	4.19909E-09
RBM10	TL-2-105	-0.20149619	8.55356E-09
RBM10	ZSTK474	-0.200623756	7.15436E-09

RBM10	MPS-1-IN-1	-0.198994196	1.79848E-08
RBM10	GSK2126458	-0.194039405	3.36032E-08
RBM10	5-Fluorouracil	-0.192420208	3.4378E-08
RBM10	ZM-447439	-0.190060264	1.82776E-06
RBM10	NG-25	-0.18667764	6.32807E-08
RBM10	KIN001-244	-0.184837045	1.3456E-07
RBM10	KIN001-236	-0.184245559	1.48117E-07
RBM10	IPA-3	-0.181339553	7.86957E-07
RBM10	BIX02189	-0.17842721	3.18934E-07
RBM10	Camptothecin	-0.176984588	4.35103E-06
RBM10	Masitinib	-0.174889218	8.01886E-07
RBM10	KIN001-260	-0.172071954	8.24409E-07
RBM10	NSC-207895	-0.17060809	≤0.001
RBM10	ZG-10	-0.170072073	0.002589098
RBM10	Y-39983	-0.169743246	1.61277E-06
RBM10	QL-XII-61	-0.169484628	0.002625051
RBM10	LAQ824	-0.168274414	5.19998E-06
RBM10	Foretinib	-0.167839054	4.39983E-06
RBM10	TL-1-85	-0.165750789	1.92185E-06
RBM10	Phenformin	-0.165540938	3.44799E-06
RBM10	TAK-715	-0.165265148	2.34512E-06
RBM10	BHG712	-0.162053032	3.44253E-06
RBM10	CAL-101	-0.157737233	9.6871E-06
RBM10	PAC-1	-0.155276986	≤0.001
RBM10	QL-XII-47	-0.153929893	≤0.001
RBM10	MP470	-0.151782304	≤0.001
RBM10	CEP-701	-0.136966718	≤0.001
RBM10	Etoposide	-0.136631218	≤0.001
RBM10	KIN001-270	-0.132749325	≤0.001
RBM10	HG-5-113-01	-0.132728979	0.031197657
RBM10	GSK429286A	-0.131806199	≤0.001
RBM10	Mitomycin C	-0.130953269	0.002427446
RBM10	AICAR	-0.124072947	0.001149139
RBM10	THZ-2-49	-0.121993792	≤0.001
RBM10	T0901317	-0.121791425	0.001384339
RBM10	SB52334	-0.120857742	0.002339877
RBM10	AZD8055	-0.117604438	0.002101619
RBM10	OSI-930	-0.11722396	0.001552765
RBM10	XMD15-27	-0.116164313	0.004603447
RBM10	BX-795	-0.112905157	0.005790896
RBM10	SN-38	-0.111566031	0.004463161
RBM10	JQ1	-0.110989336	0.004811212
RBM10	AC220	-0.105490957	0.010918245

RBM10	BAY 61-3606	-0.103813266	0.008027306
RBM10	STF-62247	-0.102252772	0.008199532
RBM10	VNLG/124	-0.101046528	0.009167674
RBM10	JQ12	-0.097750267	0.041441842
RBM10	KIN001-266	-0.093921093	0.031237833
RBM10	Shikonin	-0.09242589	0.04747328
RBM10	AP-24534	-0.086272292	0.029863094
RBM10	AS605240	-0.084675871	0.036011557
RBM10	EKB-569	-0.078372205	0.045065163

Table S6 A significant difference about the relationship of RBM10 level with drug sensitivity in CTRP was observed.

Symbol	Drug	Cor	FDR
RBM10	abiraterone	0.285420004	0.017837743
RBM10	PD 153035	0.189418715	≤0.001
RBM10	vandetanib	0.186683857	≤0.001
RBM10	VAF-347	0.17680274	0.002006369
RBM10	saracatinib	0.171559745	≤0.001
RBM10	erlotinib	0.158523871	≤0.001
RBM10	dasatinib	0.150351927	≤0.001
RBM10	trametinib	0.129452006	0.061372738
RBM10	selumetinib	0.116119246	0.008651008
RBM10	BRD-K17060750	0.102129039	0.0305061
RBM10	PD318088	0.100382852	0.022569762
RBM10	BRD-K30748066	-0.513441379	0.001271158
RBM10	GSK-J4	-0.483463905	≤0.001
RBM10	LY-2183240	-0.450286687	8.04206E-38
RBM10	GSK461364	-0.437857673	5.95352E-35
RBM10	triazolothiadiazine	-0.436519594	3.10951E-36
RBM10	teniposide	-0.425984318	1.52736E-17
RBM10	vincristine	-0.423797526	9.45121E-35
RBM10	tivantinib	-0.410386994	4.33722E-15
RBM10	PX-12	-0.408744086	3.3888E-30
RBM10	parbendazole	-0.407247393	2.98448E-31
RBM10	necrosulfonamide	-0.404423256	1.98945E-20
RBM10	cytarabine hydrochloride	-0.401463128	8.81933E-30
RBM10	BI-2536	-0.401256456	1.78278E-30
RBM10	KX2-391	-0.400193887	6.75602E-29
RBM10	docetaxel	-0.398946733	3.25738E-13
RBM10	KPT185	-0.398186823	2.08689E-21
RBM10	ceranib-2	-0.395778275	3.60092E-29
RBM10	YK 4-279	-0.395355178	9.28117E-29
RBM10	SB-743921	-0.393153998	4.55764E-29
RBM10	clofarabine	-0.391772396	1.84653E-28
RBM10	BRD-K70511574	-0.390927692	1.13426E-27
RBM10	doxorubicin	-0.387820176	2.75049E-28
RBM10	SB-225002	-0.387385821	4.02774E-28
RBM10	etoposide	-0.384946019	4.48766E-27
RBM10	narciclasine	-0.384584198	3.18426E-26
RBM10	PL-DI	-0.384240301	2.01232E-26
RBM10	KW-2449	-0.382980983	1.08885E-26
RBM10	topotecan	-0.382222276	3.50253E-27

RBM10	axitinib	-0.381966004	3.42982E-26
RBM10	chlorambucil	-0.379913819	3.11633E-25
RBM10	BRD-K34222889	-0.376669565	8.15682E-26
RBM10	FQI-2	-0.376492174	2.99724E-26
RBM10	CD-437	-0.375168015	2.23772E-25
RBM10	piperlongumine	-0.375132712	8.44603E-26
RBM10	rigosertib	-0.372645252	2.80545E-24
RBM10	isoevodiamine	-0.37032306	5.79063E-25
RBM10	belinostat	-0.369186311	1.40907E-12
RBM10	PRIMA-1	-0.366057546	6.02965E-23
RBM10	dinaciclib	-0.364971507	6.23222E-12
RBM10	CHM-1	-0.364061247	3.4535E-24
RBM10	nakiterpiosin	-0.362949457	1.07795E-23
RBM10	gemcitabine	-0.3620733	7.53814E-22
RBM10	tipifarnib-P2	-0.359743865	1.95078E-12
RBM10	ciclopirox	-0.358418769	9.42243E-24
RBM10	mitomycin	-0.355566639	9.71172E-23
RBM10	paclitaxel	-0.353448935	3.28394E-22
RBM10	tozasertib	-0.349054881	0.020994242
RBM10	manumycin A	-0.347679258	7.30502E-22
RBM10	SN-38	-0.346632998	1.96218E-17
RBM10	FQI-1	-0.343372229	3.06967E-11
RBM10	barasertib	-0.343052012	1.04131E-20
RBM10	ML311	-0.342093784	1.00076E-21
RBM10	LRRK2-IN-1	-0.341973003	2.60531E-15
RBM10	BRD-K66453893	-0.341339488	4.79131E-21
RBM10	vorinostat	-0.340223451	8.57979E-21
RBM10	ELCPK	-0.338472274	8.63203E-10
RBM10	ISOX	-0.337925438	1.48205E-20
RBM10	alvocidib	-0.336829432	6.79855E-10
RBM10	BRD-A94377914	-0.336219274	9.06996E-11
RBM10	GW-843682X	-0.335265522	1.43166E-18
RBM10	NSC95397	-0.333876438	6.3853E-18
RBM10	panobinostat	-0.333542346	1.38741E-20
RBM10	PHA-793887	-0.333526479	1.88641E-20
RBM10	BRD-K26531177	-0.332514607	5.65873E-18
RBM10	PF-3758309	-0.332384123	4.26992E-10
RBM10	PF-184	-0.332219802	3.95127E-19
RBM10	alisertib	-0.331194243	1.599E-19
RBM10	decitabine	-0.328200622	6.73862E-20
RBM10	NVP-231	-0.327983985	3.61587E-19
RBM10	apicidin	-0.325990532	1.14731E-19
RBM10	cerulenin	-0.325244545	8.05857E-19

RBM10	leptomycin B	-0.324816289	7.49139E-20
RBM10	pifithrin-mu	-0.324567221	3.98809E-18
RBM10	BRD-K61166597	-0.321695796	2.3529E-17
RBM10	Merck60	-0.319335766	1.28636E-18
RBM10	indisulam	-0.318997072	1.70568E-17
RBM10	NSC48300	-0.318248238	4.36609E-18
RBM10	SNX-2112	-0.315631721	1.92492E-18
RBM10	COL-3	-0.314638114	4.40915E-13
RBM10	3-Cl-AHPC	-0.312700871	1.46468E-17
RBM10	methylstat	-0.312348393	2.87011E-12
RBM10	AT13387	-0.309962501	2.51089E-08
RBM10	omacetaxine mepesuccinate	-0.30887033	1.06729E-12
RBM10	entinostat	-0.305818118	1.0661E-16
RBM10	PAC-1	-0.303889374	7.85386E-16
RBM10	SCH-79797	-0.302278016	2.25888E-16
RBM10	CR-1-31B	-0.302060106	5.25158E-17
RBM10	obatoclax	-0.301938317	2.20954E-16
RBM10	SR-II-138A	-0.299131453	5.53339E-17
RBM10	olaparib	-0.298319296	1.52545E-15
RBM10	NSC632839	-0.297870754	4.351E-16
RBM10	pevonedistat	-0.297210946	3.46295E-15
RBM10	QW-BI-011	-0.294900775	≤0.001
RBM10	BRD-K35604418	-0.291924685	1.00956E-14
RBM10	MK-1775	-0.291400999	4.5294E-15
RBM10	CCT036477	-0.288878097	5.43291E-15
RBM10	ouabain	-0.288411067	3.22087E-15
RBM10	STF-31	-0.288371665	2.76796E-14
RBM10	crizotinib	-0.288336462	7.41356E-15
RBM10	BRD-A86708339	-0.286435916	3.73768E-07
RBM10	curcumin	-0.285343899	1.13827E-14
RBM10	CIL70	-0.280908519	1.38458E-07
RBM10	MST-312	-0.279778685	5.68497E-14
RBM10	tacedinaline	-0.279236599	2.42023E-10
RBM10	daporinad	-0.278572295	1.15531E-11
RBM10	AT7867	-0.277306481	3.01214E-13
RBM10	BRD-K48334597	-0.277235427	0.008332378
RBM10	BIX-01294	-0.275404379	1.31292E-13
RBM10	Compound 7d-cis	-0.271734793	2.32907E-11
RBM10	OSI-930	-0.271723885	6.03553E-11
RBM10	cucurbitacin I	-0.269611852	1.67553E-12
RBM10	brivanib	-0.268267382	6.59125E-12
RBM10	NSC19630	-0.268094586	3.09915E-09
RBM10	WP1130	-0.26791358	2.33808E-12

RBM10	BRD-K66532283	-0.267414706	3.34916E-12
RBM10	SU11274	-0.267314584	3.94186E-12
RBM10	CIL56	-0.266735035	2.46471E-06
RBM10	bardoxolone methyl	-0.266630417	2.6752E-11
RBM10	PF-573228	-0.266091331	1.2171E-12
RBM10	BMS-345541	-0.266045403	1.93892E-12
RBM10	tipifarnib-P1	-0.265246792	2.5896E-12
RBM10	sotrastaurin	-0.264683287	2.80753E-09
RBM10	serdemetan	-0.264383114	6.29661E-12
RBM10	CAY10618	-0.26228606	1.67419E-12
RBM10	JQ-1	-0.261850279	7.83219E-13
RBM10	BRD1812	-0.260395957	7.40139E-12
RBM10	bortezomib	-0.259941063	6.98184E-12
RBM10	marinopyrrole A	-0.259893	6.43998E-08
RBM10	bendamustine	-0.259535559	1.9815E-10
RBM10	I-BET151	-0.25832579	1.25764E-12
RBM10	pazopanib	-0.258166025	8.42967E-12
RBM10	neopeltolide	-0.258110906	0.002525838
RBM10	AZ-3146	-0.258017171	3.41577E-11
RBM10	GMX-1778	-0.257387086	1.53269E-11
RBM10	MGCD-265	-0.252596225	1.70019E-11
RBM10	PRIMA-1-Met	-0.247722152	≤0.001
RBM10	MLN2238	-0.245113553	8.11478E-11
RBM10	ML031	-0.244951448	2.6498E-09
RBM10	GSK525762A	-0.244733649	2.68704E-11
RBM10	AZD7762	-0.243554936	4.18992E-11
RBM10	avrainvillamide	-0.243259273	9.09848E-08
RBM10	isoliquiritigenin	-0.241687324	0.003057436
RBM10	B02	-0.241525661	3.93334E-10
RBM10	BRD-K28456706	-0.240328891	8.95008E-10
RBM10	darinaparsin	-0.240110008	≤0.001
RBM10	gossypol	-0.238493729	6.90589E-10
RBM10	Ki8751	-0.237101023	3.22544E-09
RBM10	HLI 373	-0.233899659	2.05165E-09
RBM10	BRD-K80183349	-0.233615784	6.68949E-10
RBM10	ML210	-0.232263537	1.74634E-09
RBM10	dacarbazine	-0.231863407	7.11994E-10
RBM10	SMER-3	-0.231611403	3.72341E-08
RBM10	methotrexate	-0.23095746	5.44643E-09
RBM10	phloretin	-0.230903782	1.00379E-09
RBM10	nutlin-3	-0.23076353	1.70757E-09
RBM10	tubastatin A	-0.230585959	≤0.001
RBM10	GW-405833	-0.228923161	8.90285E-10

RBM10	foretinib	-0.228779544	5.52119E-09
RBM10	neuronal differentiation inducer III	-0.227926212	1.01459E-09
RBM10	ABT-199	-0.227826035	≤0.001
RBM10	KU-60019	-0.226909498	1.78829E-09
RBM10	erastin	-0.226124573	1.01264E-08
RBM10	N9-isopropylolomoucine	-0.224245369	9.88423E-09
RBM10	fingolimod	-0.223928991	4.07912E-09
RBM10	RITA	-0.223926164	1.03219E-08
RBM10	ABT-737	-0.222894389	5.13174E-08
RBM10	fluorouracil	-0.222486395	3.12529E-09
RBM10	BRD-K92856060	-0.220885597	1.81988E-08
RBM10	tigecycline	-0.220875754	≤0.001
RBM10	SID 26681509	-0.218701374	1.66889E-07
RBM10	TW-37	-0.218179345	2.05963E-08
RBM10	valdecoxib	-0.217971845	1.89028E-08
RBM10	Compound 23 citrate	-0.217835445	1.08958E-08
RBM10	PI-103	-0.21751912	2.21578E-08
RBM10	ETP-46464	-0.217413385	8.44618E-06
RBM10	BRD-K51490254	-0.21728438	8.98492E-08
RBM10	triptolide	-0.217239288	1.01312E-08
RBM10	Ko-143	-0.216846141	1.59632E-08
RBM10	R428	-0.216670003	4.24824E-06
RBM10	BRD-K88742110	-0.216457227	4.1331E-08
RBM10	NSC23766	-0.2164278	2.19548E-08
RBM10	MG-132	-0.215747903	0.00321574
RBM10	oligomycin A	-0.215201502	2.24696E-08
RBM10	SNS-032	-0.213528086	7.24432E-08
RBM10	NVP-BSK805	-0.213088027	2.11475E-08
RBM10	tacrolimus	-0.212770129	1.06652E-07
RBM10	navitoclax	-0.21197321	6.98347E-08
RBM10	TPCA-1	-0.210695213	2.15403E-08
RBM10	CIL55A	-0.20925687	≤0.001
RBM10	momelotinib	-0.209061603	9.77404E-08
RBM10	linifanib	-0.208134075	2.85123E-08
RBM10	BRD-K11533227	-0.208090372	4.79496E-07
RBM10	zebularine	-0.207309535	6.23669E-08
RBM10	epigallocatechin-3-monogallate	-0.206710744	7.67404E-07
RBM10	sunitinib	-0.206323257	7.56591E-08
RBM10	SKI-II	-0.206027627	4.97793E-07
RBM10	sirolimus	-0.204872343	7.35889E-08
RBM10	elocalcitol	-0.202374641	1.05012E-07
RBM10	SRT-1720	-0.201482806	1.21043E-06

RBM10	KU 0060648	-0.201480536	1.79739E-07
RBM10	tretinoin	-0.200274642	2.95593E-07
RBM10	NVP-BEZ235	-0.199090635	≤0.001
RBM10	1S,3R-RSL-3	-0.198950741	1.91096E-07
RBM10	ML239	-0.198703972	3.95918E-07
RBM10	AZD8055	-0.197684011	3.52128E-07
RBM10	Mdivi-1	-0.197309816	6.9085E-07
RBM10	Bax channel blocker	-0.197245956	1.94768E-06
RBM10	TG-101348	-0.197028886	2.94976E-07
RBM10	purmorphamine	-0.196857221	≤0.001
RBM10	BRD6340	-0.194083665	5.44074E-07
RBM10	AZD1480	-0.19253934	0.002673439
RBM10	AA-COCF3	-0.191548857	2.1863E-06
RBM10	CHIR-99021	-0.191427717	1.57319E-06
RBM10	Compound 1541A	-0.189644137	≤0.001
RBM10	BRD-K29313308	-0.188202814	2.95133E-06
RBM10	skepinone-L	-0.187252105	0.001723457
RBM10	BRD-K45681478	-0.185826651	7.73346E-06
RBM10	BRD-K13999467	-0.185756109	4.00926E-06
RBM10	KU-0063794	-0.18476311	3.06954E-06
RBM10	tosedostat	-0.184021267	5.02735E-06
RBM10	AZD4547	-0.183778212	≤0.001
RBM10	temsirolimus	-0.182980778	0.001554108
RBM10	brefeldin A	-0.182244707	≤0.001
RBM10	Repligen 136	-0.178906543	≤0.001
RBM10	UNC0638	-0.178761994	7.92213E-06
RBM10	RG-108	-0.176611471	≤0.001
RBM10	BRD-K24690302	-0.175894761	≤0.001
RBM10	niclosamide	-0.175747864	≤0.001
RBM10	GSK-3 inhibitor IX	-0.175716111	≤0.001
RBM10	sorafenib	-0.175442933	≤0.001
RBM10	ML162	-0.175355593	7.66196E-06
RBM10	PIK-93	-0.174163402	≤0.001
RBM10	BIRB-796	-0.173542576	≤0.001
RBM10	Ch-55	-0.172383711	≤0.001
RBM10	BMS-754807	-0.171748465	≤0.001
RBM10	AM-580	-0.171640682	≤0.001
RBM10	masitinib	-0.170639389	≤0.001
RBM10	YM-155	-0.169232312	≤0.001
RBM10	BRD-K55116708	-0.169048857	≤0.001
RBM10	cabozantinib	-0.168048046	≤0.001
RBM10	BIBR-1532	-0.167538879	≤0.001
RBM10	BRD-K41597374	-0.167397665	≤0.001

RBM10	NSC 74859	-0.16708525	≤0.001
RBM10	ML050	-0.166146753	≤0.001
RBM10	StemRegenin 1	-0.165654168	≤0.001
RBM10	nintedanib	-0.164863553	≤0.001
RBM10	ruxolitinib	-0.164669487	≤0.001
RBM10	KU-55933	-0.16450581	≤0.001
RBM10	vorapaxar	-0.163857426	≤0.001
RBM10	HBX-41108	-0.163651878	0.001230782
RBM10	lenvatinib	-0.162800029	≤0.001
RBM10	imatinib	-0.162210409	≤0.001
RBM10	BRD1835	-0.161762452	≤0.001
RBM10	CD-1530	-0.159600309	0.001293039
RBM10	OSI-027	-0.159055505	≤0.001
RBM10	ML320	-0.157593522	≤0.001
RBM10	16-beta-bromoandrosterone	-0.157474617	≤0.001
RBM10	tandutinib	-0.156935049	≤0.001
RBM10	BRD-K63431240	-0.155260196	≤0.001
RBM10	CIL41	-0.155094341	0.016264129
RBM10	KHS101	-0.154848542	≤0.001
RBM10	dexamethasone	-0.154786091	≤0.001
RBM10	BRD-A02303741	-0.154778486	0.00160666
RBM10	BRD-K97651142	-0.153649699	≤0.001
RBM10	PDMP	-0.153390551	≤0.001
RBM10	BRD-A71883111	-0.152119933	≤0.001
RBM10	BMS-270394	-0.150767315	≤0.001
RBM10	MK-2206	-0.145088122	≤0.001
RBM10	tivozanib	-0.143574464	0.001107668
RBM10	LE-135	-0.143285588	≤0.001
RBM10	SCH-529074	-0.143136335	0.001076522
RBM10	DBeQ	-0.142643608	≤0.001
RBM10	prochlorperazine	-0.142388718	≤0.001
RBM10	dabrafenib	-0.141939509	0.038402495
RBM10	regorafenib	-0.138054715	0.002741171
RBM10	AZD7545	-0.137551939	≤0.001
RBM10	BMS-195614	-0.135324597	0.01463656
RBM10	MI-2	-0.134245229	0.038203064
RBM10	PF-750	-0.130413345	0.003586307
RBM10	linsitinib	-0.129658684	0.001687375
RBM10	bexarotene	-0.12121199	0.003877024
RBM10	quizartinib	-0.119726534	0.022786462
RBM10	azacitidine	-0.119715477	0.009355954
RBM10	necrostatin-1	-0.118310785	0.007779996
RBM10	parthenolide	-0.117040546	0.022956726

RBM10	GDC-0941	-0.114518624	0.007278228
RBM10	NVP-TAE684	-0.110082204	0.012340032
RBM10	fluvastatin	-0.109922019	0.019473151
RBM10	ML029	-0.109147621	0.010751584
RBM10	NVP-ADW742	-0.1091076	0.010296305
RBM10	necrostatin-7	-0.10777509	0.012860534
RBM10	GSK4112	-0.106935185	0.019130139
RBM10	pyrazolanthrone	-0.10662658	0.018601588
RBM10	RAF265	-0.106535118	0.037516088
RBM10	BRD-K02251932	-0.105994519	0.025816927
RBM10	XL765	-0.103692614	0.042665256
RBM10	ML203	-0.102944103	0.040475716
RBM10	VER-155008	-0.10113511	0.014178719
RBM10	spautin-1	-0.099405965	0.033935819
RBM10	TG-100-115	-0.095478086	0.045094582
RBM10	BRD-K85133207	-0.09528713	0.027044231
RBM10	cytochalasin B	-0.094100992	0.049057695
RBM10	pandacostat	-0.092589108	0.041944338

Table S7 Top 50 RBM10 related genes were explored using GEPIA2.

Gene Symbol	Gene ID	PCC
UBTF	ENSG00000108312.14	0.63
SAFB	ENSG00000160633.12	0.61

DHX30	ENSG00000132153.14	0.6
HNRNPA0	ENSG00000177733.6	0.58
SUGP1	ENSG00000105705.15	0.58
MLLT1	ENSG00000130382.8	0.57
ILF3	ENSG00000129351.17	0.56
HDGFRP2	ENSG00000167674.14	0.56
SRRT	ENSG00000087087.18	0.56
CCDC22	ENSG00000101997.12	0.55
HCFC1	ENSG00000172534.13	0.55
U2AF2	ENSG00000063244.12	0.55
NELFB	ENSG00000188986.4	0.55
UBA1	ENSG00000130985.16	0.54
BRF1	ENSG00000185024.15	0.54
SNRNP70	ENSG00000104852.14	0.54
OTUD5	ENSG00000068308.13	0.54
ZNF777	ENSG00000196453.7	0.54
DDX39B	ENSG00000198563.13	0.54
FUS	ENSG00000089280.18	0.54
CHTOP	ENSG00000160679.12	0.54
GRIPAP1	ENSG00000068400.13	0.53
NCOA5	ENSG00000124160.11	0.53
RANBP3	ENSG00000031823.14	0.53
GPKOW	ENSG00000068394.10	0.53
EHMT2	ENSG00000204371.11	0.53
EWSR1	ENSG00000182944.17	0.53
SF3B2	ENSG00000087365.14	0.53
CACTIN	ENSG00000105298.13	0.53
DHX9	ENSG00000135829.16	0.53
HNRNPM	ENSG00000099783.11	0.53
NRF1	ENSG00000106459.14	0.53
TAF6L	ENSG00000162227.7	0.53
MED22	ENSG00000148297.15	0.52
USP11	ENSG00000102226.9	0.52
SNAPC4	ENSG00000165684.3	0.52
AAAS	ENSG00000094914.12	0.52
GTF2F1	ENSG00000125651.13	0.51
COPS7B	ENSG00000144524.17	0.51
SNRNP200	ENSG00000144028.14	0.51
DPF2	ENSG00000133884.9	0.51
SGTA	ENSG00000104969.9	0.51
CPSF3L	ENSG00000127054.18	0.51
SFSWAP	ENSG00000061936.9	0.51
SART3	ENSG00000075856.11	0.51

PELP1	ENSG00000141456.14	0.51
ALKBH4	ENSG00000160993.3	0.51
C19orf68	ENSG00000185453.12	0.51
CCAR2	ENSG00000158941.16	0.51
NONO	ENSG00000147140.15	0.5

Table S8 The detail enrichment results of KEGG pathway and HALLMARK of RBM10 in GSEA.

Term	ES	NES	NP	FDR	FWER
KEGG_CELL_CYCLE	-0.6771	-2.1334	0	0.0216	0.011

KEGG_DNA_REPLICATION	-0.8265	-2.0059	0	0.0423	0.044
KEGG_MISMATCH_REPAIR	-0.7644	-1.8842	0.0022	0.133	0.157
KEGG_PROTEASOME	-0.7015	-1.7962	0.0129	0.2401	0.307
KEGG_HOMOLOGOUS_RECOMBINATION	-0.6789	-1.7852	0.0106	0.2107	0.326
KEGG_OOCYTE_MEIOSIS	-0.4927	-1.7708	0.0145	0.1997	0.355
KEGG_SPLICEOSOME	-0.5873	-1.7658	0.029	0.1772	0.368
KEGG_BASAL_TRANSCRIPTION_FACTORS	-0.5794	-1.7461	0.0143	0.1866	0.407
KEGG_PENTOSE_PHOSPHATE_PATHWAY	-0.5792	-1.7444	0.0146	0.1679	0.41
KEGG_NUCLEOTIDE_EXCISION_REPAIR	-0.5748	-1.7173	0.023	0.1881	0.467
KEGG_PROGESTERONE_MEDIATED_OOCYTE_MATURATION	-0.4706	-1.6905	0.0123	0.2132	0.539
KEGG_CYSTEINE_AND_METHIONINE_METABOLISM	-0.5071	-1.6039	0.0178	0.3519	0.703
KEGG_N_GLYCAN_BIOSYNTHESIS	-0.4799	-1.5662	0.0615	0.4049	0.762
KEGG_PYRIMIDINE_METABOLISM	-0.4252	-1.5085	0.0801	0.5189	0.835
KEGG_ALZHEIMERS_DISEASE	-0.4249	-1.5049	0.085	0.4933	0.84
KEGG_P53_SIGNALING_PATHWAY	-0.408	-1.5038	0.0568	0.4656	0.843
KEGG_ONE_CARBON_POOL_BY_FOLATE	-0.5457	-1.4747	0.0812	0.5077	0.868
KEGG_AMYOTROPHIC_LATERAL_SCLEROSIS_ALS	-0.3977	-1.4725	0.0566	0.4844	0.868
KEGG_BASE_EXCISION_REPAIR	-0.523	-1.4629	0.1186	0.4825	0.879
KEGG_GLYOXYLATE_AND_DICARBOXYLATE_METABOLISM	-0.5453	-1.4607	0.0925	0.4632	0.881
KEGG_PURINE_METABOLISM	-0.358	-1.4584	0.0701	0.4456	0.882
KEGG_VIBRIO_CHOLERAE_INFECTION	-0.4093	-1.4517	0.0673	0.4397	0.886
KEGG_GLYCOSYLPHOSPHATIDYLINOSITOL_GPI_ANCHOR_BIOSYNTHESIS	-0.4868	-1.4232	0.1266	0.4887	0.911
KEGG_PARKINSONS_DISEASE	-0.4858	-1.4124	0.1785	0.4908	0.919
KEGG_HUNTINGTONS_DISEASE	-0.4052	-1.4072	0.1478	0.4853	0.923
KEGG_AMINOACYL_TRNA_BIOSYNTHESIS	-0.5123	-1.404	0.163	0.4734	0.927
KEGG_FOLATE_BIOSYNTHESIS	-0.5611	-1.3906	0.139	0.4854	0.941
KEGG_RNA_DEGRADATION	-0.4542	-1.3864	0.1747	0.4772	0.944
KEGG_CITRATE_CYCLE_TCA_CYCLE	-0.5233	-1.3798	0.1429	0.4756	0.948
KEGG_GLYCOSPHINGOLIPID_BIOSYNTHESIS_GLOBO_SERIES	-0.4926	-1.3725	0.1148	0.4747	0.951
KEGG_RIBOFLAVIN_METABOLISM	-0.4519	-1.3538	0.1537	0.4956	0.957
KEGG_GAP_JUNCTION	-0.3671	-1.348	0.1201	0.4917	0.96
KEGG_CARDIAC_MUSCLE_CONTRACTION	-0.3768	-1.3393	0.1245	0.4945	0.965
KEGG_UBIQUITIN_MEDIATED_PROTEOLYSIS	-0.3781	-1.3314	0.1898	0.4963	0.969
KEGG_GLYCOSAMINOGLYCAN_BIOSYNTHESIS_HEPARAN_SULFATE	-0.42	-1.3248	0.1515	0.4949	0.971
KEGG_LYSINE_DEGRADATION	-0.3998	-1.3028	0.1726	0.5282	0.98
KEGG_OXIDATIVE_PHOSPHORYLATION	-0.4525	-1.302	0.2645	0.5155	0.981
KEGG_NON_HOMOLOGOUS_END_JOINING	-0.5151	-1.2901	0.1947	0.5251	0.987
KEGG_GLUTATHIONE_METABOLISM	-0.3952	-1.2878	0.1804	0.516	0.987
KEGG_PATHOGENIC_ESCHERICHIA_COLI_INFECTION	-0.3853	-1.2846	0.2008	0.5094	0.988
KEGG_DORSO_VENTRAL_AXIS_FORMATION	-0.4217	-1.2824	0.1703	0.5009	0.988

KEGG_GALACTOSE_METABOLISM	-0.4034	-1.2819	0.1623	0.4898	0.988
KEGG_RNA_POLYMERASE	-0.4328	-1.2722	0.2114	0.4965	0.991
KEGG_REGULATION_OF_AUTOPHAGY	-0.3774	-1.2508	0.1677	0.5259	0.993
KEGG_SMALL_CELL_LUNG_CANCER	-0.3516	-1.2463	0.2037	0.5225	0.993
KEGG_EPITHELIAL_CELL_SIGNALING_IN_HELICOBACTER_ PYLORI_INFECTION	-0.3426	-1.2441	0.1967	0.5154	0.993
KEGG_PANCREATIC_CANCER	-0.3443	-1.2092	0.2474	0.5715	0.994
KEGG_BLADDER_CANCER	-0.3386	-1.1937	0.2417	0.5921	0.995
KEGG_PATHWAYS_IN_CANCER	-0.2878	-1.1929	0.2276	0.5817	0.995
KEGG_GLYCOLYSIS_GLUCONEOGENESIS	-0.3417	-1.1923	0.2464	0.5711	0.995
KEGG_RENAL_CELL_CARCINOMA	-0.3367	-1.1905	0.2567	0.5635	0.996
KEGG_BIOSYNTHESIS_OF_UNSATURATED_FATTY_ACIDS	-0.3948	-1.1705	0.2868	0.5919	0.997
KEGG_GLYCINE_SERINE_AND_THREONINE_METABOLISM	-0.3728	-1.163	0.2524	0.5954	0.997
KEGG_ENDOCYTOSIS	-0.2955	-1.1547	0.278	0.6017	0.998
KEGG_ALANINE_ASPARTATE_AND_GLUTAMATE_METABOLI SM	-0.3427	-1.1449	0.2791	0.6095	0.998
KEGG_PHENYLALANINE_METABOLISM	-0.3958	-1.1308	0.3159	0.6254	0.998
KEGG_NOTCH_SIGNALING_PATHWAY	-0.3443	-1.1268	0.3366	0.6225	0.998
KEGG_SULFUR_METABOLISM	-0.4295	-1.1255	0.3145	0.6142	0.998
KEGG_PROTEIN_EXPORT	-0.4101	-1.1169	0.3497	0.6213	0.998
KEGG_FC_GAMMA_R_MEDIATED_PHAGOCYTOSIS	-0.3061	-1.1144	0.334	0.6158	0.998
KEGG_NEUROACTIVE_LIGAND_RECEPTOR_INTERACTION	-0.2605	-1.1127	0.264	0.6091	0.998
KEGG_PRION_DISEASES	-0.3407	-1.0983	0.334	0.6257	0.998
KEGG_PANTOTHENATE_AND_COA_BIOSYNTHESIS	-0.3611	-1.0917	0.3434	0.6288	0.998
KEGG_PROSTATE_CANCER	-0.2984	-1.0912	0.3307	0.62	0.998
KEGG_GLIOMA	-0.3002	-1.0894	0.342	0.6137	0.998
KEGG_NON_SMALL_CELL_LUNG_CANCER	-0.3171	-1.087	0.3691	0.6087	0.998
KEGG_PYRUVATE_METABOLISM	-0.3346	-1.0653	0.3765	0.6411	0.999
KEGG_REGULATION_OF_ACTIN_CYTOSKELETON	-0.268	-1.0502	0.3596	0.6599	0.999
KEGG_MELANOMA	-0.282	-1.0492	0.3931	0.6523	0.999
KEGG_SELENOAMINO_ACID_METABOLISM	-0.3353	-1.043	0.4114	0.6545	0.999
KEGG_VALINE_LEUCINE_AND_ISOLEUCINE_BIOSYNTHESIS	-0.4442	-1.0419	0.4346	0.6475	0.999
KEGG_PRIMARY_IMMUNODEFICIENCY	-0.3881	-1.027	0.4433	0.6657	1
KEGG_LONG_TERM_DEPRESSION	-0.2573	-1.0243	0.4129	0.6615	1
KEGG_ADIPOCYTOKINE_SIGNALING_PATHWAY	-0.2759	-1.0088	0.4571	0.6827	1
KEGG_TIGHT_JUNCTION	-0.252	-0.9831	0.4513	0.723	1
KEGG_OTHER_GLYCAN_DEGRADATION	-0.3713	-0.9818	0.4672	0.7164	1
KEGG_CHEMOKINE_SIGNALING_PATHWAY	-0.2571	-0.9736	0.4825	0.7239	1
KEGG_CHRONIC_MYELOID_LEUKEMIA	-0.28	-0.971	0.4909	0.7203	1
KEGG_MELANOGENESIS	-0.2419	-0.9634	0.5209	0.7254	1
KEGG_FRUCTOSE_AND_MANNOSE_METABOLISM	-0.2867	-0.9609	0.5	0.721	1
KEGG_ACUTE_MYELOID_LEUKEMIA	-0.2673	-0.9347	0.5184	0.7621	1
KEGG_GLYCOSPHINGOLIPID_BIOSYNTHESIS_LACTO_AND_	-0.2778	-0.9051	0.5678	0.8099	1

NEOLACTO_SERIES					
KEGG_INOSITOL_PHOSPHATE_METABOLISM	-0.2616	-0.8983	0.5513	0.8142	1
KEGG_GLYCEROLIPID_METABOLISM	-0.2404	-0.8963	0.6052	0.8079	1
KEGG_ARGININE_AND_PROLINE_METABOLISM	-0.2555	-0.8951	0.5922	0.8012	1
KEGG_ADHERENS_JUNCTION	-0.2594	-0.893	0.5638	0.7966	1
KEGG_CALCIUM_SIGNALING_PATHWAY	-0.2071	-0.8768	0.6762	0.8183	1
KEGG_AMINO_SUGAR_AND_NUCLEOTIDE_SUGAR_METABOLISM	-0.2632	-0.875	0.5876	0.8126	1
KEGG_NOD_LIKE_RECEPTOR_SIGNALING_PATHWAY	-0.2538	-0.8711	0.5963	0.8116	1
KEGG_CYTOSOLIC_DNA_SENSING_PATHWAY	-0.2393	-0.8702	0.6159	0.804	1
KEGG_GLYCOSAMINOGLYCAN_BIOSYNTHESIS_KERATAN_SULFATE	-0.3146	-0.8698	0.6182	0.7962	1
KEGG_TOLL_LIKE_RECEPTOR_SIGNALING_PATHWAY	-0.2352	-0.8613	0.5864	0.8043	1
KEGG_TERPENOID_BACKBONE_BIOSYNTHESIS	-0.3353	-0.8586	0.6067	0.8012	1
KEGG_DRUG_METABOLISM_OTHER_ENZYMES	-0.2437	-0.8469	0.7	0.8147	1
KEGG_COLORECTAL_CANCER	-0.2368	-0.8298	0.6513	0.8395	1
KEGG_LEUKOCYTE_TRANSENDOTHELIAL_MIGRATION	-0.2209	-0.811	0.6918	0.8665	1
KEGG_NICOTINATE_AND_NICOTINAMIDE_METABOLISM	-0.2462	-0.8086	0.7303	0.862	1
KEGG_MAPK_SIGNALING_PATHWAY	-0.1882	-0.8074	0.7465	0.8555	1
KEGG_LYSOSOME	-0.2355	-0.8071	0.6446	0.8474	1
KEGG_INSULIN_SIGNALING_PATHWAY	-0.1981	-0.7941	0.7631	0.8613	1
KEGG_SPHINGOLIPID_METABOLISM	-0.2332	-0.7827	0.7174	0.8728	1
KEGG_GLYCOSAMINOGLYCAN_BIOSYNTHESIS_CHONDROITIN_SULFATE	-0.2637	-0.766	0.7423	0.8931	1
KEGG_B_CELL_RECEPTOR_SIGNALING_PATHWAY	-0.2254	-0.7609	0.714	0.8931	1
KEGG_NEUROTROPHIN_SIGNALING_PATHWAY	-0.2026	-0.7518	0.7646	0.8998	1
KEGG_WNT_SIGNALING_PATHWAY	-0.1878	-0.74	0.8306	0.9108	1
KEGG_PHOSPHATIDYLINOSITOL_SIGNALING_SYSTEM	-0.2061	-0.7357	0.7813	0.9087	1
KEGG_THYROID_CANCER	-0.2379	-0.7335	0.7946	0.9046	1
KEGG_T_CELL_RECEPTOR_SIGNALING_PATHWAY	-0.2095	-0.7332	0.7369	0.8967	1
KEGG_ERBB_SIGNALING_PATHWAY	-0.1858	-0.6984	0.8436	0.9409	1
KEGG_PORPHYRIN_AND_CHLOROPHYLL_METABOLISM	-0.2124	-0.6955	0.8706	0.9363	1
KEGG_OLFACTORY_TRANSDUCTION	-0.2266	-0.6904	0.8665	0.9349	1
KEGG_RIG_I_LIKE_RECEPTOR_SIGNALING_PATHWAY	-0.183	-0.6778	0.8994	0.9441	1
KEGG_TGF_BETA_SIGNALING_PATHWAY	-0.1853	-0.6752	0.8928	0.9388	1
KEGG_SYSTEMIC_LUPUS_ERYTHEMATOSUS	-0.204	-0.6742	0.7725	0.932	1
KEGG_LONG_TERM_POTENTIATION	-0.1787	-0.6709	0.9234	0.9278	1
KEGG_ANTIGEN_PROCESSING_AND_PRESENTATION	-0.2047	-0.6687	0.8182	0.9227	1
KEGG_AXON_GUIDANCE	-0.1713	-0.6655	0.9245	0.9186	1
KEGG_NATURAL_KILLER_CELL_MEDIATED_CYTOTOXICITY	-0.1784	-0.6649	0.872	0.9116	1
KEGG_ENDOMETRIAL_CANCER	-0.194	-0.6583	0.877	0.9114	1
KEGG_STARCH_AND_SUCROSE_METABOLISM	-0.1883	-0.6494	0.958	0.9141	1
KEGG_VIRAL_MYOCARDITIS	-0.1957	-0.6344	0.8407	0.9237	1

KEGG_PENTOSE_AND_GLUCURONATE_INTERCONVERSIONS	-0.2211	-0.6336	0.9165	0.917	1
KEGG_STEROID_HORMONE_BIOSYNTHESIS	-0.2047	-0.6282	0.9715	0.9146	1
KEGG_STEROID_BIOSYNTHESIS	-0.2421	-0.6184	0.8651	0.9164	1
KEGG_FOCAL_ADHESION	-0.1339	-0.4713	0.9979	0.9908	1
KEGG_RETINOL_METABOLISM	-0.1354	-0.45	1	0.9874	1
KEGG_METABOLISM_OF_XENOBIOTICS_BY_CYTOCHROME_P450	0.1482	0.4796	0.9961	0.9805	1
KEGG_MATURITY_ONSET_DIABETES_OF_THE_YOUNG	0.177	0.5051	0.9921	0.9878	1
KEGG_MTOR_SIGNALING_PATHWAY	0.1457	0.5196	0.9941	0.9989	1
KEGG_TASTE_TRANSDUCTION	0.1715	0.5281	0.9627	1	1
KEGG_LEISHMANIA_INFECTION	0.1828	0.5505	0.8887	1	1
KEGG_ECM_RECEPTOR_INTERACTION	0.174	0.5527	0.9348	1	1
KEGG_CYTOKINE_CYTOKINE_RECEPTOR_INTERACTION	0.1514	0.5802	0.9466	1	1
KEGG_BASAL_CELL_CARCINOMA	0.1753	0.618	0.952	1	1
KEGG_TYPE_I_DIABETES_MELLITUS	0.2361	0.6355	0.8069	1	1
KEGG_DRUG_METABOLISM_CYTOCHROME_P450	0.1945	0.6408	0.9453	1	1
KEGG_ASCORBATE_AND_ALDARATE_METABOLISM	0.2404	0.6509	0.9022	1	1
KEGG_JAK_STAT_SIGNALING_PATHWAY	0.1779	0.6842	0.8842	1	1
KEGG_DILATED_CARDIOMYOPATHY	0.1889	0.6897	0.8974	1	1
KEGG_TAURINE_AND_HYPOTAURINE_METABOLISM	0.2816	0.7275	0.8396	0.9907	1
KEGG_GNRH_SIGNALING_PATHWAY	0.1819	0.729	0.8784	1	1
KEGG_CELL_ADHESION_MOLECULES_CAMS	0.2082	0.7299	0.7296	1	1
KEGG_HYPERTROPHIC_CARDIOMYOPATHY_HCM	0.2036	0.7473	0.8242	1	1
KEGG_TRYPTOPHAN_METABOLISM	0.224	0.7475	0.8087	1	1
KEGG_HEDGEHOG_SIGNALING_PATHWAY	0.2074	0.7567	0.8058	1	1
KEGG_COMPLEMENT_AND_COAGULATION_CASCADES	0.2324	0.7659	0.7365	1	1
KEGG_VEGF_SIGNALING_PATHWAY	0.1962	0.7684	0.8116	1	1
KEGG_GLYCOSAMINOGLYCAN_DEGRADATION	0.2671	0.7721	0.72	1	1
KEGG_TYROSINE_METABOLISM	0.2251	0.7784	0.8157	1	1
KEGG_GRAFT_VERSUS_HOST_DISEASE	0.3202	0.7802	0.6699	1	1
KEGG_ARRHYTHMOGENIC_RIGHT_VENTRICULAR_CARDIOMYOPATHY_ARVC	0.2201	0.7804	0.757	1	1
KEGG_BETA_ALANINE_METABOLISM	0.2494	0.7893	0.7568	1	1
KEGG_SNARE_INTERACTIONS_IN_VESICULAR_TRANSPORT	0.2413	0.7994	0.7126	1	1
KEGG_GLYCEROPHOSPHOLIPID_METABOLISM	0.2085	0.8157	0.7405	1	1
KEGG_RENIN_ANGIOTENSIN_SYSTEM	0.2982	0.8504	0.6772	1	1
KEGG_VASOPRESSIN_REGULATED_WATER_REABSORPTION	0.2624	0.8613	0.656	1	1
KEGG_TYPE_II_DIABETES_MELLITUS	0.2476	0.8791	0.6494	1	1
KEGG_ABC_TRANSPORTERS	0.2563	0.91	0.5758	1	1
KEGG_APOPTOSIS	0.2598	0.9372	0.5262	1	1
KEGG_VASCULAR_SMOOTH_MUSCLE_CONTRACTION	0.2402	0.9457	0.5287	1	1
KEGG_ETHER_LIPID_METABOLISM	0.2791	0.968	0.5073	1	1

KEGG_AUTOIMMUNE_THYROID_DISEASE	0.3437	0.9705	0.4795	1	1
KEGG_HEMATOPOIETIC_CELL_LINEAGE	0.3122	0.9972	0.4482	1	1
KEGG_PPAR_SIGNALING_PATHWAY	0.2695	1.0095	0.4185	1	1
KEGG_INTESTINAL_IMMUNE_NETWORK_FOR_IGA_PRODUCTION	0.3806	1.0115	0.4691	1	1
KEGG_FC_EPSILON_RI_SIGNALING_PATHWAY	0.2769	1.035	0.4274	1	1
KEGG_ALLOGRAFT_REJECTION	0.4411	1.065	0.4453	1	1
KEGG_VALINE_LEUCINE_AND_ISOLEUCINE_DEGRADATION	0.3551	1.0659	0.3938	1	1
KEGG_BUTANOATE_METABOLISM	0.3394	1.0736	0.3775	1	1
KEGG_RIBOSOME	0.5229	1.0938	0.4415	1	0.998
KEGG_CIRCADIAN_RHYTHM_MAMMAL	0.4284	1.126	0.326	1	0.998
KEGG_PROPANOATE_METABOLISM	0.383	1.1366	0.3306	1	0.998
KEGG_PROXIMAL_TUBULE_BICARBONATE_RECLAMATION	0.3631	1.1458	0.2886	1	0.998
KEGG_HISTIDINE_METABOLISM	0.3683	1.1857	0.2598	1	0.994
KEGG_PRIMARY_BILE_ACID_BIOSYNTHESIS	0.424	1.1866	0.2358	1	0.994
KEGG_PEROXISOME	0.34	1.1881	0.2649	1	0.994
KEGG_LIMONENE_AND_PINENE_DEGRADATION	0.4976	1.1981	0.2651	1	0.994
KEGG_O_GLYCAN_BIOSYNTHESIS	0.4079	1.2585	0.1976	1	0.989
KEGG_FATTY_ACID_METABOLISM	0.3952	1.2616	0.1996	1	0.988
KEGG_LINOLEIC_ACID_METABOLISM	0.4127	1.2632	0.1562	1	0.988
KEGG_NITROGEN_METABOLISM	0.4024	1.3163	0.1139	1	0.984
KEGG_ALDOSTERONE_REGULATED_SODIUM_REABSORPTION	0.3813	1.3476	0.1161	1	0.971
KEGG_ALPHA_LINOLENIC_ACID_METABOLISM	0.4997	1.5166	0.0572	0.8195	0.814
KEGG_ASTHMA	0.6228	1.5631	0.1086	0.816	0.732
KEGG_GLYCOSPHINGOLIPID_BIOSYNTHESIS_GANGLIO_SERIES	0.563	1.5649	0.0364	1	0.73
KEGG_ARACHIDONIC_ACID_METABOLISM	0.4391	1.6054	0.0101	1	0.669
HALLMARK_SPERMATOGENESIS	-0.5399	-2.0916	0	0.0272	0.019
HALLMARK_E2F_TARGETS	-0.7373	-1.9891	0	0.0299	0.041
HALLMARK_G2M_CHECKPOINT	-0.7079	-1.9615	0	0.0249	0.053
HALLMARK_MTORC1_SIGNALING	-0.5803	-1.9552	0.0041	0.0199	0.054
HALLMARK_MYC_TARGETS_V1	-0.6282	-1.7232	0.0481	0.0962	0.219
HALLMARK_MYC_TARGETS_V2	-0.6597	-1.6994	0.0367	0.0917	0.241
HALLMARK_UNFOLDED_PROTEIN_RESPONSE	-0.4893	-1.6614	0.037	0.1009	0.296
HALLMARK_PI3K_AKT_MTOR_SIGNALING	-0.4461	-1.6512	0.0381	0.0935	0.308
HALLMARK_DNA_REPAIR	-0.4797	-1.626	0.0644	0.0963	0.333
HALLMARK_MITOTIC_SPINDLE	-0.4903	-1.607	0.0573	0.0983	0.36
HALLMARK_GLYCOLYSIS	-0.403	-1.5072	0.071	0.1518	0.48
HALLMARK_UV_RESPONSE_UP	-0.3432	-1.3706	0.0956	0.2548	0.646
HALLMARK_OXIDATIVE_PHOSPHORYLATION	-0.4654	-1.3681	0.2025	0.2376	0.649
HALLMARK_ESTROGEN_RESPONSE_LATE	-0.2987	-1.2652	0.1437	0.3292	0.753
HALLMARK_HYPOXIA	-0.3037	-1.194	0.2327	0.3942	0.813

HALLMARK_APICAL_SURFACE	-0.3217	-1.144	0.2639	0.4378	0.855
HALLMARK_CHOLESTEROL_HOMEOSTASIS	-0.3246	-1.1229	0.3156	0.4418	0.873
HALLMARK_ANDROGEN_RESPONSE	-0.2762	-1.0189	0.4271	0.572	0.923
HALLMARK_PROTEIN_SECRETION	-0.3019	-0.9947	0.4361	0.5782	0.931
HALLMARK_WNT_BETA_CATENIN_SIGNALING	-0.3161	-0.987	0.462	0.5608	0.934
HALLMARK_XENOBIOTIC_METABOLISM	-0.239	-0.9794	0.4664	0.5463	0.935
HALLMARK_KRAS_SIGNALING_DN	-0.2222	-0.9605	0.539	0.5496	0.94
HALLMARK_REACTIVE_OXYGEN_SPECIES_PATHWAY	-0.2863	-0.8869	0.5794	0.6412	0.959
HALLMARK_COMPLEMENT	-0.218	-0.85	0.6327	0.6763	0.969
HALLMARK_APICAL_JUNCTION	-0.2132	-0.7869	0.7235	0.7583	0.982
HALLMARK_ADIPOGENESIS	-0.2037	-0.7734	0.7275	0.7541	0.985
HALLMARK_APOPTOSIS	-0.192	-0.7624	0.7881	0.7442	0.986
HALLMARK_PANCREAS_BETA_CELLS	-0.2231	-0.7208	0.8504	0.778	0.991
HALLMARK_ALLOGRAFT_REJECTION	-0.152	-0.4981	0.948	0.9626	0.998
HALLMARK_EPITHELIAL_MESENCHYMAL_TRANSITION	0.1354	0.4112	0.9844	0.9864	0.999
HALLMARK_TNFA_SIGNALING_VIA_NFKB	0.1343	0.4633	0.9882	1	0.999
HALLMARK_INTERFERON_ALPHA_RESPONSE	0.1783	0.5004	0.878	1	0.999
HALLMARK_INTERFERON_GAMMA_RESPONSE	0.1659	0.5144	0.9004	1	0.999
HALLMARK_INFLAMMATORY_RESPONSE	0.1556	0.5586	0.9298	1	0.998
HALLMARK_ANGIOGENESIS	0.1729	0.5623	0.9733	1	0.998
HALLMARK_MYOGENESIS	0.1434	0.5694	0.9901	1	0.998
HALLMARK_IL6_JAK_STAT3_SIGNALING	0.1825	0.5928	0.8887	1	0.997
HALLMARK_NOTCH_SIGNALING	0.1935	0.6313	0.9273	1	0.996
HALLMARK_TGF_BETA_SIGNALING	0.1961	0.6394	0.8757	1	0.996
HALLMARK_KRAS_SIGNALING_UP	0.1728	0.7151	0.833	1	0.988
HALLMARK_IL2_STAT5_SIGNALING	0.1874	0.7691	0.748	1	0.986
HALLMARK_BILE_ACID_METABOLISM	0.2227	0.9007	0.5898	1	0.97
HALLMARK_ESTROGEN_RESPONSE_EARLY	0.2137	0.9038	0.5734	1	0.97
HALLMARK_UV_RESPONSE_DN	0.2594	0.9377	0.5094	1	0.961
HALLMARK_FATTY_ACID_METABOLISM	0.2534	0.9418	0.504	1	0.959
HALLMARK_PEROXISOME	0.2509	0.966	0.4817	1	0.948
HALLMARK_HEME_METABOLISM	0.2376	1.01	0.4035	1	0.934
HALLMARK_COAGULATION	0.2752	1.029	0.4048	1	0.926
HALLMARK_HEDGEHOG_SIGNALING	0.3205	1.0594	0.3895	1	0.914
HALLMARK_P53_PATHWAY	0.3405	1.3784	0.1004	1	0.672
