# Supplementary material for: RBM10 Is a Biomarker Associated with Pan-Cancer Prognosis and Immune Infiltration: System Analysis Combined with In Vitro and Vivo Experiments
Source: Oxid Med Cell Longev. 2022 Nov 28;2022:7654937. doi: 10.1155/2022/7654937 (PMC11401663; doi:10.1155/2022/7654937)
Supplement: Supplementary Materials — Figure S1: RBM10 mRNA expression in human normal tissues and different cancer cells. (A) RBM10 mRNA expression in various normal tissues was analyzed using the HPA/GTEx/FANTOM5 database. (B) The mRNA expression level of RBM10 in various cancer cells was analyzed from the data of the CCLE database. Figure S2: the protein expression of RBM10 in pan-cancer and a single-gene GO analysis of RBM10. (A) RBM10 protein level in different tumors and normal adjacent tissues through the CPTAC database. (B) SangerBox showed the positively association between RBM10 expression and each analysis result. Figure S3: the correlation between RBM10 and different molecular subtypes in pan-cancer. (A) The expression of RBM10 was significantly associated with molecular subtypes in BRCA, HNSC, KIRP, LGG, READ, OV, LUSC, and PRAD. (B) The RBM10 expression was not associated with molecular subtypes in ACC, COAD, ESCA, GBM, LIHC, PCPG, and SKCM. (C) The expression level of RBM10 was not associated with tumor stages in BLCA, BRCA, CESC, CHOL, COAD, DLBC, ESCA, HNSC, KIRC, KIRP, LUAD, LUSC, READ, STAD, TGCT, THCA, and UCEC. Figure S4: the prognosis analysis of RBM10 in tumors. (A, B) The correlation analysis of RBM10 expression with overall survival (OS, (A)) and relapse-free survival (RFS, (B)) in patients with different cancer types from the TCGA database of the Kaplan-Meier plotter. The median RBM10 expression was used to classify patients into high expression and low expression groups. p < 0.05 was statistically significant. Figure S5: (A–D) forest map showed the univariate Cox regression results of RBM10 for OS (A), DSS (B), DFI (C), and PFI (D) in multiple cancers. Figure S6: the expression level of RBM10 in the patients with RBM10 wild type (RBM10 WT) or RBM10 mutation (RBM10 Mut) of LUAD, BLCA, and PAAD. Figure S7: the correlation of RBM10 expression with CNV. (A) The association between RBM10 mRNA expression and CNV in different cancers using GSCA. Blue bubbles represented negative corr [file 7654937.f1.zip › Supplementary Figures.pdf]

Figures S1~S13

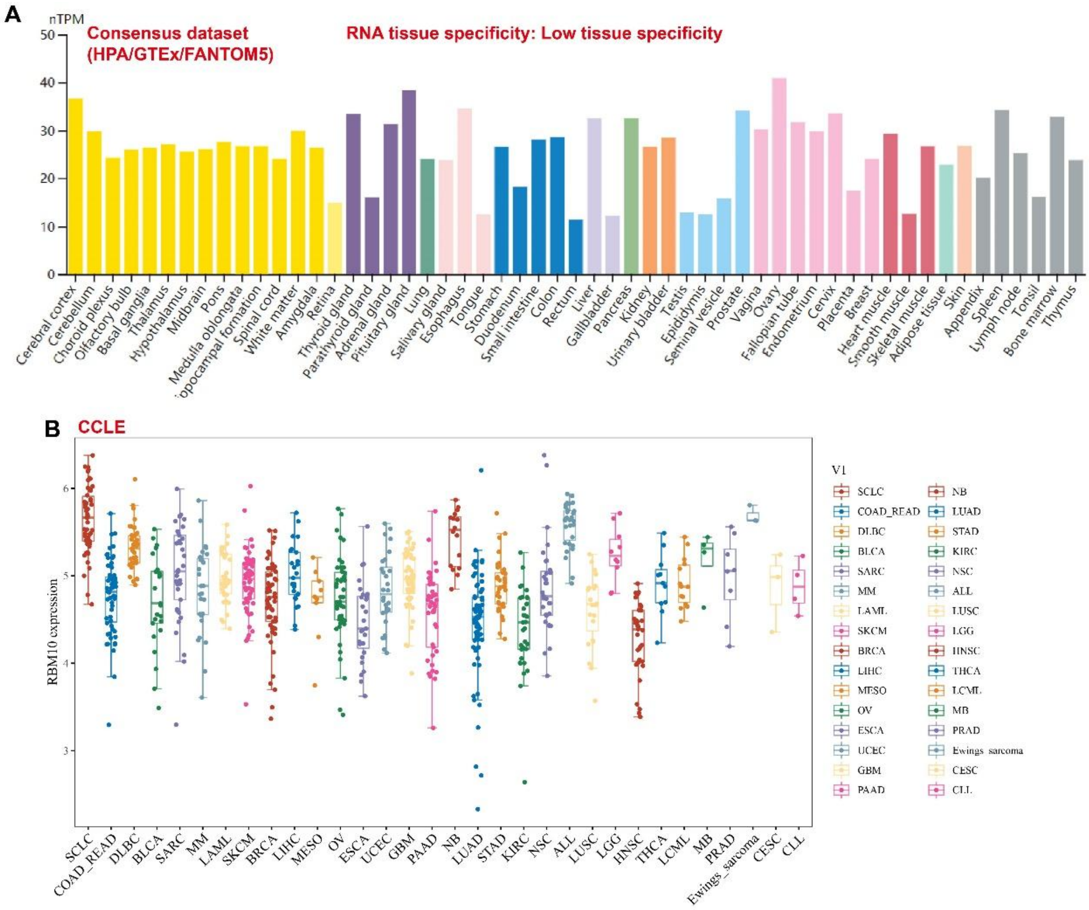

Figure S1 RBM10 mRNA expression in human normal tissues and different cancer cells (A) RBM10 mRNA expression in various normal tissues was analyzed using HPA/GTEx/FANTOM5 database**. (B)** The mRNA expression level of RBM10 in various cancer cells was analyzed from the data of CCLE database.

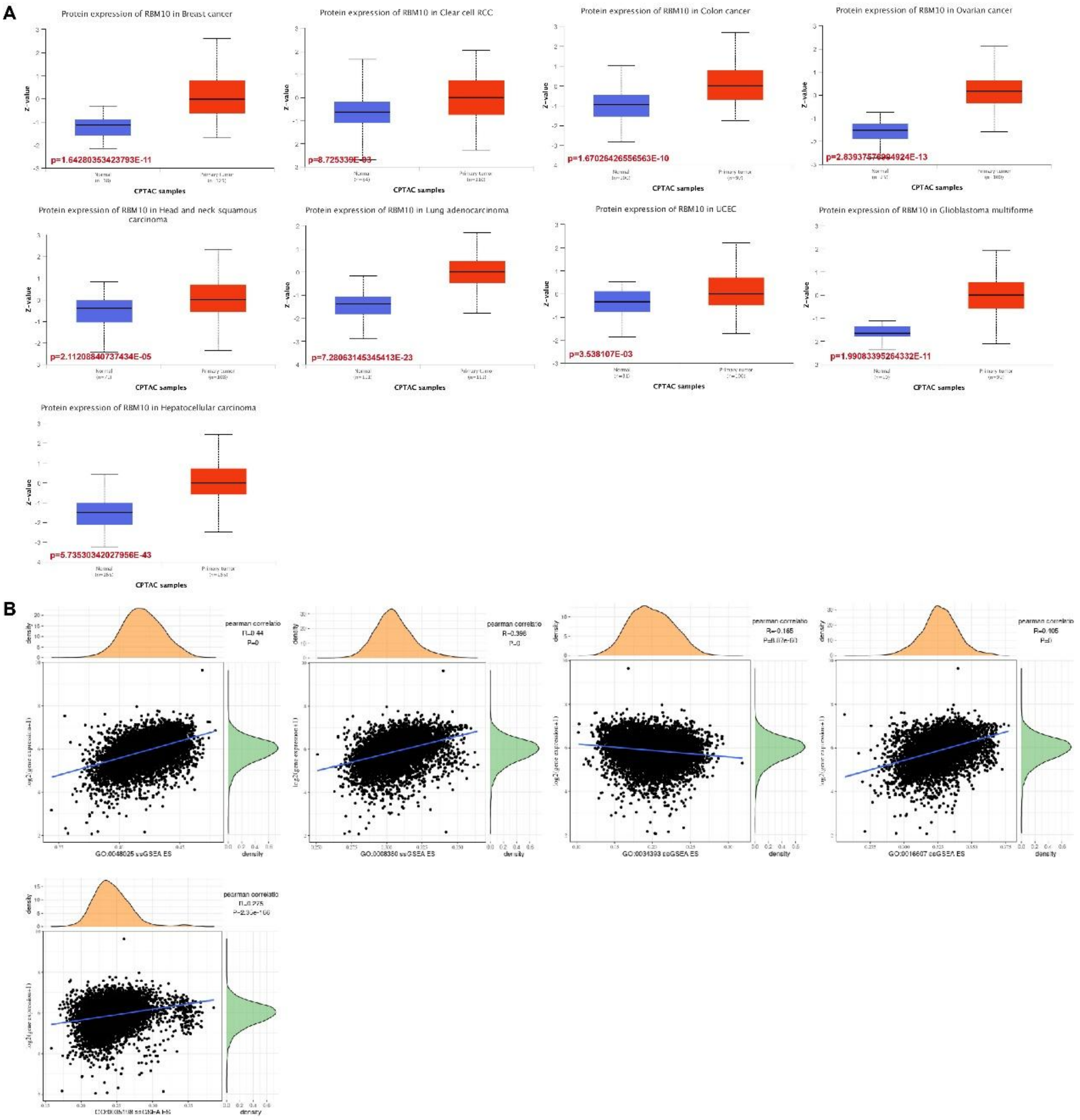

Figure S2 The protein expression of RBM10 in pan-cancer and a single-gene GO analysis of RBM10 (A) RBM10 protein level in different tumors and normal adjacent tissues through CPTAC database. **(B)** SangerBox showed the positively association between RBM10 expression and each analysis result.

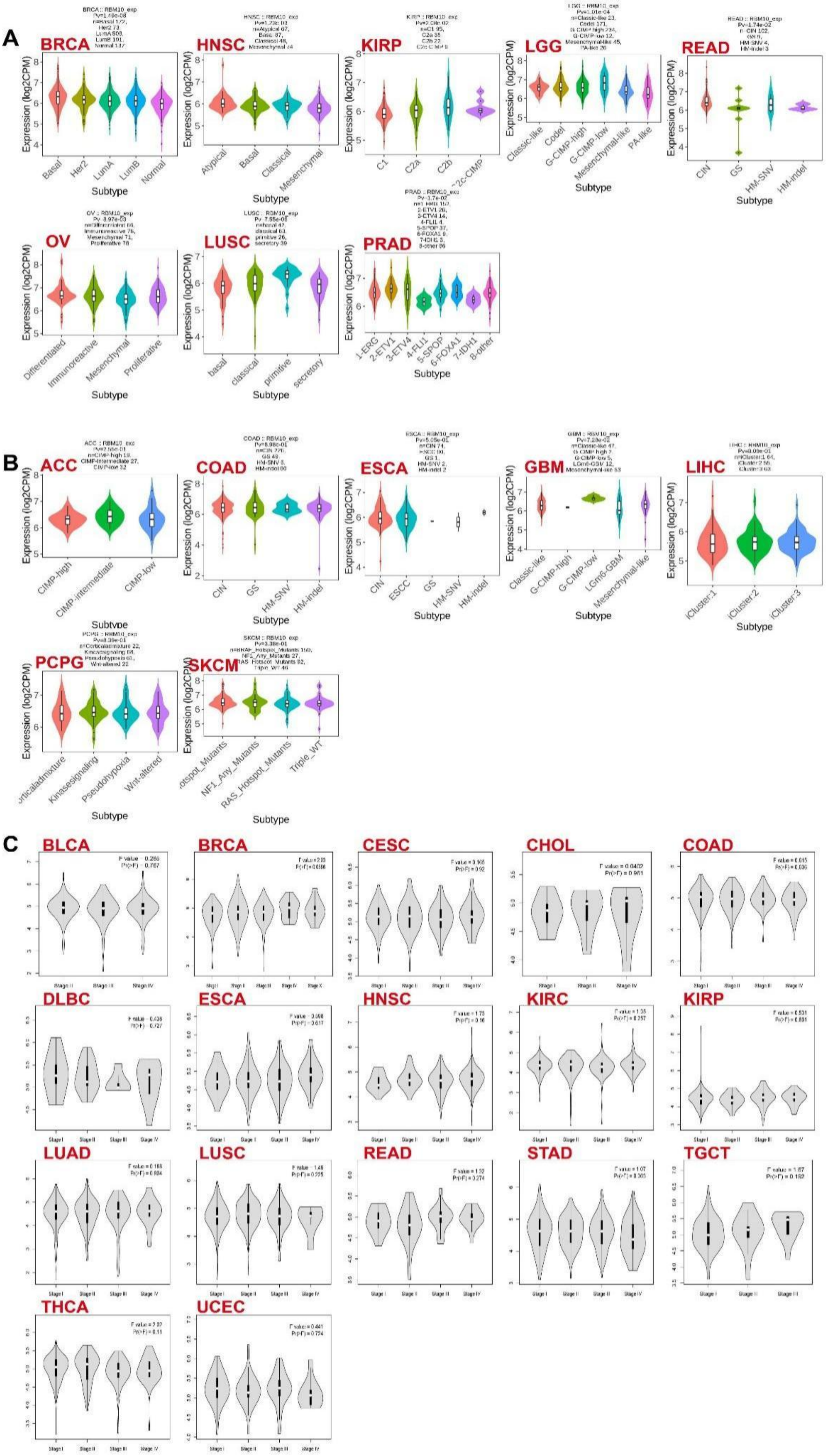
A
BRCA
HNSC
KIRP
LGG
READ
OV
LUSC
PRAD
Expression (log2CPM)
Subtype
Basal
Her2
LumA
LumB
Normal
Atypical
Classical
Mesenchymal
C1
C2a
C2b
Classic-like
Codel
G-CIMP-high
G-CIMP-low
Mesenchymal-like
PA-like
CIN
GS
HM-SNV
HM-indel
Differentiated
Immunoreactive
Proliferative
basal
classical
primitive
secretory
1-ERG
2-ETV1
3-ETV4
4-FLI1
5-SPOP
6-FOXA1
7-IDH1
8-other
B
ACC
COAD
ESCA
GBM
LIHC
PCPG
SKCM
CIMP-high
CIMP-intermediate
CIMP-low
ESCC
LGm6-GBM
iCluster:1
iCluster:2
iCluster:3
Kinasesignaling
Pseudohypoxia
Wnt-altered
NF1_Any_Mutants
RAS_Hotspot_Mutants
Triple_WT
C
BLCA
BRCA
CESC
CHOL
COAD
DLBC
ESCA
HNSC
KIRC
KIRP
LUAD
LUSC
READ
STAD
TGCT
THCA
UCEC

Figure S3 The correlation between RBM10 and different molecular subtypes in pan-cancer (A) The expression of RBM10 was significantly associated with molecular subtypes in BRCA, HNSC, KIRP, LGG, READ, OV, LUSC and PRAD. (**B**) RBM10 expression was no associated with molecular subtypes in ACC, COAD, ESCA, GBM, LIHC, PCPG and SKCM. (**C**) The expression level of RBM10 was no associated with tumor stages in BLCA, BRCA, CESC, CHOL, COAD, DLBC, ESCA, HNSC, KIRC, KIRP, LUAD, LUSC, READ, STAD, TGCT, THCA and UCEC.

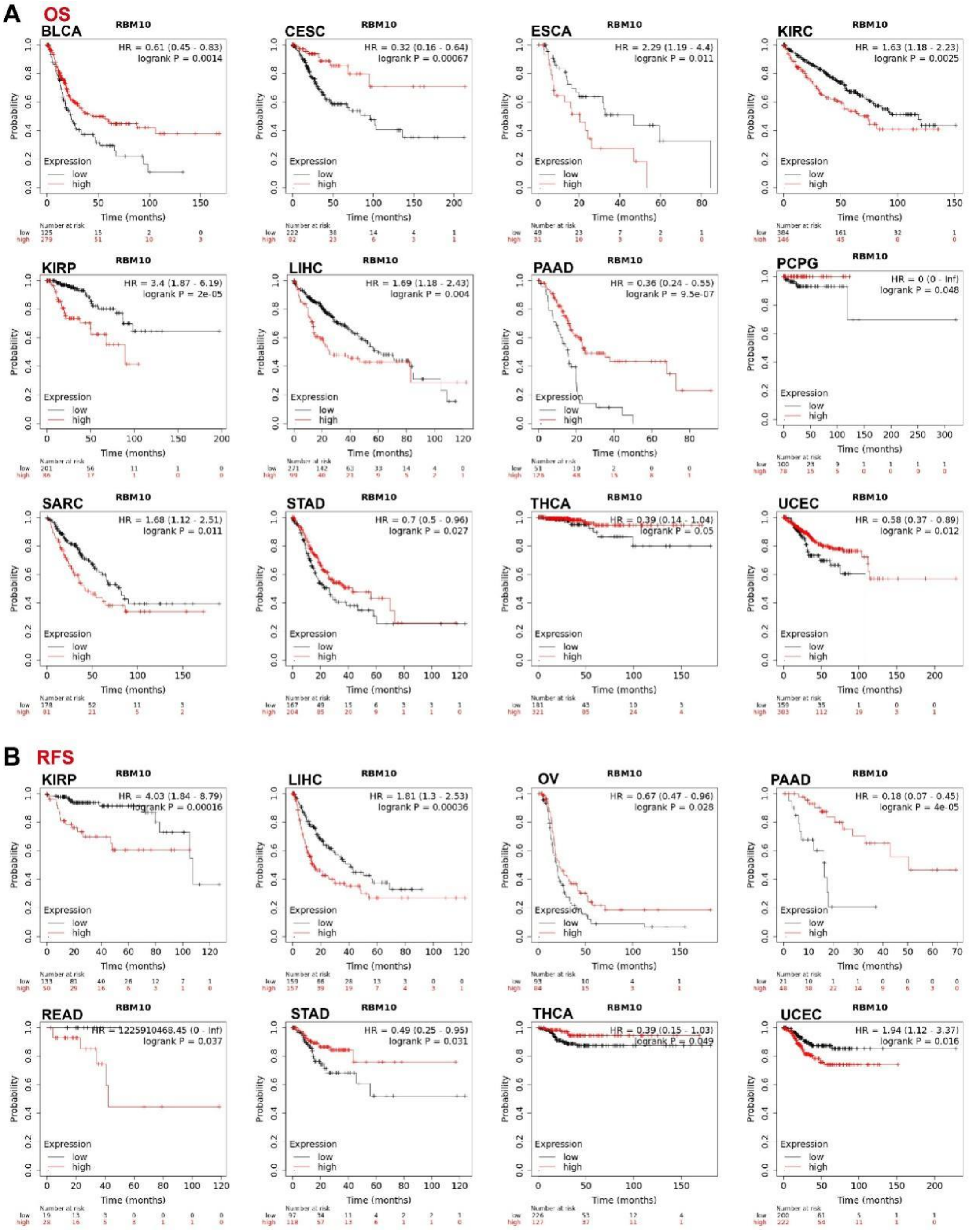

Figure S4 The prognosis analysis of RBM10 in tumors. (A~B) The correlation analysis of RBM10 expression with overall survival (OS, **A**) and relapse free survival (RFS, **B**) in patients with different cancer types from TCGA database of Kaplan-Meier plotter. The median RBM10 expression was used to classify patients into high expression and low expression groups. $p<0.05$ was statistically significant.

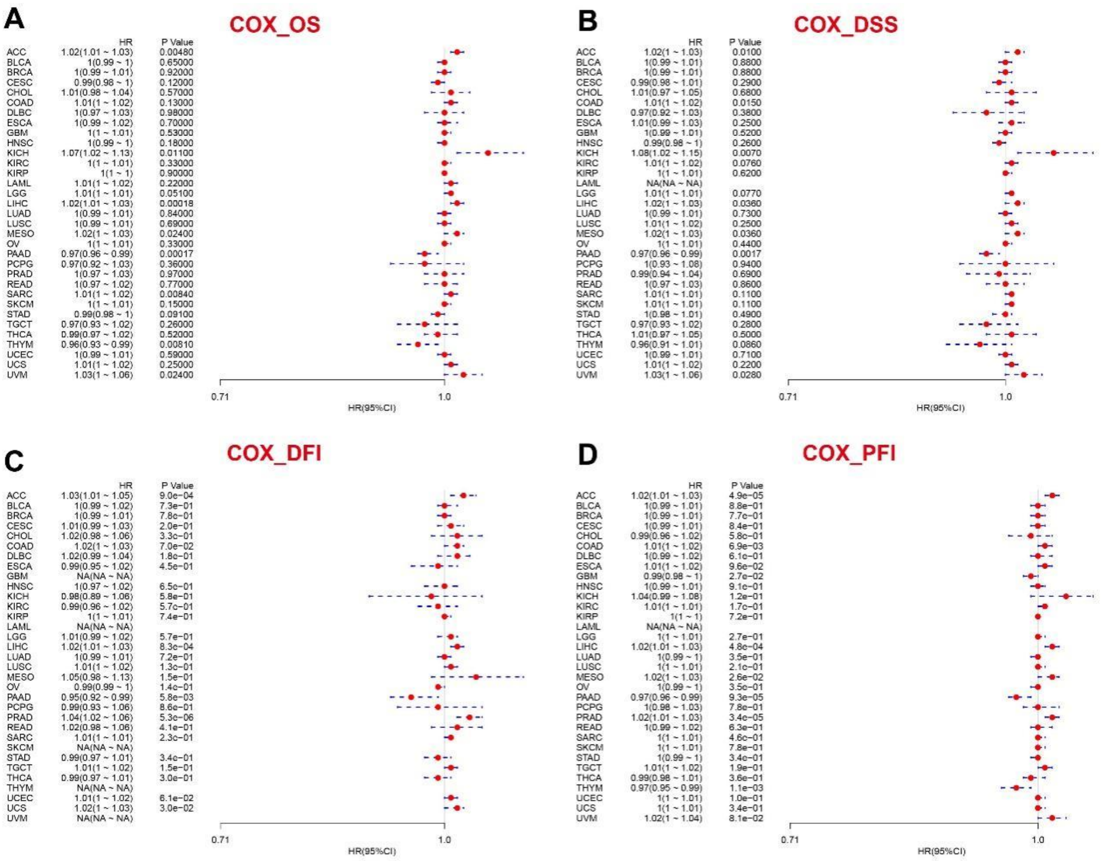

Figure S5 (A~D) Forest map showed the univariate cox regression results of RBM10 for OS (A), DSS(B), DFI(C) and PFI(D) in multiple cancers.

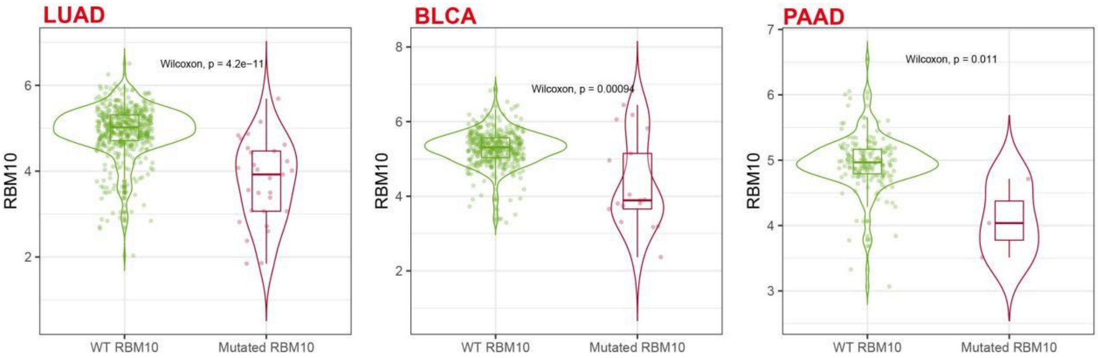

Figure S6 The expression level of RBM10 in the patients with RBM10 wild type (RBM10 WT) or RBM10 mutation (RBM10 Mut) of LUAD, BLCA and PAAD.

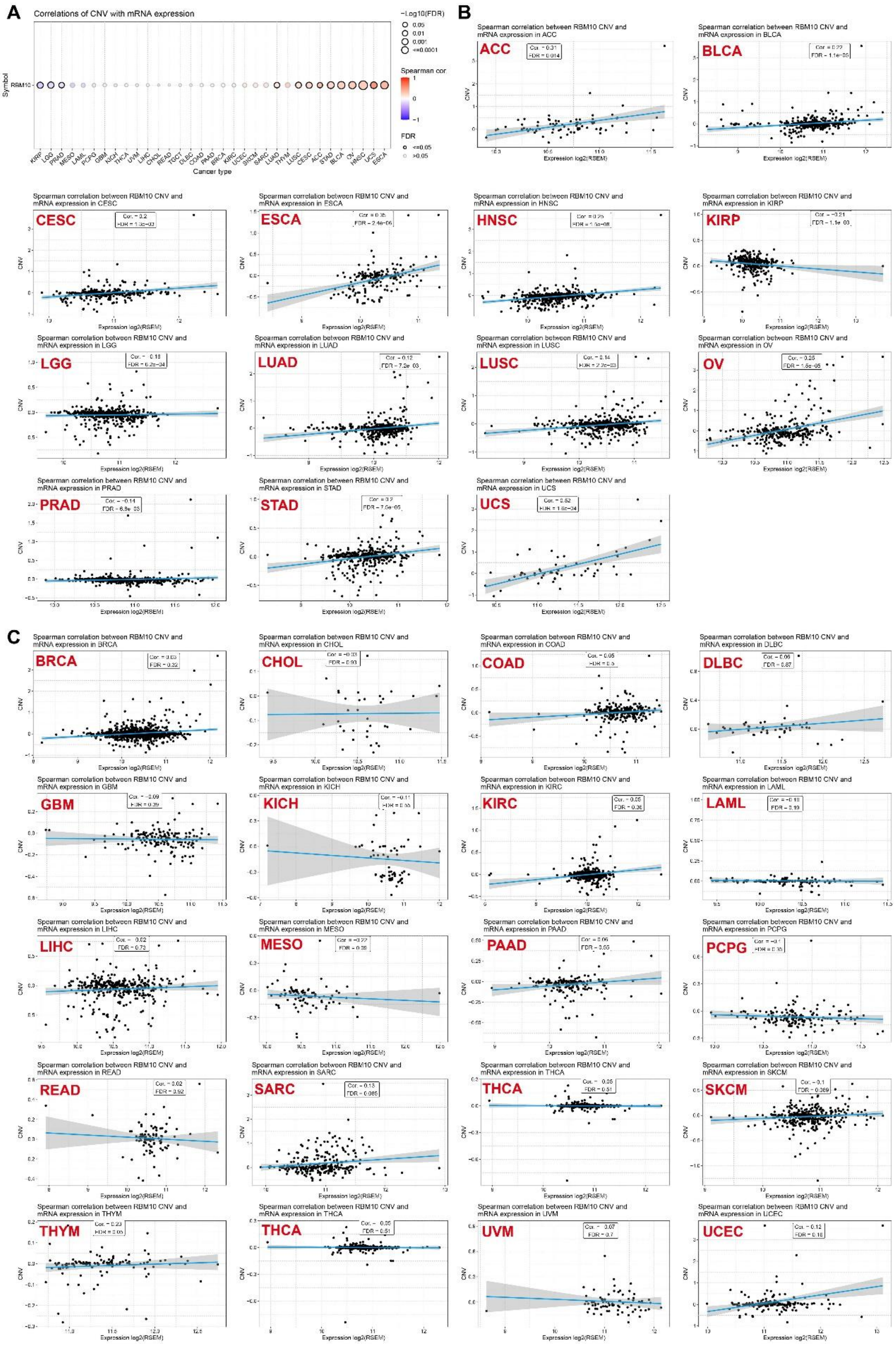

Figure S7 The correlation of RBM10 expression with CNV (A) The association between RBM10 mRNA expression and CNV in different cancers using GSCA. Blue

bubbles represented negative correlation, red bubbles represented positive correlation; and the darker the color, the higher the correlation. Bubble size was positively correlated with FDR. Black outline indicates FDR < 0.05. **(B)** A significant difference correlation between RBM10 mRNA levels and CNV in ACC, BLCA, CESC, ESCA, HNSC, KIRP, LGG, LUAD, LUSC, OV, PRAD, STAD and UCS. (**C**) There was no correlation between RBM10 mRNA expression and CNV among other human tumors.

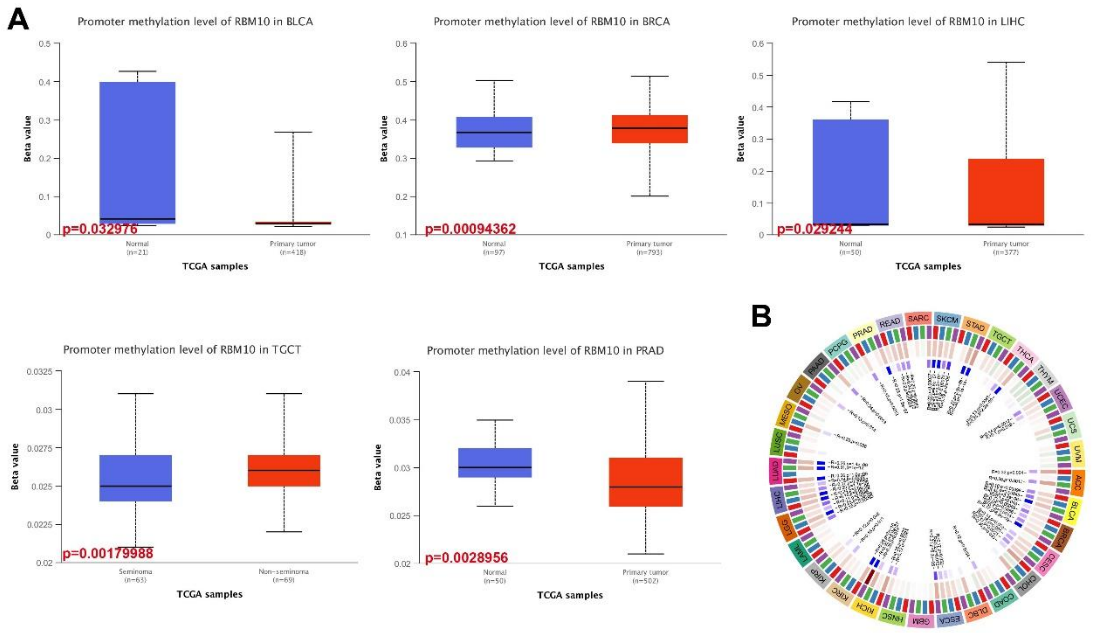

Figure S8 The promotor methylation level of RBM10 in various cancers (A) The promoter methylation level of RBM10 in BLCA, BRCA, LIHC, TGCT and PRAD. **(B)** The correlation between RBM10 expression and the level of DNMT genes in across different cancer types was investigated via SangerBox database. The red color: DNMT1, the blue color: DNMT2, the green color: DNMT3A, the purple: DNMT3B.

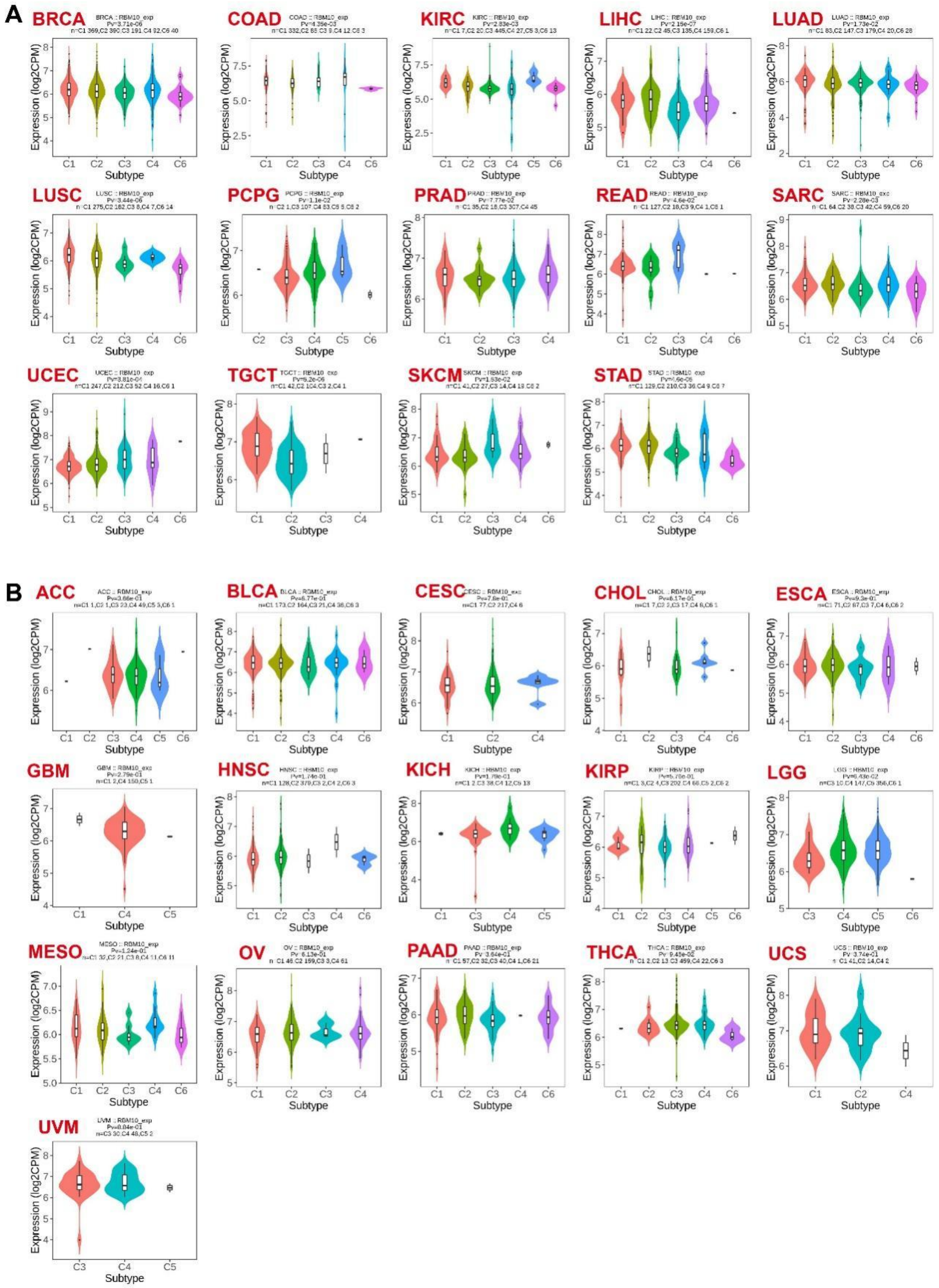

Figure S9 Relationship between RBM10 expression and different immune subtypes in human tumor (A) The violin diagram showed that RBM10 is significantly associated with different immune subtypes in BRCA, COAD, KIRC, LIHC, LUAD, LUSC, PCPG, PRAD, READ, SARC, UCEC, TGCT, SKCM and STAD using TISIDB. **(B)** There was not differ significantly between RBM10

expression and immune subtypes in ACC, BLCA, CESC, CHOL, ESCA, GBM, HNSC, KICH, KIRP, LGG, MESO, OV, PAAD, THCA, UCS and UVM.

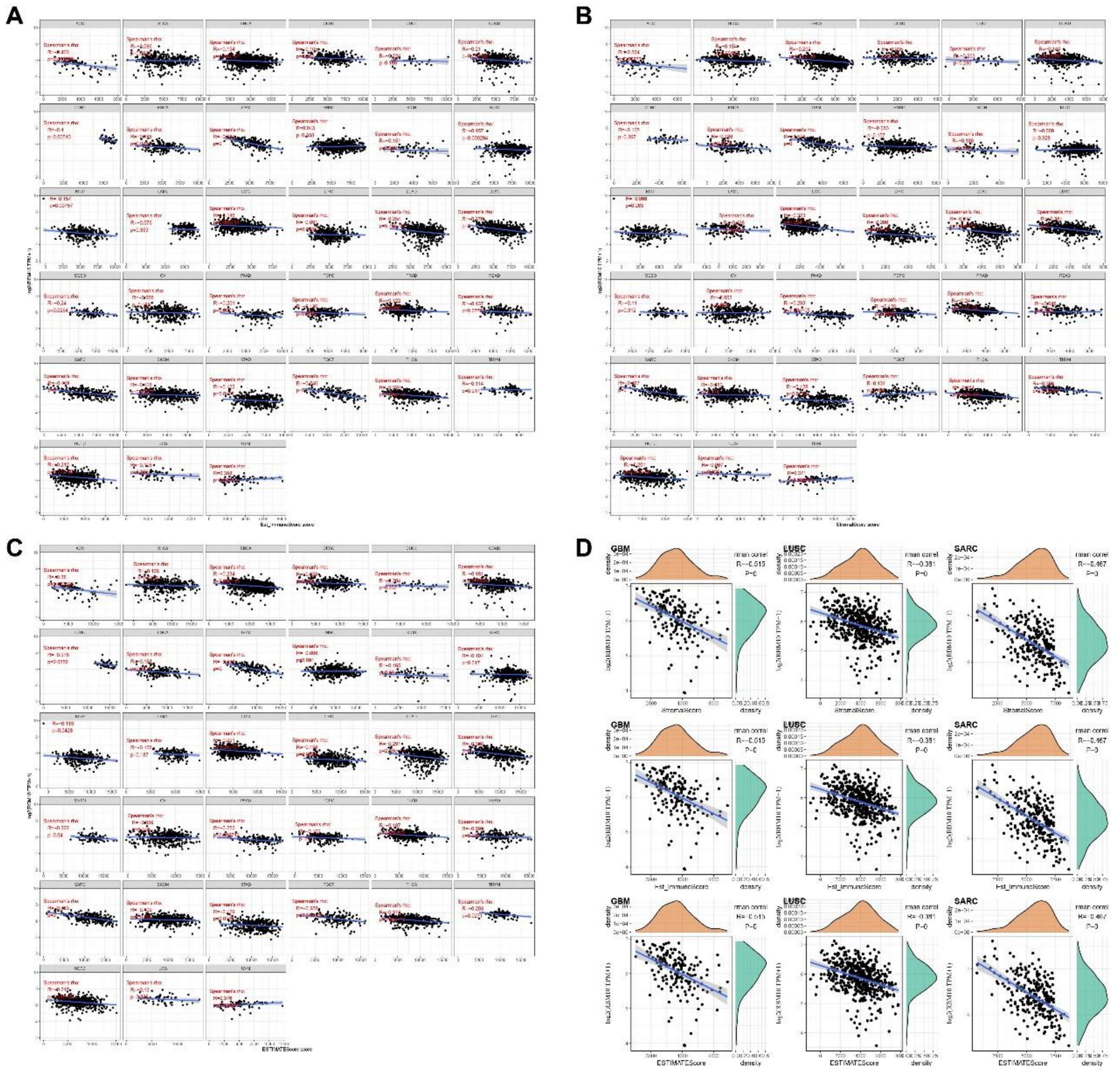

Figure S10 RBM10 expression was related to TME (A) The correlation of RBM10 expression with ImmuneScore in pan-cancer through Sangerbox. **(B)** The association between RBM10 level and StromalScore in different cancer types based on the SangerBox website. **(C)** The relationship of RBM10 expression with ESTIMATEScore across multiple cancer types using SangerBox. **(D)** The top three tumors that were most significantly related.

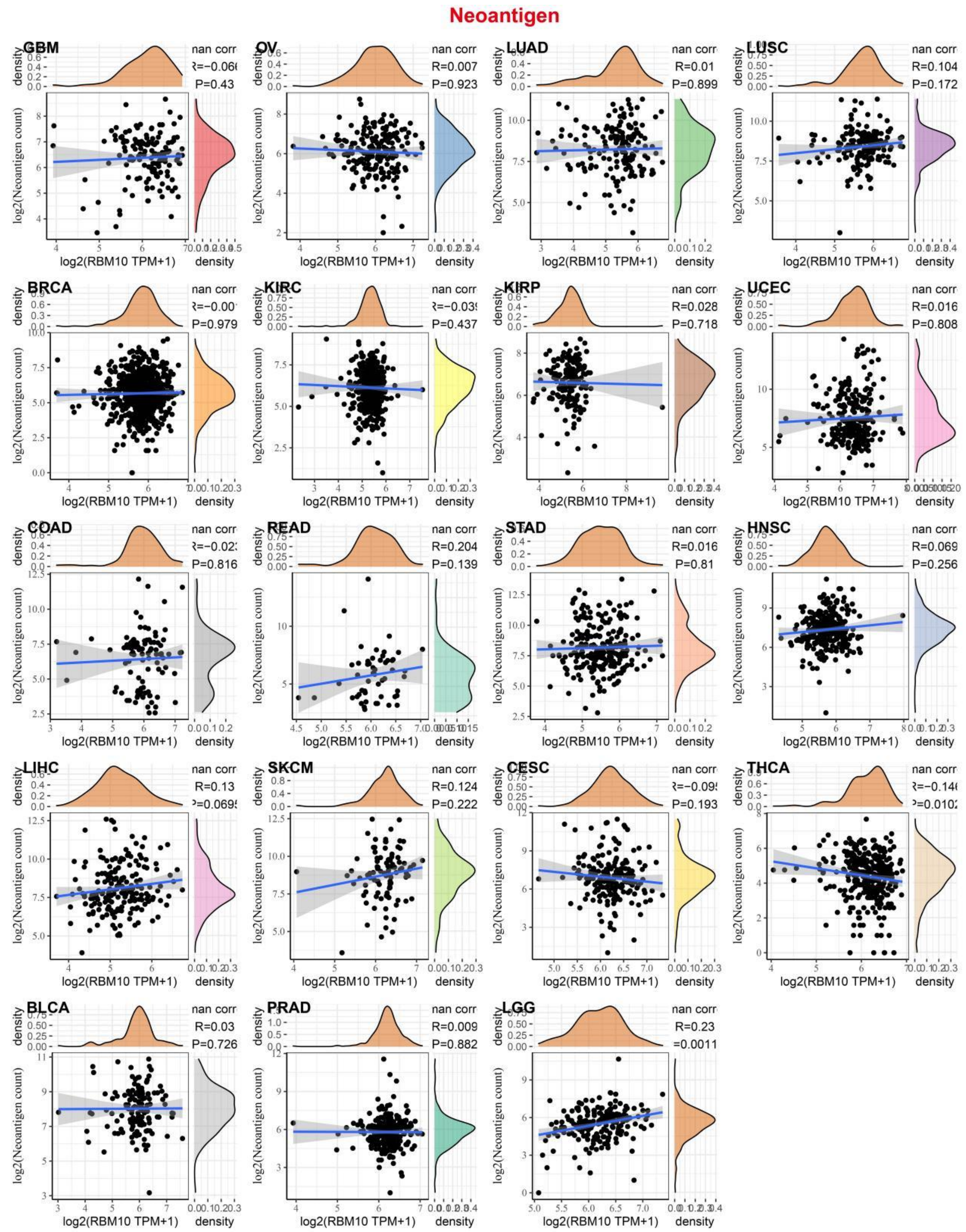

Figure S11 The relationship between RBM10 expression and neoantigens.

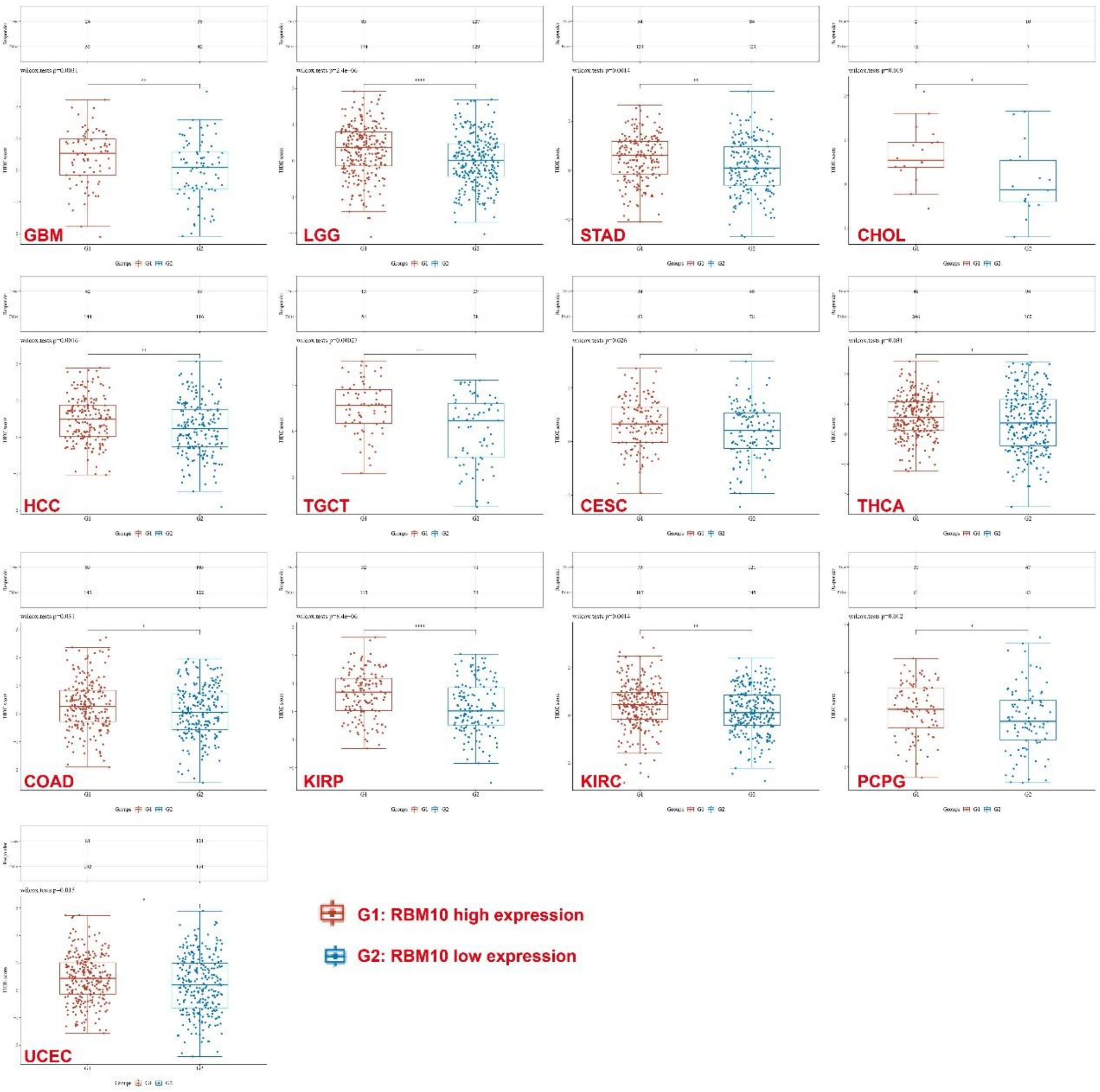

Figure S12 TIDE score of high and low RBM10 expression groups in GBM, TGCT, CESC, THCA, COAD, KIRP, KIRC, PCPG and UCEC. G1, RBM10 high expression: red color; G2, RBM10 low expression: blue color.

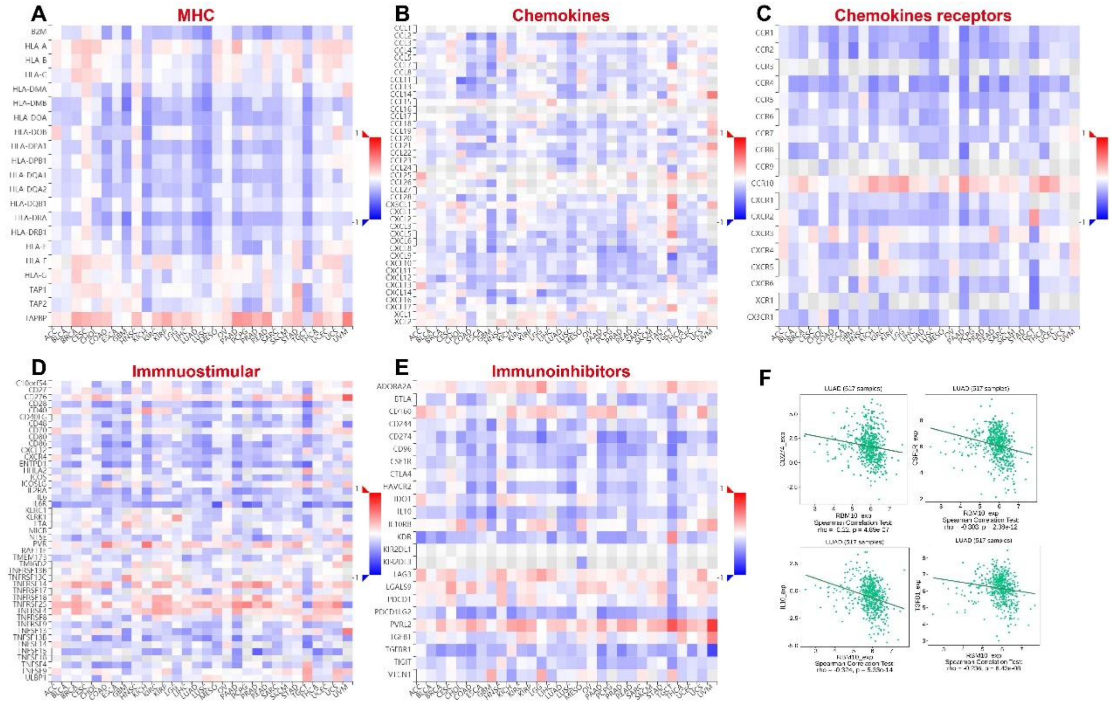

Figure S13 The correlation of RBM10 expression with immnuorelation-related genes (**A~E**) Heatmaps indicated the association of RBM10 expression with MHC(**A**), chemokines(**B**), chemokines recptors(**C**), immnuostimulars(**D**) and immuoinhibitors(**E**). (**F**) Scatter plots showed that RBM10 was negatively correlated with PD-L1(CD274), CSF1R, IL-10 and TGFB1 in LUAD.
