# Supplementary figures and images for: RBM10 Is a Biomarker Associated with Pan-Cancer Prognosis and Immune Infiltration: System Analysis Combined with In Vitro and Vivo Experiments
Source: Oxid Med Cell Longev. 2022 Nov 28;2022:7654937. doi: 10.1155/2022/7654937 (PMC11401663; doi:10.1155/2022/7654937)

Supplementary original western blots

Figure 8F

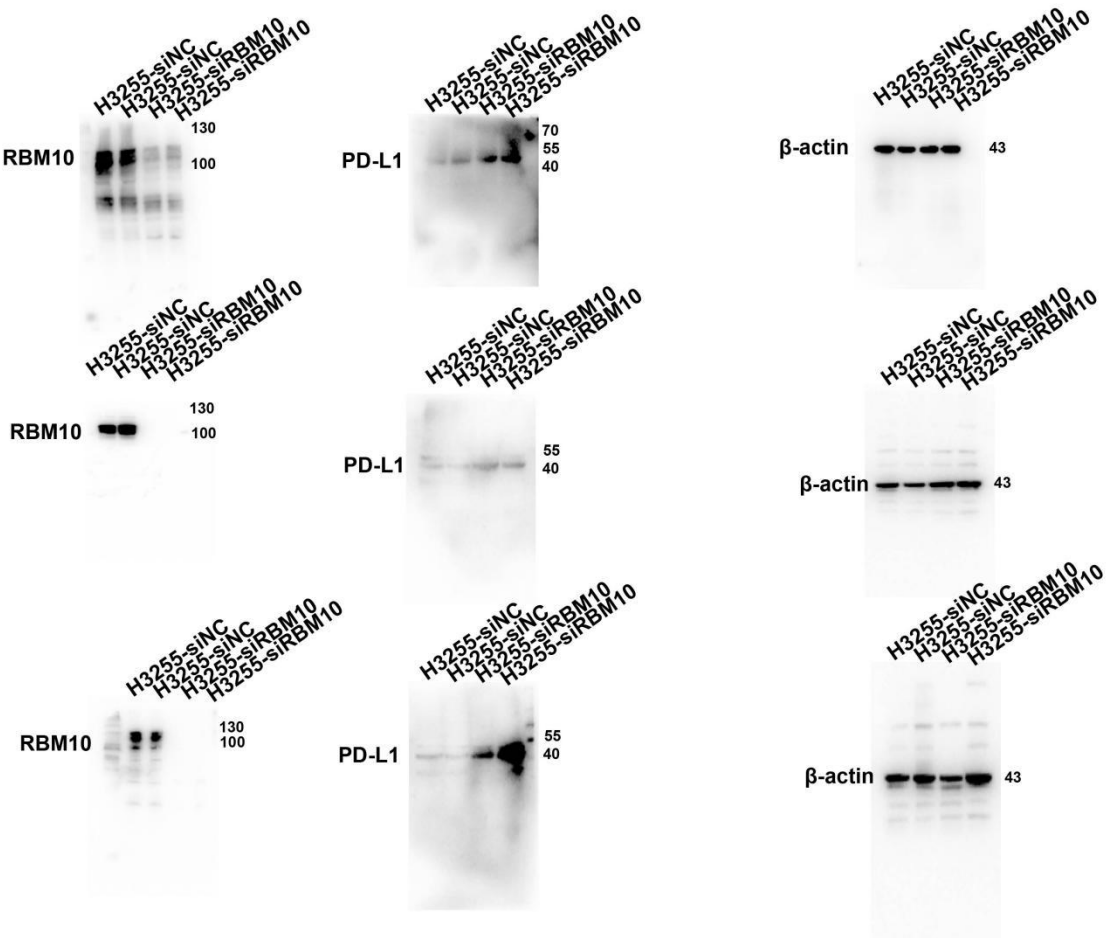

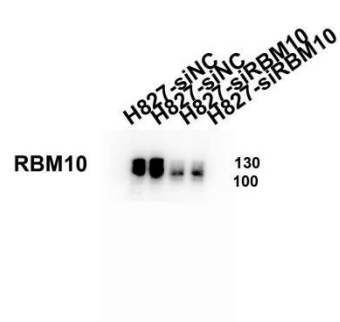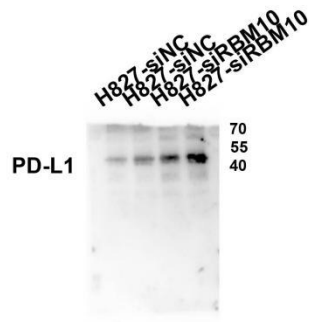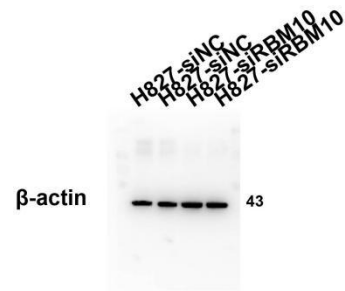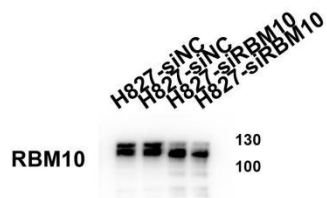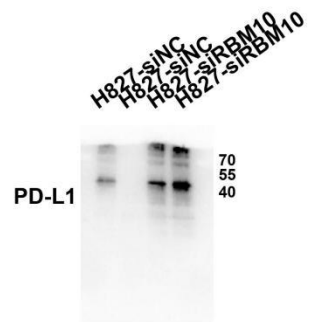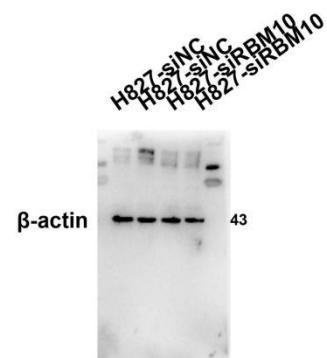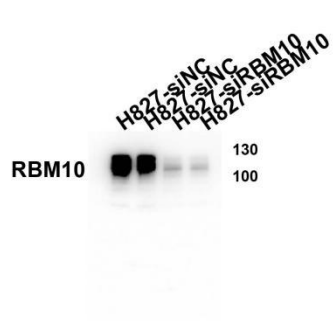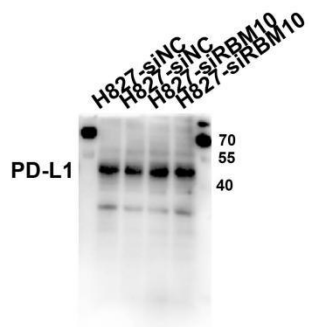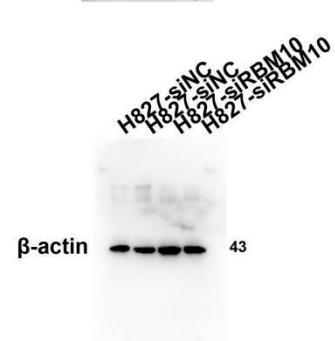

Figure 8G

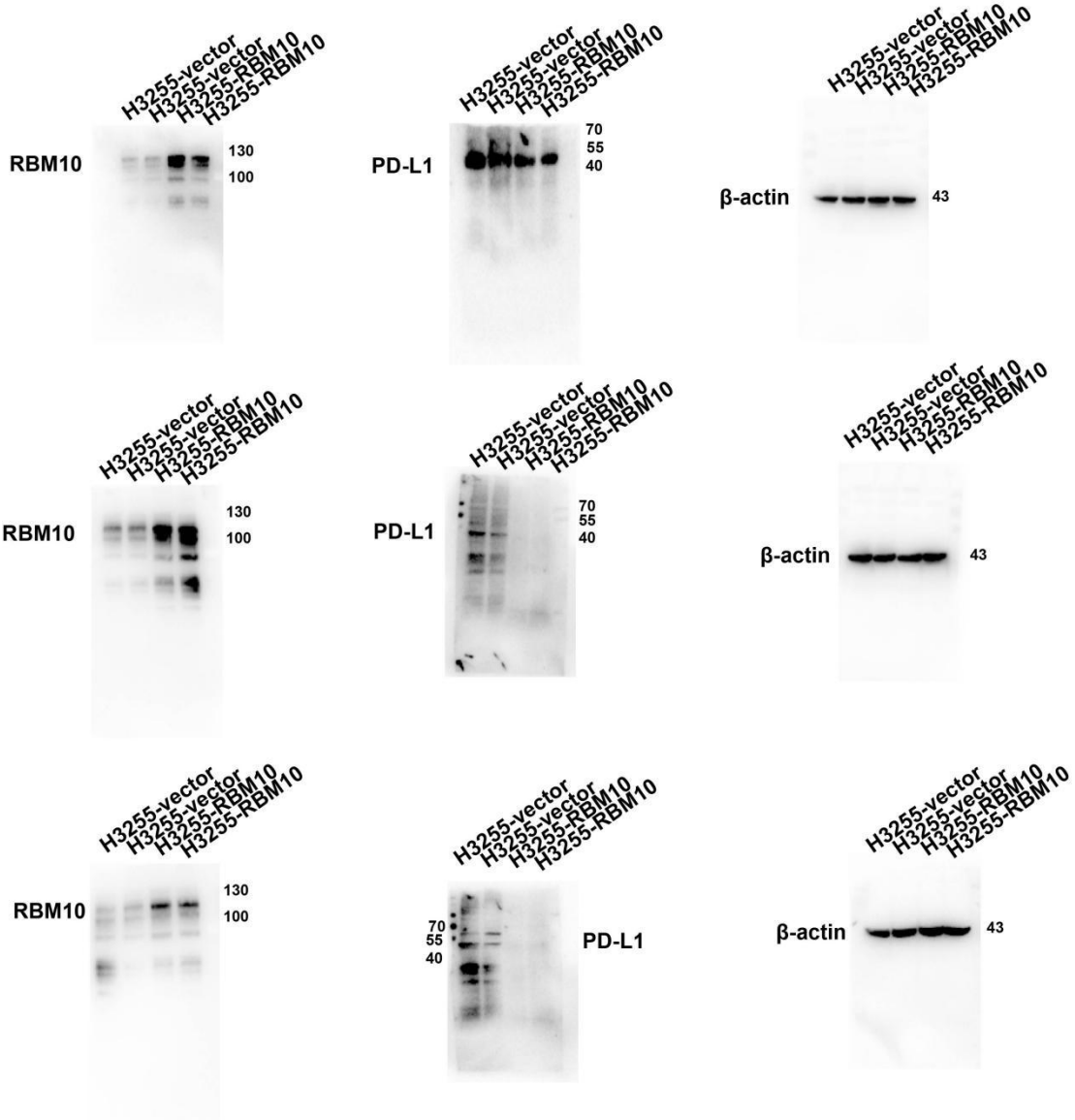

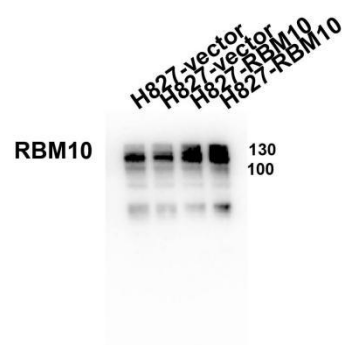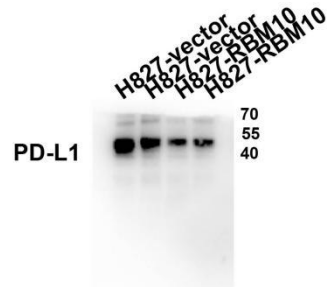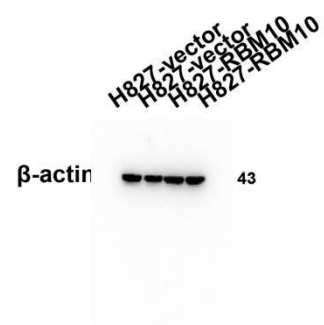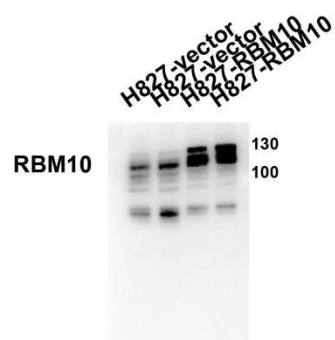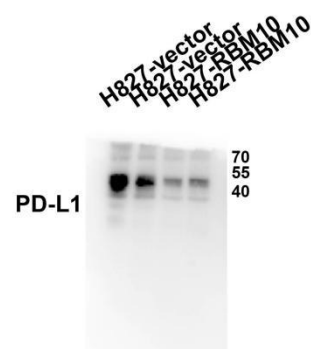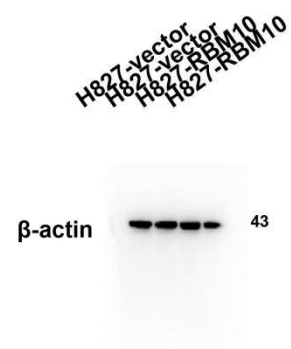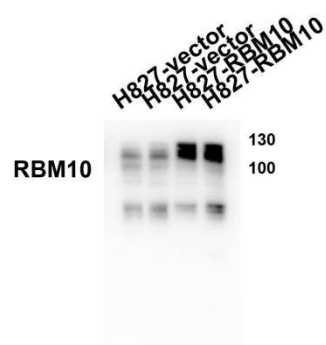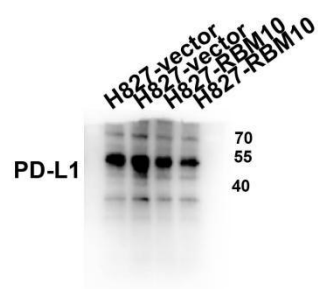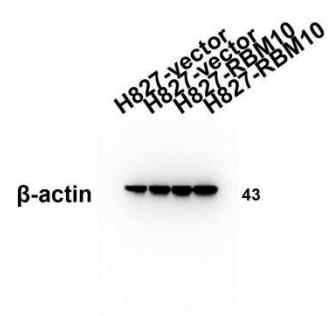

Figure 8H

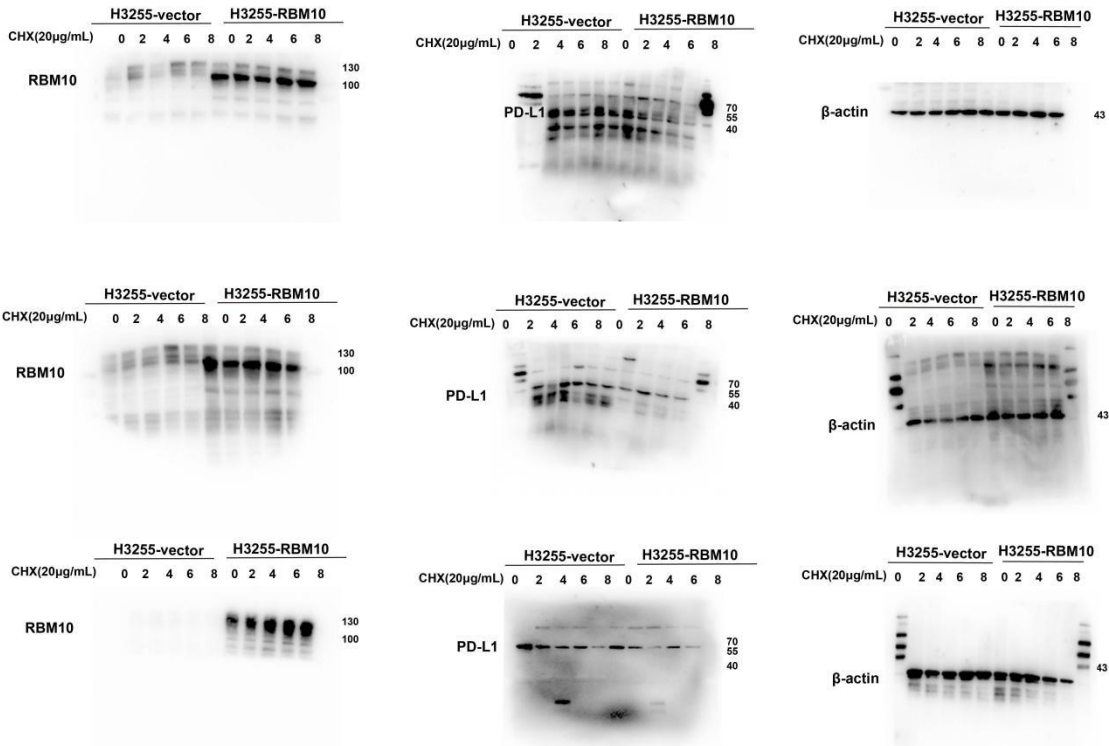

Figure 8I

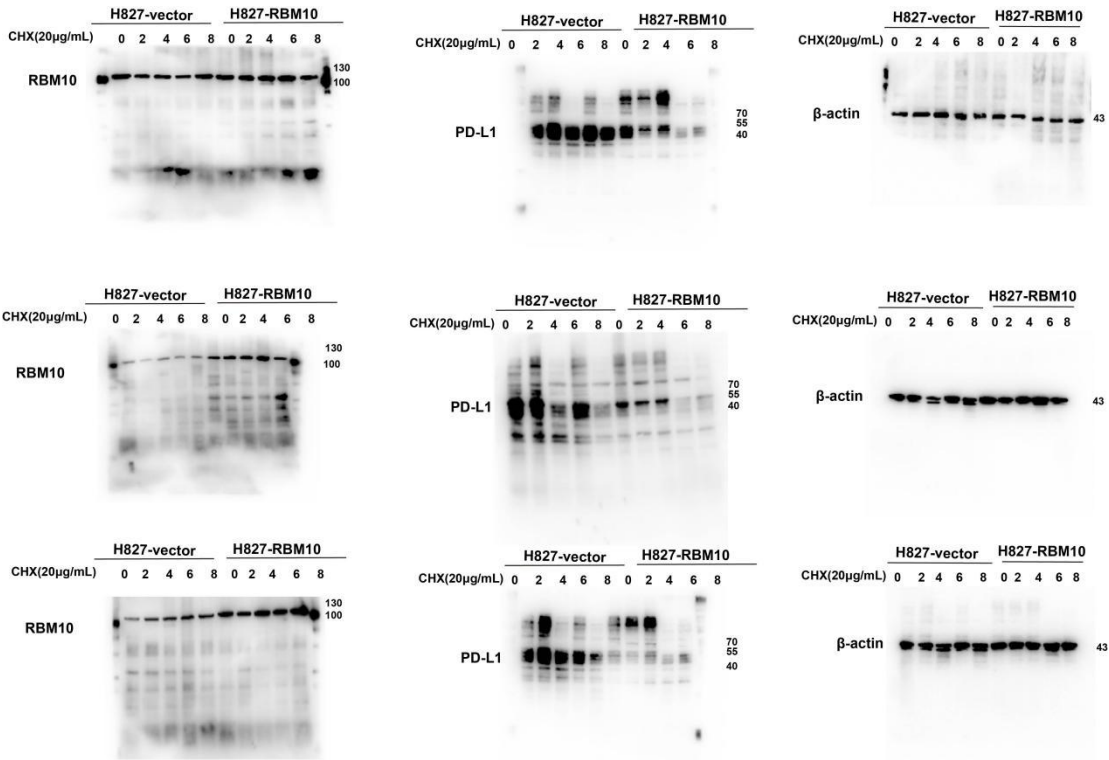

Figure 9E

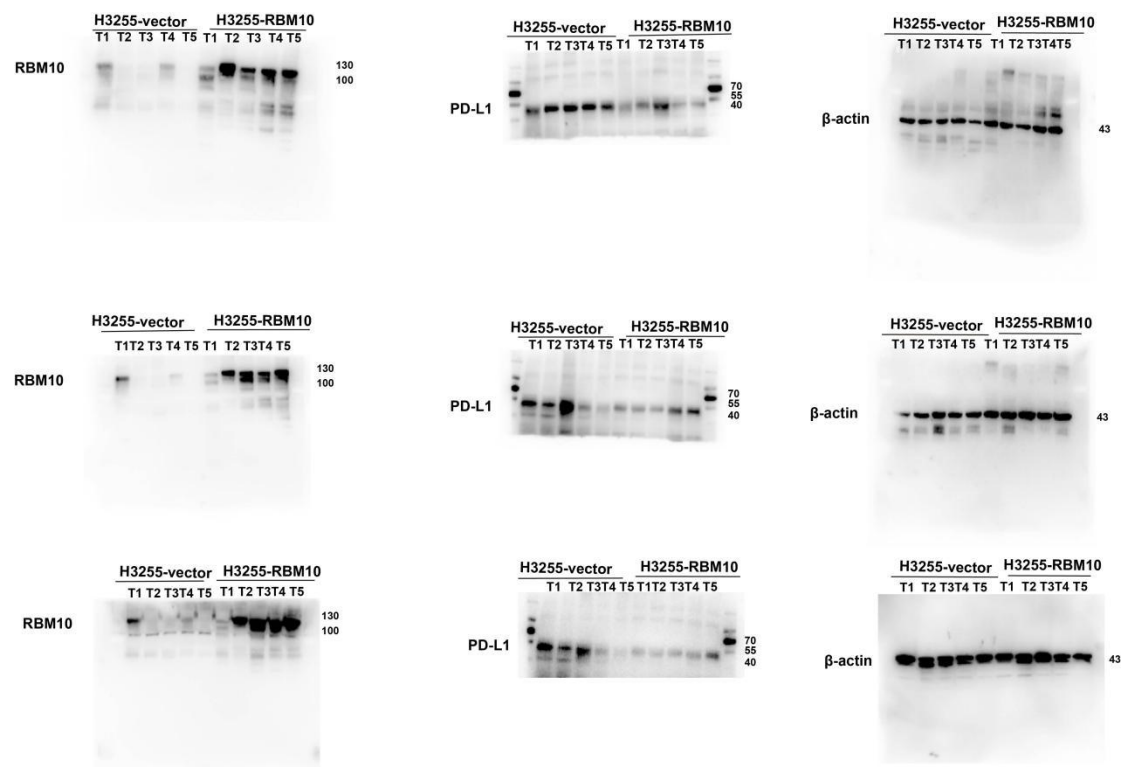

Supplement: Supplementary Materials — Figure S1: RBM10 mRNA expression in human normal tissues and different cancer cells. (A) RBM10 mRNA expression in various normal tissues was analyzed using the HPA/GTEx/FANTOM5 database. (B) The mRNA expression level of RBM10 in various cancer cells was analyzed from the data of the CCLE database. Figure S2: the protein expression of RBM10 in pan-cancer and a single-gene GO analysis of RBM10. (A) RBM10 protein level in different tumors and normal adjacent tissues through the CPTAC database. (B) SangerBox showed the positively association between RBM10 expression and each analysis result. Figure S3: the correlation between RBM10 and different molecular subtypes in pan-cancer. (A) The expression of RBM10 was significantly associated with molecular subtypes in BRCA, HNSC, KIRP, LGG, READ, OV, LUSC, and PRAD. (B) The RBM10 expression was not associated with molecular subtypes in ACC, COAD, ESCA, GBM, LIHC, PCPG, and SKCM. (C) The expression level of RBM10 was not associated with tumor stages in BLCA, BRCA, CESC, CHOL, COAD, DLBC, ESCA, HNSC, KIRC, KIRP, LUAD, LUSC, READ, STAD, TGCT, THCA, and UCEC. Figure S4: the prognosis analysis of RBM10 in tumors. (A, B) The correlation analysis of RBM10 expression with overall survival (OS, (A)) and relapse-free survival (RFS, (B)) in patients with different cancer types from the TCGA database of the Kaplan-Meier plotter. The median RBM10 expression was used to classify patients into high expression and low expression groups. p < 0.05 was statistically significant. Figure S5: (A–D) forest map showed the univariate Cox regression results of RBM10 for OS (A), DSS (B), DFI (C), and PFI (D) in multiple cancers. Figure S6: the expression level of RBM10 in the patients with RBM10 wild type (RBM10 WT) or RBM10 mutation (RBM10 Mut) of LUAD, BLCA, and PAAD. Figure S7: the correlation of RBM10 expression with CNV. (A) The association between RBM10 mRNA expression and CNV in different cancers using GSCA. Blue bubbles represented negative corr [file 7654937.f1.zip › Supplementary original Western blotts data.pdf]
